# Angular Regioselective Synthesis of Varied Functionalized Hexahydro-1,2,4-triazolo[4,3-*a*]quinazolin-9-ones and Their Antiproliferative Action †

**DOI:** 10.3390/molecules28093718

**Published:** 2023-04-25

**Authors:** Awad I. Said, Márió Gajdács, István Zupkó, Matti Haukka, Márta Palkó

**Affiliations:** 1 Institute of Pharmaceutical Chemistry, University of Szeged, Eötvös u. 6, H-6720 Szeged, Hungary; 2 Chemistry Department, Faculty of Science, Assiut University, Assiut 71516, Egypt; 3 Institute of Pharmacodynamics and Biopharmacy, University of Szeged, Eötvös u. 6, H-6720 Szeged, Hungary; gajdacs.mario@szte.hu (M.G.); zupko.istvan@szte.hu (I.Z.); 4 Department of Chemistry, University of Jyväskulä, FIN-40014 Jyväskulä, Finland; matti.o.haukka@jyu.fi

**Keywords:** regioselective synthesis, hydrazonoyl chloride, [1,2,4]triazolo[4,3-*a*]quinazolin-9-ones, antitumor action

## Abstract

New 2-thioxopyrimidin-4-ones capable of participating in regioselective reactions with functionally diverse hydrazonoyl chlorides towards angular regioisomers, rather than linear ones, were designed and synthesized to form stereoisomeric *cis*- and *trans*-hexahydro [1,2,4]triazolo[4,3-*a*]quinazolin-9-ones to be tested as antitumor candidates. The angular regiochemistry of the products was verified through crystallographic experiments and NMR studies. In addition, the regioselectivity of the reaction was found to be independent of the stereochemistry of the used 2-thioxopyrimidin-4-one. Only compound **4c** demonstrated satisfactory growth inhibition against all the cancer cells used among all the produced drugs.

## 1. Introduction

Hydrazonoyl halides have attracted the attention of chemists in organic synthesis, since they exhibit valuable applicability as precursors for the synthesis of various heterocyclic compounds, such as pyrazoles [1], thiazoles [2,3], imidazoles [4], triazoles [5,6], thiadiazoles [7,8], and tetrazines [9].

Reactions of hydrazonoyl halides with 2-thioxopyrimidin-4-ones lead to the formation of 1,2,3-triazoles, which have a wide range of applications as synthetic intermediates and pharmaceuticals [10,11,12,13]. Numerous 1,2,4-triazoles with medicinal potential have been identified, including HIV inhibitors [14,15,16], antimicrobial drugs [17], antitumor potentials [18], and kinase inhibitors [19,20]. These reactions are regioselective and controlled by electronic factors rather than steric properties. Namely, in the reaction of the two possible regioisomeric 1,2,3-triazoles, the formation of the linear regioisomer is favored by the presence of the C=C bond in 2-thioxopyrimidin-4-ones [21,22,23,24,25]. The formation of the angular regioisomer, in turn, will be favored if the substrate does not contain the C=C bond, as demonstrated in our previous works [26,27].

Cancer remains the second leading cause of mortality in both industrialized and developing nations, despite the great advancements in cancer therapy that have increased the cure rates for a variety of malignancies [28]. Chemotherapy is one of the main treatment options for cancer patients. The toxicity and drug resistance [29] of the existing chemotherapeutics, however, restrict their use in the treatment of cancer patients. Therefore, the development of safe chemotherapy drugs with anticancer properties is urgently needed. The anticancer efficacy of compounds bearing the 1,2,4-triazolo[4,3-*a*]pyrimidine scaffold is well established [30,31,32]. Hence, we were motivated to prepare new 1,2,4-triazolo[4,3-*a*]pyrimidines based on cyclohexene and to investigate their antiproliferative actions.

Continuing our research, we designed and synthesized new 2-thioxopyrimidin-4-ones achieving regioselective reactions with functionally diverse hydrazonoyl chlorides **3a**–**h**. These new 2-thioxopyrimidin-4-ones are *cis* and *trans* stereoisomers of cyclohexene-condensed 2-thioxopyrimidin-4-ones **1** and **2**. The product molecules can be reacted further to form novel angular *cis*- and *trans*-1,2,4-triazoles **4a**–**h** and **5a**–**h** with various functionalities which have the C=C bond for further derivatization. X-ray and NMR investigations were employed to establish the stereochemistry of the compounds. Furthermore, the antiproliferative action of the prepared compounds was also examined.

## 2. Results and Discussion

**Scheme 1:**
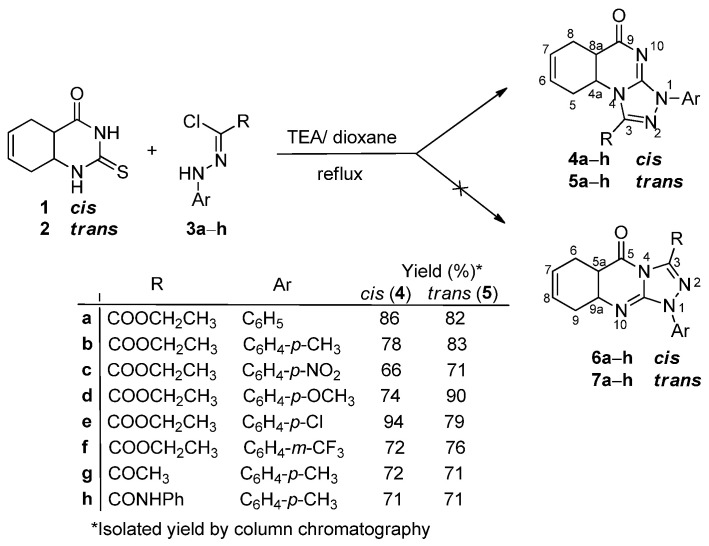
Synthesis of angular [1,2,4]triazolo[4,3-*a*]quinazolinones **4a**–**h** and **5a**–**h.** Reagents and conditions: **1** or **2** (0.06 mmol), **3a**–**h** (0.06 mmol), TEA (100 μL), dioxane (10 mL), reflux 6–8 h.

**Scheme 2:**
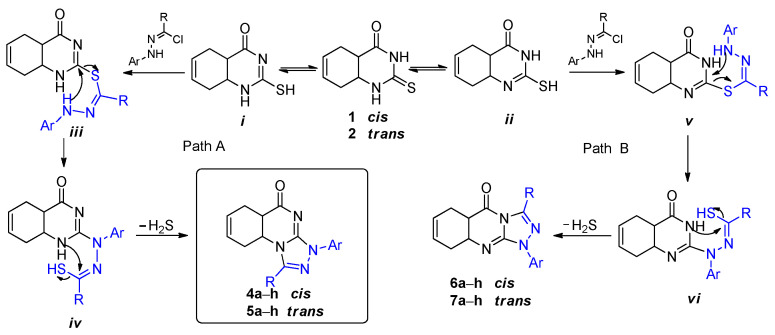
Proposed pathway for forming angular and linear regioisomers.

**Figure 1:**
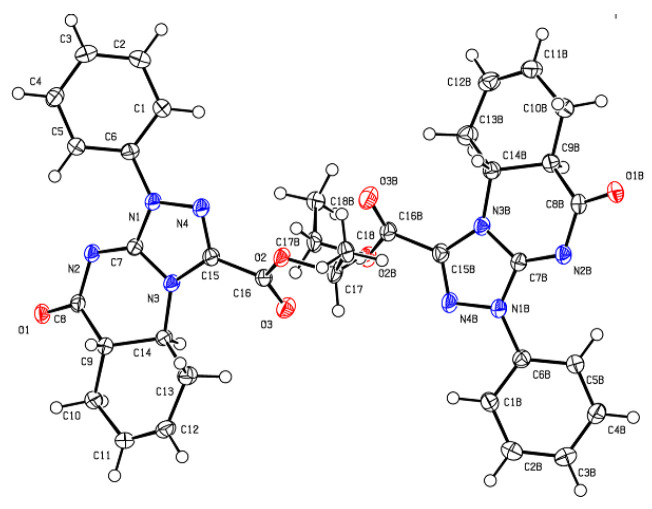
TELP image of **5a** at 50% probability level.

The cyclohexene-based 2-thioxopyrimidin-4-one **1** and its *trans* stereoisomer **2** precursor molecules were synthesized according to the literature [33]. The reactions of thioxopyrimidinones **1** or **2** with functionally diverse hydrazonoyl chlorides **3a–h** were performed in dioxane as a solvent using triethylamine as a basic additive under reflux conditions for 6–8 h (Scheme 1). As shown in Scheme 2, the reaction proceeds through either path A or B, depending on the tautomeric structure ***I*** or ***ii*** that directs the reaction via *S*-alkylation to form *S*-alkylated intermediates ***iii*** or ***v***, respectively. These then undergo Smiles rearrangement [34], yielding intermediates ***iv*** or ***vi***, followed by cyclization through the elimination of H_2_S to afford angular regioisomers **4a**–**h** and **5a**–**h** or linear ones **6a**–**h** and **7a**–**h**, respectively. Because of the conjugation of the C=N and C=O bonds, the tautomeric form ***i*** is favored in comparison to ***ii***. Consequently, the reactions proceeded through path A, which accounts for the regioselectivity of the reactions towards the angular isomers **4a**–**h** and **5a**–**h** rather than the linear ones **6a**–**h** and **7a**–**h**. This regioselectivity was confirmed using a variety of tests, including chromatography, ^1^H-NMR ^13^C-NMR spectroscopy, and X-ray crystallographic analysis. TLC, following the reaction, indicated the formation of only a single product. The ^1^H-NMR spectra (Supplementary Materials) showed the resonances of only a single isomer. In addition, in the case of hydrazonoyl chlorides **3a**–**f**, the signal of the methylene (CH_2_) moiety of the ester functional group appeared to have higher multiplicity than expected (quartet). This is due to the proximity of the ester group to the cyclohexene moiety in the angular regioisomer. The ^13^C-NMR spectrum (Supplementary Materials) exhibited the resonance of the carbonyl carbon (CO) of the pyrimidinone ring at almost 176 ppm, which is in accordance with the reported values of structurally related carbonyl carbon atoms in the literature [35]. In these compounds, where the carbonyl carbon of the pyrimidinone residue in the angular structure is adjacent to the sp^2^-hybridized nitrogen (releasing less electrons), the carbonyl group is less shielded, and it resonates at 170–176 ppm. In contrast, the nitrogen atom in the linear structure is sp^3^-hybridized (releasing more electrons), and consequently the carbonyl group is more shielded and resonates at lower δ values (160–165 ppm). Finally, X-ray crystallographic analysis of **5a** (Figure 1) provided indisputable evidence of the angular stereochemistry of the product.

As concerns the antiproliferative properties of the compounds thus prepared, none of them proved to be comparable with the reference agent cisplatin (Table 1). The most active analog was **4c**, eliciting 30–50% growth inhibition at 30 μM against all the cancer cells used. Incubation with compound **5h** resulted in cell growth inhibition above 30% against 3 cell lines. Though no clear tendency was observed concerning the role of stereochemistry in the antiproliferative activity of the compounds, our results indicate that the *cis* arrangement of the *p*-nitrophenyl substituent on the triazole ring may be attractive for anticancer drug candidates with a similar scaffold. Treatment with compounds **4d**, **4g**, and **4h** resulted in less than 20% growth inhibition at the higher concentration against only a single cell line. All other molecules (**4a**, **4b**, and **5a**) elicited no relevant antiproliferative action (i.e., less than 10% growth inhibition) against the tested cancer cell lines.

## 3. Materials and Methods

### 3.1. General Methods

NMR characterization of the product compounds was carried out in CDCl_3_ at room temperature (500.20 MHz for ^1^H-NMR, 125.62 MHz for ^13^C-NMR) using a Bruker AV NEO Ascend 500 spectrometer with a Double-Resonance Broad-Band Probe (Bruker Biospin, Karlsruhe, Germany). As an internal standard, tetramethylsilane (TMS) was used. Thin-layer chromatography (TLC) was conducted to monitor the reaction progress (aluminum sheets, silica gel coating (POLYGRAM^®^SIL G/UV254, Merck, Darmstadt, Germany) with evaluations upon UV illumination. Melting points were measured using Hinotek-X4 micro melting point equipment (Hinotek, Ningbo, China). The HRMS flow injection study was carried out using a Thermo Scientific Q Exactive Plus hybrid quadrupole-Orbitrap mass spectrometer linked to a Waters Acquity I-Class UPLCTM (Thermo Fisher Scientific, Waltham, MA, USA) (Waters, Manchester, UK).

The synthesis of *cis*- and *trans*-thioxopyrimidinones (**1** and **2**) was carried out by transforming the corresponding amino esters, as described in the literature [36,37]. The synthesis of hydrazonoyl chlorides **2a**–**h** was performed in accordance with the previously described methods [38,39].

### 3.2. Synthesis of Cis- and Trans-Hexahydro [1,2,4]triazolo[4,3-a]quinazolin-9(1H)-ones ***4a***–***h*** and ***5a***–***h***

Cyclohexene-condensed 2-thioxopyrimidin-4-one **1** or **2** (0.6 mmol) and hydrazonoyl chlorides (**3a**–**h**) were treated under reflux conditions for 6–8 h in the presence of 100 μL triethylamine (TEA) and 10 mL of dioxane. The reaction was monitored using TLC (*n*-hexane/EtOAC = 1:1) until it was completed. After the evaporation of the solvent under reduced pressure, the residue dissolved in CHCl_3_ (20 mL) was extracted with water (three times, 10 mL). Then, the solution was dried (Na_2_SO_4_), and the residue, after evaporation of the solvent under reduced pressure, was purified using column chromatography with an *n*-hexane/EtOAc eluent ratio of 2:1.

(4aS*,8aR*)-Ethyl 9-oxo-1-phenyl-1,4a,5,8,8a,9-hexahydro [1,2,4]triazolo[4,3-*a*]quinazoline-3-carboxylate (**4a**): 0.17 g (86%), white crystals, m.p. 232–234 °C. ^1^H NMR (500 MHz, CDCl_3_) δ 8.04 (dd, *J* = 8.7, 1.0 Hz, 2H), 7.46 (t, *J* = 8.0 Hz, 2H), 7.34 (t, *J* = 8.0 Hz, 1H), 5.88 (dd, *J* = 9.3, 4.4 Hz, 1H), 5.70–5.55 (m, 1H), 4.60–4.43 (m, 2H, CH_3_*CH_2_*), 4.38 (ddd, *J* = 13.6, 9.7, 5.1 Hz, 1H), 3.34–3.21 (m, 1H), 2.84–2.71 (m, 1H), 2.62 (ddd, *J* = 13.4, 11.2, 4.9 Hz, 1H), 2.42–2.21 (m, 2H), 1.47 (t, *J* = 7.1 Hz, 3H, *CH_3_*CH_2_). ^13^C NMR (126 MHz, CDCl_3_) δ 176.4, 157.1, 153.5, 138.8, 136.2, 129.2, 127.8, 127.3, 122.1, 121.9, 63.8, 54.8, 39.1, 32.2, 26.3, 14.1. HRMS-ESI [M+H] + *m*/*z* calcd for C_18_H_19_N_4_O_3_: 339.14517, found: 339.14441.

(4aS*,8aR*)-Ethyl 9-oxo-1-(*p*-tolyl)-1,4a,5,8,8a,9-hexahydro [1,2,4]triazolo[4,3-*a*]quinazoline-3-carboxylate (**4b**): 0.17 g (78%), white crystals, m.p. 212–215 °C. ^1^H NMR (500 MHz, CDCl_3_) δ 7.93–7.85 (m, 2H), 7.25 (d, *J* = 8.2 Hz, 2H), 5.92–5.83 (m, 1H), 5.64 (dd, *J* = 9.7, 5.7 Hz, 1H), 4.65–4.42 (m, 2H, CH_3_*CH_2_*), 4.36 (ddd, *J* = 13.7, 9.7, 5.1 Hz, 1H), 3.38–3.20 (m, 1H), 2.38 (s, 2H), 2.31 (qdd, *J* = 9.4, 4.5, 2.2 Hz, 2H), 1.47 (t, *J* = 7.1 Hz, 3H, *CH_3_*CH_2_). ^13^C NMR (126 MHz, CDCl_3_) δ 176.3, 157.1, 153.4, 138.7, 137.9, 133.7, 129.7, 127.3, 122.1, 121.8, 63.7, 54.8, 39.1, 32.2, 26.3, 21.1, 14.1. HRMS-ESI [M+H] + *m*/*z* calcd for C_19_H_21_N_4_O_3_: 353.16082, found: 353.16007.

(4aS*,8aR*)-Ethyl 9-oxo-1-(4-nitrophenyl)-1,4a,5,8,8a,9-hexahydro-[1,2,4]triazolo[4,3-*a*]quinazoline-3-carboxylate (**4c**): 0.15 g (66%), white crystals, m.p. 257–259 °C. ^1^H NMR (500 MHz, CDCl_3_) δ 8.46 (d, *J* = 8.5 Hz, 2H), 8.32 (d, *J* = 8.9 Hz, 2H), 5.89 (br, 1H,), 5.65 (br, 1H), 4.55 (m, *J* = 15.2, 7.4 Hz, 2H, CH_3_*CH_2_*), 4.40 (m, 1H), 3.25 (d, *J* = 15.0 Hz, 1H), 2.78 (d, *J* = 17.4 Hz, 1H), 2.64 (dd, *J* = 16.3, 7.4 Hz, 1H), 2.49–2.19 (m, 2H), 1.50 (t, *J* = 6.9 Hz, 3H, *CH_3_*CH_2_). ^13^C NMR (126 MHz, CDCl_3_) δ 176.2, 156.8, 153.7, 145.9, 141.2, 139.6, 127.2, 124.8, 122.0, 121.2, 64.1, 54.9, 39.1, 32.1, 26.1, 14.0. HRMS-ESI [M+H] + *m*/*z* calcd for C_18_H_18_N_5_O_5_: 384.13025, found: 384.12941.

(4aR*,8aS*)-Ethyl 9-oxo-1-(4-methoxyphenyl)-1,4a,5,8,8a,9-hexahydro-[1,2,4]triazolo[4,3-*a*]quinazoline-3-carboxylate (**4d**): 0.16 g (74%), white crystals, m.p. 221–223 °C. ^1^H NMR (500 MHz, CDCl_3_) δ 8.01–7.78 (m, 2H), 7.08–6.77 (m, 2H), 5.99–5.75 (m, 1H), 5.64 (dd, *J* = 9.0, 5.2 Hz, 1H), 4.64–4.41 (m, 2H, CH_3_*CH_2_*), 4.37 (ddd, *J* = 13.6, 9.6, 5.2 Hz, 1H), 3.83 (s, 3H,OCH_3_), 3.40–3.17 (m, 1H), 2.83–2.70 (m, 1H), 2.61 (ddd, *J* = 13.4, 11.1, 5.0 Hz, 1H), 2.49–2.18 (m, 2H), 1.47 (t, *J* = 7.2 Hz, 3H, *CH_3_*CH_2_). ^13^C NMR (126 MHz, CDCl_3_) δ 176.3, 159.1, 157.1, 153.3, 138.7, 129.1, 127.3, 123.7, 122.1, 114.3, 63.7, 55.6, 54.9, 39.1, 32.2, 26.3, 14.1. HRMS-ESI [M+H] + *m*/*z* calcd for C_19_H_21_N_4_O_4_: 369.15573, found: 369.15494.

(4aR*,8aS*)-Ethyl 9-oxo-1-(4-chlorophenyl)-1,4a,5,8,8a,9-hexahydro [1,2,4]triazolo[4,3-*a*]quinazoline-3-carboxylate (**4e**): 0.21 g (94%), white crystals, m.p. 238–240 °C. ^1^H NMR (500 MHz, CDCl_3_) δ 8.12–8.02 (m, 2H), 7.46–7.37 (m, 2H), 6.01–5.77 (m, 1H), 5.75–5.50 (m, 1H), 4.62–4.42 (m, 2H, CH_3_*CH_2_*), 4.37 (ddd, *J* = 13.7, 9.7, 5.2 Hz, 1H), 3.40–3.11 (m, 1H), 2.87–2.69 (m, 1H), 2.62 (ddd, *J* = 13.3, 11.1, 5.0 Hz, 1H), 2.49–2.05 (m, 2H), 1.48 (t, *J* = 7.1 Hz, 3H, *CH_3_*CH_2_). ^13^C NMR (126 MHz, CDCl_3_) δ 156.9, 153.5, 138.9, 134.8, 133.3, 129.3, 127.3, 122.7, 122.1, 63.9, 54.9, 39.1, 32.1, 26.3, 14.0. HRMS-ESI [M+H] + *m*/*z* calcd for C_18_H_18_ClN_4_O_3_: 373.10619, found: 373.10548.

(4aR*,8aS*)-Ethyl 9-oxo-1-(3-(trifluoromethyl)phenyl)-1,4a,5,8,8a,9-hexahydro [1,2,4]triazolo[4,3-*a*]quinazoline-3-carboxylate (**4f**): 0.17 g (72%), white crystals, m.p. 167–169 °C. ^1^H NMR (500 MHz, CDCl_3_) δ 8.54–8.46 (m, 1H), 8.23 (s, 1H), 7.63–7.56 (m, 2H), 5.91–5.86 (m, 1H), 5.65 (dd, *J* = 8.9, 5.0 Hz, 1H), 4.61–4.48 (m, 2H, CH_3_CH_2_), 4.43–4.36 (m, 1H), 3.30–3.23 (m, 1H), 3.10 (qd, *J* = 7.3, 5.0 Hz, 1H), 2.81–2.73 (m, 1H), 2.64 (ddd, *J* = 13.4, 11.1, 5.0 Hz, 1H), 2.39–2.27 (m, 2H), 1.49 (t, *J* = 7.1 Hz, 3H, CH_3_CH_2_).^13^C NMR (126 MHz, CDCl_3_) δ 176.2 (C=O), 156.9(C=O), 153.6(C), 139.2(C), 136.71(C), 131.7 (q, *J* = 33 Hz, C-CF_3_) 129.9 (CH), 127.2(CH), 124.9(CH), 124.2 (q, *J* = 3.7 Hz, CHCCF_3_), 123.5 (q, *J* = 230 Hz, CF_3_) 122.0(CH), 118.3 (q, *J* = 3.7 Hz, CHCCF_3_), 64.0 (OCH_2_), 54.9 (CH), 39.1 (CH), 32.1 (CH_2_), 26.2 (CH_2_), 14.03 (CH_3_). HRMS-ESI [M+H] + *m*/*z* calcd for C_19_H_18_F_3_N_4_O_3_: 407.13255, found: 407.13179.

(4aR*,8aS*)-3-Acetyl-1-(*p*-tolyl)-4a,5,8,9-tetrahydro [1,2,4]triazolo[4,3-*a*]quinazoline-9(1H)-one (**4g**): 0.14 g (72%), white crystals, m.p. 165–169 °C. ^1^H NMR (500 MHz, CDCl_3_) δ 7.96–7.88 (m, 2H), 7.27 (d, *J* = 7.9 Hz, 2H), 5.90–5.80 (m, 1H), 5.66–5.61 (m, 1H), 4.35 (ddd, *J* = 13.3, 9.8, 5.0 Hz, 1H), 3.34 (dt, *J* = 16.2, 5.3 Hz, 1H), 2.76 (dt, *J* = 18.3, 4.9 Hz, 1H), 2.71 (s, 3H, CO*CH_3_*), 2.59 (ddd, *J* = 13.3, 11.4, 4.9 Hz, 1H), 2.39 (s, 3H, CH_3_, *p*-tolyl), 2.32 (ddtd, *J* = 15.9, 13.7, 4.7, 2.4 Hz, 1H), 2.22–2.13 (m, 1H). ^13^C NMR (126 MHz, CDCl_3_) δ 187.5 (C=O), 176.4 (C=O), 153.9 (C), 144.0 (C), 138.0 (C), 133.9 (C), 129.7 (CH), 127.0 (CH), 122.6 (CH), 121.7 (CH), 55.1 (CH), 39.3 (CH), 32.8 (CH_2_), 27.2 (CH), 26.47(CH_2_), 21.07(CH_3_). HRMS-ESI [M+H] + *m*/*z* calcd for C_18_H_19_N_4_O_2_: 323.15025, found: 323.14957.

(4aR*,8aS*)-9-Oxo-N-phenyl-1-(*p*-tolyl)-5-oxo-1,4a,5,8,8a,9-hexahydro [1,2,4]triazolo[4,3-*a*]quinazoline-3-carboxamide (**4h**): 0.17 g (71%), white crystals, m.p. 273–276 °C. ^1^H NMR (500 MHz, CDCl_3_) δ 8.79 (s, 1H, NH), 7.89–7.86 (m, 2H), 7.69–7.66 (m, 2H), 7.42 (dd, *J* = 10.8, 5.2 Hz, 2H), 7.26–7.21 (m, 3H), 5.84 (dd, *J* = 8.9, 3.8 Hz, 1H), 5.62 (dd, *J* = 9.0, 5.2 Hz, 1H), 4.36 (ddd, *J* = 13.4, 9.8, 5.0 Hz, 1H), 3.65–3.51 (m, 1H), 2.74 (dd, *J* = 13.7, 9.1 Hz, 1H), 2.61–2.45 (m, 1H), 2.38 (s, 3H, CH_3_, *p*-tolyl), 2.36–2.17 (m, 2H). ^13^C NMR (126 MHz, CDCl_3_) δ 176.7 (C=O), 153.7 (C=O), 153.6 ©, 141.3 (C), 138.0(C), 136.4 (C), 133.6 (C), 129.8 (CH), 129.4 (CH), 126.8 (CH), 125.8 (CH), 122.7 (CH), 121.6 (CH), 120.2 (CH), 55.1 (CH), 39.3 (CH), 32.4 (CH_2_), 26.5 (CH_2_), 21.1 (CH_3_). HRMS-ESI [M+H] + *m*/*z* calcd for C_23_H_22_N_5_O_2_: 400.17680, found: 400.17611.

(4aS*,8aS*)-Ethyl 9-oxo-1-phenyl-1,4a,5,8,8a,9-hexahydro-[1,2,4]triazolo[4,3-*a*]quinazoline-3-carboxylate (**5a**): 0.16 g (82%), white crystals, m.p. 222–225 °C. ^1^H NMR (500 MHz, CDCl_3_) δ 8.05 (d, *J* = 7.6 Hz, 2H), 7.46 (t, *J* = 8.0 Hz, 2H), 7.33 (t, *J* = 7.4 Hz, 1H), 5.93–5.82 (m, 1H), 5.67–5.59 (m, 1H), 4.59–4.44 (m, 2H), 4.37 (ddd, *J* = 13.6, 9.7, 5.2 Hz, 1H), 3.29 (dd, *J* = 12.2, 7.1 Hz, 1H), 2.83–2.72 (m, 1H), 2.62 (ddd, *J* = 13.4, 11.1, 5.0 Hz, 1H), 2.42–2.23 (m, 2H), 1.47 (t, *J* = 7.1 Hz, 3H). ^13^C NMR (126 MHz, CDCl_3_) δ 176.2 (C=O), 157.1 (C=O), 153.4 (C), 138.8 (C), 136.3(C), 129.13(CH), 127.78(CH), 127.32(CH), 122.05(CH), 121.84(CH), 63.70(CH_2_), 54.88(CH), 39.15(CH), 32.16(CH_2_), 26.3(CH_2_), 14.00(CH_3_). HRMS-ESI [M+H] + *m*/*z* calcd for C_18_H_19_N_4_O_3_: 339.14517, found: 339.14446.

(4aS*,8aS*)-Ethyl 9-oxo-1-(*p*-tolyl)-1,4a,5,8,8a,9-hexahydro-[1,2,4]triazolo[4,3-*a*]quinazoline-3-carboxylate (**5b**): 0.18 g (83%), white crystals, m.p. 200–202 °C. ^1^H NMR (500 MHz, CDCl_3_) δ 7.90 (d, *J* = 8.5 Hz, 2H), 7.24 (d, *J* = 8.3 Hz, 2H), 5.88 (dd, *J* = 9.3, 4.5 Hz, 1H), 5.63 (dd, *J* = 9.5, 5.8 Hz, 1H), 4.60–4.42 (m, 2H, CH_3_*CH_2_*), 4.35 (ddd, *J* = 13.6, 9.7, 5.2 Hz, 1H), 3.33–3.22 (m, 1H), 2.82–2.71 (m, 1H), 2.60 (ddd, *J* = 13.3, 11.2, 5.0 Hz, 1H), 2.37 (s, 3H), 2.35–2.24 (m, 2H), 1.47 (t, *J* = 7.1 Hz, 3H, *CH_3_*CH_2_). ^13^C NMR (126 MHz, CDCl_3_) δ 176.2 (C=O), 157.1 (C=O), 153.4 (C), 138.7(C), 137.9(C), 133.8 (C), 129.7(CH), 127.3 (CH), 122.1(CH), 121.8(CH), 63.6 (CH_2_), 54.9(CH), 39.2(CH), 32.2(CH_2_), 26.3 (CH_2_), 21.1 (CH_3_), 14.0 (CH_3_). HRMS-ESI [M+H] + *m*/*z* calcd for C_19_H_21_N_4_O_3_: 353.16082, found: 353.16005.

(4aS*,8aS*)-Ethyl 1-(4-nitrophenyl)-9-oxo-1,4a,5,8,8a,9-hexahydro-[1,2,4]triazolo[4,3-*a*]quinazoline-3-carboxylate (**5c**): 0.16 g (71%), white crystals, m.p. 236–238 °C. ^1^H NMR (500 MHz, CDCl_3_) δ 8.46 (d, *J* = 9.3 Hz, 2H), 8.31 (d, *J* = 9.3 Hz, 2H), 5.97–5.79 (m, 1H), 5.71–5.56 (m, 1H), 4.66–4.44 (m, 2H, CH_3_*CH_2_*), 4.39 (ddd, *J* = 13.7, 9.6, 5.1 Hz, 1H), 3.33–3.15 (m, 1H), 2.84–2.70 (m, 1H), 2.64 (ddd, *J* = 13.4, 11.1, 4.9 Hz, 1H), 2.44–2.21 (m, 2H), 1.49 (t, *J* = 7.1 Hz, 1H,* CH_3_*CH_2_). ^13^C NMR (126 MHz, CDCl_3_) δ 176.0 (C=O), 156.8 (C=O), 153.7 (C), 146.0 (C), 141.3(C), 139.6 (C), 127.2 (CH), 124.7 (CH), 121.9 (CH), 121.2(CH), 64.0 (CH_2_), 54.9 (CH), 39.1 (CH), 32.1(CH_2_), 26.1 (CH_2_), 14.0 (CH_3_). HRMS-ESI [M+H] + *m*/*z* calcd for C_18_H_18_N_5_O_5_: 384.13025, found: 384.12980.

(4aS*,4aS*)-Ethyl 1-(4-methoxyphenyl)-9-oxo-1,4a,5,8,8a,9-hexahydro-[1,2,4]triazolo[4,3-*a*]quinazoline-3-carboxylate (**5d**): 0.20 g (90%), white crystals, m.p. 201–204 °C. ^1^H NMR (500 MHz, CDCl_3_) δ 7.90 (d, *J* = 9.1 Hz, 2H), 6.95 (d, *J* = 9.1 Hz, 2H), 5.88 (dd, *J* = 7.7, 5.7 Hz, 1H), 5.63 (dd, *J* = 9.7, 5.7 Hz, 1H), 4.58–4.44 (m, 1H, CH_3_*CH_2_*), 4.36 (ddd, *J* = 13.9, 9.7, 5.1 Hz, 1H), 3.83 (s, 3H, O*CH_3_*), 3.35–3.22 (m, 1H), 2.83–2.71 (m, 1H), 2.60 (ddd, *J* = 13.3, 11.2, 4.9 Hz, 1H), 2.39–2.23 (m, 2H), 1.46 (t, *J* = 7.1 Hz, 3H, *CH_3_*CH_2_). ^13^C NMR (126 MHz, CDCl_3_) δ 176.17(C=O), 159.11(C=O), 157.12(C), 153.4(C), 138.6(C), 129.3(C), 127.3(CH), 123.7(CH), 122.1(CH), 114.3(CH), 63.6(CH_2_), 55.6(OCH_3_), 55.0(CH), 39.2(CH), 32.2(CH_2_), 26.4(CH_2_), 14.00(CH_3_). HRMS-ESI [M+H] + *m*/*z* calcd for C_19_H_21_N_4_O_4_: 369.15573, found: 369.15513.

(4aS*,8aS*)-Ethyl 1-(4-chlorophenyl)-9-oxo-1,4a,5,8,8a,9-hexahydro-[1,2,4]triazolo[4,3-*a*]quinazoline-3-carboxylate (**5e**): 0.18 g (79%), white crystals, m.p. 231–233 °C. ^1^H NMR (500 MHz, CDCl_3_) δ 8.08 (d, *J* = 12.0 Hz, 2H), 7.42 (d, *J* = 9.0 Hz, 2H), 5.88 (dd, *J* = 8.8, 4.9 Hz, 1H), 5.63 (dd, *J* = 7.5, 6.0 Hz, 1H), 4.62–4.44 (m, 2H, CH_3_*CH_2_*), 4.41–4.29 (m, 1H), 3.33–3.19 (m, 1H), 2.82–2.71 (m, 1H), 2.61 (ddd, *J* = 13.4, 11.2, 4.9 Hz, 1H), 2.39–2.22 (m, 2H), 1.47 (t, *J* = 7.1 Hz, 3H, *CH_3_*CH_2_). ^13^C NMR (126 MHz, CDCl_3_) δ 176.1(C=O), 157.0(C=O), 153.4(C), 138.9(C), 134.9(C), 133.3(C), 129.2(CH), 127.3(CH), 122.7(CH), 122.0(CH), 63.8(CH_2_), 54.9(CH), 39.1(CH), 32.1 (CH_2_), 26.3(CH_2_), 14.0(CH_3_). HRMS-ESI [M+H] + *m*/*z* calcd for C_18_H_18_ClN_4_O_3_: 373.10619, found: 373.10560.

(4aS*,8aS*)-Ethyl 9-oxo-1-(3-(trifluoromethyl)phenyl)-1,4a,5,8,8a,9-hexahydro-[1,2,4]triazolo[4,3-*a*]quinazoline-3-carboxylate: (**5f**): 0.18 g (76%), white crystals, m.p. 162–164 °C. ^1^H NMR (500 MHz, CDCl_3_) δ 8.55–8.46 (m, 1H), 8.24 (s, 1H), 7.63–7.55 (m, 2H), 5.89 (dd, *J* = 8.4, 4.7 Hz, 1H), 5.64 (dd, *J* = 8.6, 4.9 Hz, 1H), 4.61–4.46 (m, 2H, CH_3_CH_2_), 4.38 (ddd, *J* = 13.6, 9.6, 5.2 Hz, 1H), 3.36–3.16 (m, 1H), 2.77 (dt, *J* = 8.7, 4.6 Hz, 1H), 2.62 (ddd, *J* = 13.4, 11.1, 5.0 Hz, 1H), 2.45–2.18 (m, 2H), 1.49 (t, *J* = 7.1 Hz, 3H, CH_3_CH_2_). ^13^C NMR (126 MHz, CDCl_3_) δ 176.1 (C=O), 156.9(C=O), 153.6(C), 139.2(C), 136.8(C), 131.7 (q, *J* = 34 Hz, C-CF_3_) 129.9 (CH), 127.3(CH), 124.9(CH), 124.2 (q, *J* = 3.6 Hz, CHCCF_3_), 123.5 (q, *J* = 270 Hz, CF_3_) 122.0(CH), 118.2 (q, *J* = 3.7 Hz, CHCCF_3_), 63.9 (OCH_2_), 54.9 (CH), 39.2 (CH), 32.1 (CH_2_), 26.2 (CH_2_), 14.0 (CH_3_). HRMS-ESI [M+H] + *m*/*z* calcd for C_19_H_18_F_3_N_4_O_3_: 407.13255, found: 407.13150.

(4aS*,8aS*)-3-Acetyl-1-(*p*-tolyl)-4a,5,8,9-tetrahydro-[1,2,4]triazolo[4,3-*a*]quinazolin-9(1H)-one (**5g**): 0.14 g (71%), white crystals, m.p. 162–165 °C. ^1^H NMR (500 MHz, CDCl_3_) δ 7.93 (d, *J* = 8.6 Hz, 2H), 7.26 (d, *J* = 8.1 Hz, 2H), 5.90–5.82 (m, 1H), 5.67–5.60 (m, 1H), 4.34 (ddd, *J* = 13.4, 9.8, 5.1 Hz, 1H), 3.39–3.28 (m, 1H), 2.76 (dt, *J* = 18.5, 5.0 Hz, 1H), 2.71 (s, 3H, CO*CH_3_*), 2.63–2.54 (m, 1H), 2.39 (s, 3H, CH_3_, *p*-tolyl)), 2.32 (ddtd, *J* = 15.9, 13.6, 4.6, 2.4 Hz, 1H), 2.17 (dddt, *J* = 12.2, 7.0, 4.6, 2.4 Hz, 1H). ^13^C NMR (126 MHz, CDCl_3_) δ 187.5 (C=O), 176.5 (C=O), 153.9 (C), 144.0 (C), 138.0 (C), 133.9 (C), 129.7 (CH), 127.0 (CH), 122.6 (CH), 121.7 (CH), 55.1 (CH), 39.3 (CH), 32.8 (CH_2_), 27.2 (CH), 26.47(CH_2_), 21.1(CH_3_). HRMS-ESI [M+H] + *m*/*z* calcd for C_18_H_19_N_4_O_2_: 323.15025, found: 323.14953.

(4aS*,8aS*)-9-Oxo-N-phenyl-1-(*p*-tolyl)-1,4a,5,8,8a,9-hexahydro-[1,2,4]triazolo[4,3-*a*]quinazoline-3-carboxamide (**5h**): 0.17 g (71%), white crystals, m.p. 282–284 °C. ^1^H NMR (500 MHz, CDCl_3_) δ 8.81 (s, 1H), 7.87 (d, *J* = 8.4 Hz, 2H), 7.67 (d, *J* = 7.9 Hz, 2H), 7.41 (t, *J* = 7.9 Hz, 2H), 7.24 (d, *J* = 8.2 Hz, 3H), 5.86–5.78 (m, 1H), 5.66–5.58 (m, 1H), 4.41–4.27 (m, 1H), 3.57 (d, *J* = 15.4 Hz, 1H), 2.74 (d, *J* = 17.9 Hz, 1H), 2.59–2.45 (m, 1H), 2.37 (s, 3H,CH_3_, *p*-tolyl), 2.35–2.13 (m, 2H). ^13^C NMR (126 MHz, CDCl_3_) δ 176.56(C=O), 153.67(C=O), 141.28(C), 137.92(C), 136.47(C), 133.71(C), 129.73(CH), 129.33(CH), 126.79(CH), 125.75(CH), 122.65(CH), 121.58(CH), 120.26(CH), 55.14(CH), 39.29(CH), 32.37(CH_2_), 26.46 CH_2_), 21.06 CH_3_). HRMS-ESI [M+H] + *m*/*z* calcd for C_23_H_22_N_5_O_2_: 400.17680, found: 400.17591.

### 3.3. X-ray Structure Determinations

After immersing the crystal of **5a** mounted on a loop into cryo-oil at a temperature of 120 K, XRD data were collected (Rigaku Oxford Diffraction Supernova device, Cu Kα radiation). Cell refinement and data reduction were achieved using the *CrysAlisPro* software package ((CrysAlisPro 1.171.40.53), CrysAlisPro = CrysAlisPro package, SHELXL 2017/1, SHELXT 2018/2, SHELXLE rev. 1320) [35]. The intensities were corrected before structure determination (Gaussian absorption correction (*CrysAlisPro* [40]), intrinsic phasing (*SHELXT* [40]) method). Additional structural refinements were also carried out (*SHELXL* [41] software with the *SHELXLE* [42] graphical user interface). The crystal contained two independent molecules in the asymmetric unit. Hydrogen atoms were positioned geometrically on their parent atoms, with C–H = 0.95–1.00 Å and U_iso_ = 1.2–1.5·U_eq_ (parent atom) (Appendix A). Other important structural details can be found in Table S1 (Supplementary Materials).

### 3.4. Determination of Antiproliferative Properties of the Prepared Compounds

The antiproliferative actions of the selected compounds (4a–e, 4g,h, and 5a–h) were investigated through the MTT (3-(4,5-dimethylthiazol-2-yl)-2,5-diphenyltetrazolium bromide) assay using a set of adherent cell lines isolated from human (SiHa), ovarian (A2780), and breast (MCF-7 and MDA-MB-231) cancers [43]. All cell lines were obtained from the European Collection of Cell Cultures (ECCAC, Salisbury, UK), except for SiHa, which was purchased from the American Tissue Culture Collection (Manassas, VA, USA). The cells were grown in minimal essential medium supplemented with fetal bovine serum (10%), non-essential amino acids (1%), and a penicillin–streptomycin–amphotericin B mixture (1%). All cell culture components were purchased from Lonza Group Ltd. (Basel, Switzerland). Malignant cells were plated into 96-well plates at the density of 5000/well, and the next day, the test substance was added in 10 μM or 30 μM final concentrations. After 72 h of incubation, MTT solution (5 mg/mL, 20 μL) was added to each well and incubated for 4 h. Finally, the medium was discarded, and the formazan was solubilized in 100 μL DMSO during 60 min of shaking at 37 °C. The absorbance was determined at 545 nm using a microplate reader (SpectoStarNano, BMG Labtech, Ortenberg, Germany). Two independent experiments were carried out with five wells for each condition, and cisplatin (Ebewe GmbH, Unterach, Austria) was included as a reference agent.

## 4. Conclusions

New angular 1,2,4-triazolo[4,3-*a*]quinazolin-9-ones with various functionalities were prepared in a simple and regioselective manner by reacting variously functionalized hydrazonoyl chlorides and cyclohexene-based 2-thioxopyrimidin-4-ones **1** and **2** that favored the formation of angular products rather than linear ones. Both *cis* and *trans* isomers of 2-thioxopyrimidin-4-ones led to the formation of angular products. Among all the prepared compounds, only the *cis* isomer of triazole **4c** with a *p*-nitrophenyl substituent of the ring revealed significant growth inhibition against all the used cancer cells.

## Figures and Tables

**Table 1:** Antiproliferative action of the tested compounds.

Comp.	Conc.	Inhibition of Cancer Growth (%) ± SEM			
		MDA-MB-231	MCF-7	SiHa	A2780
**4c**	10 μM	33.47 ± 0.90	41.80 ± 1.27	31.22 ± 0.57	24.37 ± 1.72
	30 μM	34.63 ± 2.55	46.85 ± 1.12	36.93 ± 0.48	30.55 ± 2.70
**4d**	10 μM	14.65 ± 3.33	– *	–	–
	30 μM	14.75 ± 2.66	–	–	–
**4e**	10 μM	–	–	–	–
	30 μM	12.02 ± 4.48	–	–	–
**4g**	10 μM	–	–	17.29 ± 2.24	–
	30 μM	–	–	19.37 ± 1.29	–
**4h**	10 μM	–	–	–	–
	30 μM	–	12.87 ± 1.16	–	–
**5b**	10 μM	–	–	17.32 ± 1.90	–
	30 μM	13.52 ± 1.87	27.04 ± 2.39	27.34 ± 3.28	19.65 ± 2.09
**5c**	10 μM	–	27.49 ± 2.70	16.58 ± 2.27	10.86 ± 2.32
	30 μM	–	35.28 ± 2.09	21.43 ± 2.95	24.62 ± 1.73
**5d**	10 μM	–	–	–	–
	30 μM	17.97 ± 2.44	25.51 ± 2.52	23.19 ± 2.39	17.34 ± 2.72
**5e**	10 μM	–	21.01 ± 2.77	21.04 ± 2.90	–
	30 μM	–	23.38 ± 2.86	22.36 ± 2.58	14.53 ± 2.93
**5f**	10 μM	–	12.75 ± 2.79	–	–
	30 μM	–	20.59 ± 2.79	–	–
**5g**	10 μM	–	21.18 ± 2.55	10.08 ± 1.07	–
	30 μM	–	24.48 ± 2.82	10.02 ± 2.45	12.61 ± 2.53
**5h**	10 μM	–	–	–	–
	30 μM	17.03 ± 2.36	39.01 ± 2.20	32.78 ± 2.89	31.90 ± 1.59
Cispl.	10 μM	42.72 ± 2.68	54.06 ± 1.17	88.64 ± 0.5	83.57 ± 1.21
	30 μM	88.43 ± 0.42	95.45 ± 0.28	90.18 ± 1.78	95.02 ± 0.28

* Cell proliferation inhibition values less than 10% were regarded as negligible and are not shown numerically.

## Data Availability

Samples of the compounds are not available from the authors.

## Supplementary Materials

The following are available online at https://www.mdpi.com/article/10.3390/molecules28093718/s1, NMR spectra of all synthesized compounds and crystallographic details of **5a** and Table S1: Crystal Data.

## Sample Availability

Samples of the compounds are available from the authors.

## Appendix A

**5a**: C_18_H_18_N_4_O_3_; a colourless needle of dimensions 0.02 × 0.06 × 0.132 mm^3^ gave a triclinic space group P 1, a = 9.5332(3) Å, b = 10.4081 (3) Å, c = 16.8864(5) Å, α = 89.227(2)°, β = 81.130(2)°, γ = 74.199(3)°, λ = 1.54184 Å, V = 1.592.20(3) Å, T = 120(2) K, *ρ*_calc_ = 1.412 Mg/m^3^, θ range: 2.650 to 76.986°, No. reflections: 42,738, no. unique reflections: 6682, completeness to θ_67.684°_ = 100%, GOOF = 1.029, R_int_ = 0.0503, R1 = 0.0377, wR2 = 0.0932 with *R1* = Σ||*F*_o_| − |*F*_c_||/Σ|*F*_o_|. *wR*_2_ = {Σ[*w*(*F*_o_^2^ − *F*_c_^2^)^2^]/Σ[*w*(*F*_o_^2^)^2^]}^1/2^.
