# Supplementary material for: Angular Regioselective Synthesis of Varied Functionalized Hexahydro-1,2,4-triazolo[4,3-a]quinazolin-9-ones and Their Antiproliferative Action
Source: Molecules. 2023 Apr 25;28(9):3718. doi: 10.3390/molecules28093718 (PMC10180492; doi:10.3390/molecules28093718)

Angular Regioselective Synthesis of Varied Functionalized Hexahydro[1,2,4]triazolo[4,3-*a*]quinazolin-9-ones and Their Antiproliferative Action

Table of Contents:

Copies of NMR spectra of compounds 4 and 5:

(4a*S**,8a*R**)-Ethyl 9-oxo-1-phenyl-1,4a,5,8,8a,9-hexahydro[1,2,4]triazolo[4,3-*a*]quinazoline-3-carboxylate (**4a**) **S3**

(4a*S**,8a*R**)-Ethyl 9-oxo-1-(*p*-tolyl)-1,4a,5,8,8a,9-hexahydro[1,2,4]triazolo[4,3-*a*]quinazoline-3-carboxylate (**4b**) **S6**

(4a*S**,8a*R**)-Ethyl 9-oxo-1-(4-nitrophenyl)-1,4a,5,8,8a,9-hexahydro-[1,2,4]triazolo[4,3-*a*]quinazoline-3-carboxylate (**4c**) **S9**

(4a*S**,8a*R**)-Ethyl 9-oxo-1-(4-methoxyphenyl)-1,4a,5,8,8a,9-hexahydro[1,2,4]triazolo-[4,3-*a*]quinazoline-3-carboxylate (**4d**) **S10**

(4a*S**,8a*R**)-Ethyl 9-oxo-1-(4-chlorophenyl)-1,4a,5,8,8a,9-hexahydro[1,2,4]triazolo[4,3-*a*]quinazoline-3-carboxylate (**4e**) **S13**

(4a*S**,8a*R**)-Ethyl 9-oxo-1-(3-(trifluoromethyl)phenyl)-1,4a,5,8,8a,9-hexahydro[1,2,4]triazolo-[4,3-*a*]quinazoline-3-carboxylate (**4f**) **S14**

(4a*S**,8a*R**)-3-Acetyl-1-(*p*-tolyl)-4a,5,8,9-tetrahydro[1,2,4]triazolo[4,3-*a*]quinazoline-9(1*H*)-one (**4g**) .. **S15**

(4a*S**,8a*R**)-9-Oxo-*N*-Phenyl-1-(*p*-tolyl)-1,4a,5,8,8a,9-hexahydro[1,2,4]triazolo[4,3-*a*]quinazoline-3-carboxamide (**4h**) **S16**

(4a*S**,8a*S**)-Ethyl 9-oxo-1-phenyl-1,4a,5,8,8a,9-hexahydro-[1,2,4]triazolo[4,3-a]quinazoline-3-carboxylate (**5a**) **S17**

(4a*S**,8a*S**)-Ethyl 9-oxo-1-(*p*-tolyl)-1,4a,5,8,8a,9-hexahydro-[1,2,4]triazolo[4,3-*a*]quinazoline-3-carboxylate (**5b**) **S21**

(4a*S**,8a*S**)-Ethyl 1-(4-nitrophenyl)-9-oxo-1,4a,5,8,8a,9-hexahydro-[1,2,4]triazolo[4,3-*a*]quinazoline-3-carboxylate (**5c**) **S22**

(4a*S**,8a*S**)-Ethyl 1-(4-methoxyphenyl)-9-oxo-1,4a,5,8,8a,9-hexahydro-[1,2,4]triazolo-[4,3-*a*]quinazoline-3-carboxylate (**5d**) **S23**

(4a*S**,8a*S**)-Ethyl 1-(4-chlorophenyl)-9-oxo-1,4a,5,8,8a,9-hexahydro-[1,2,4]triazolo[4,3-*a*]quinazoline-3-carboxylate (**5e**) **S24**

(4a*S**,8a*S**)-Ethyl 9-oxo-1-(3-(trifluoromethyl)phenyl)- 1,4a,5,8,8a,9-hexahydro-[1,2,4]triazolo-[4,3-*a*]quinazoline-3-carboxylate (**5f**) **S25**

(4a*S**,8a*S**)-3-Acetyl-1-(*p*-tolyl)-4a,5,8,9-tetrahydro-[1,2,4]triazolo[4,3-*a*]quinazolin-9(1*H*)-one (**5g**) ... **S26**

(4a*S**,8a*S**)-9-Oxo-*N*-phenyl-1-(*p*-tolyl)-1,4a,5,8,8a,9-hexahydro-[1,2,4]triazolo[4,3-*a*]quinazoline-3-carboxamide (**5h**) ... **S27**

Crystallographic details of compound 5a ... **S28**

Copies of HRMS-ESI Spectra of compounds 4a-h and 5a-h ... **S29**

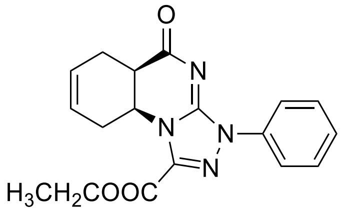

(4a*S**,8a*R**)-Ethyl 9-oxo-1-phenyl-1,4a,5,8,8a,9-hexahydro[1,2,4]triazolo[4,3-*a*]quinazoline-3-carboxylate (**4a**)

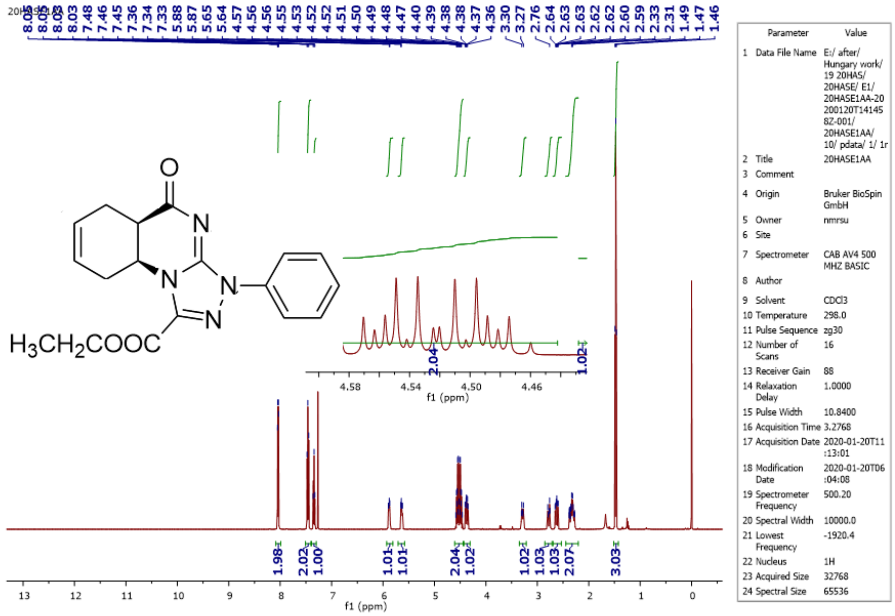

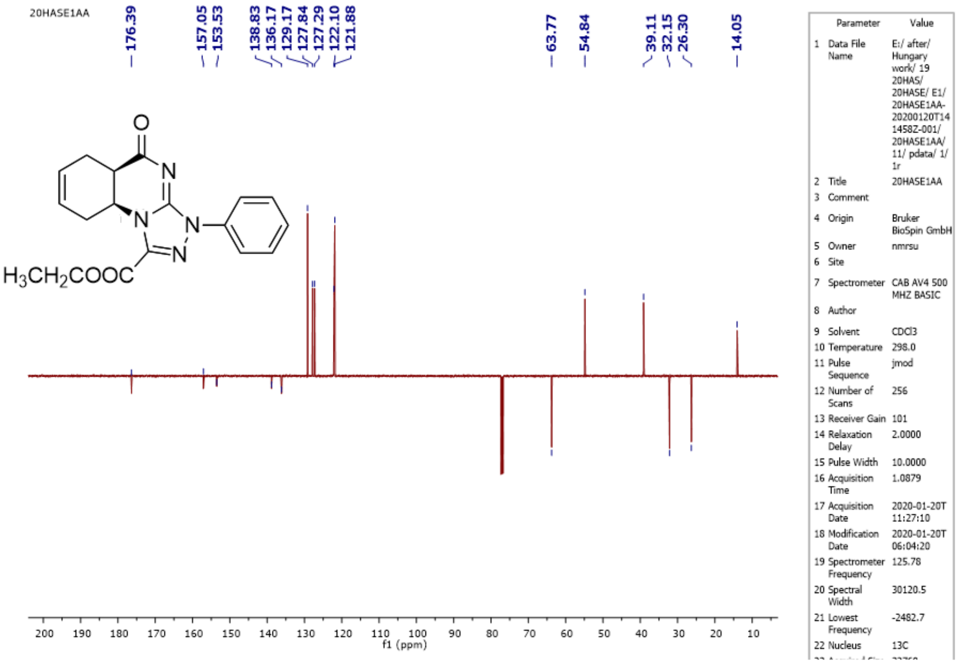

COSY Experiments

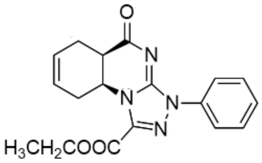

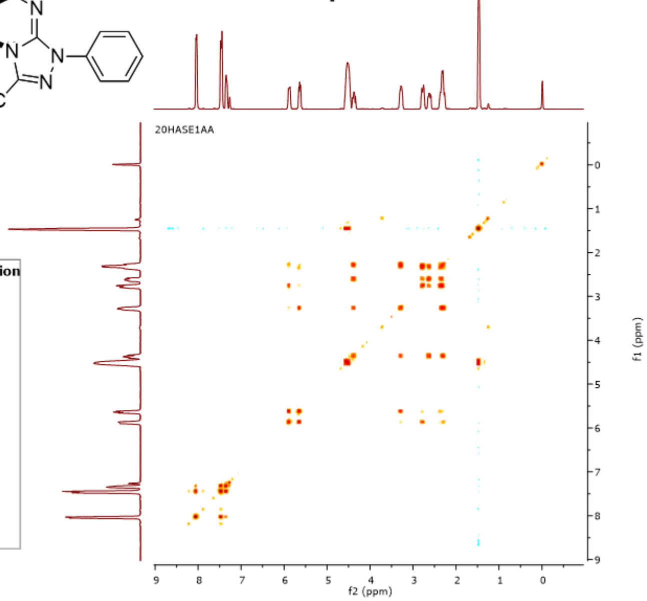

H-H Correlation

f1 (ppm)	f2 (ppm)
4.36	2.29
4.38	2.63
2.62	2.34
4.54	1.48
7.45	7.33
5.88	2.81
4.28	3.26
5.64	3.29
2.79	2.33
5.90	5.63
3.27	2.30
8.04	7.44

	Parameter	Value (f2, f1)
1	Data File Name	E:/ after/ Hungary work/ 19 20HAS/ 20HASE/ E1/ 20HASE1AA-20 200120T14145 8Z-001/ 20HASE1AA/ 12/ pdata/ 1/ 2rr
2	Title	20HASE1AA
3	Comment	
4	Origin	Bruker BioSpin GmbH
5	Owner	nmrsu
6	Site	
7	Spectrometer	CAB AV4 500 MHZ BASIC
8	Author	
9	Solvent	CDCl3
10	Temperature	298.0
11	Pulse Sequence	cosygpppqf
12	Number of Scans	1
13	Receiver Gain	101
14	Relaxation Delay	1.9508
15	Pulse Width	10.8400
16	Acquisition Time	0.2048
17	Acquisition Date	2020-01-20T11:33:00
18	Modification Date	2020-01-20T06:04:30
19	Spectrometer Frequency	(500.20, 500.20)
20	Spectral Width	(5000.0, 5000.0)
21	Lowest Frequency	(-483.9, -483.9)
22	Nucleus	(1H, 1H)
23	Acquired Size	(1024, 128)
24	Spectral Size	(1024, 1024)

TOCSY Experiments

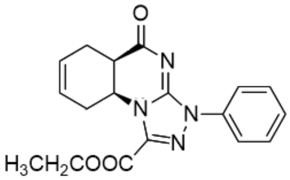

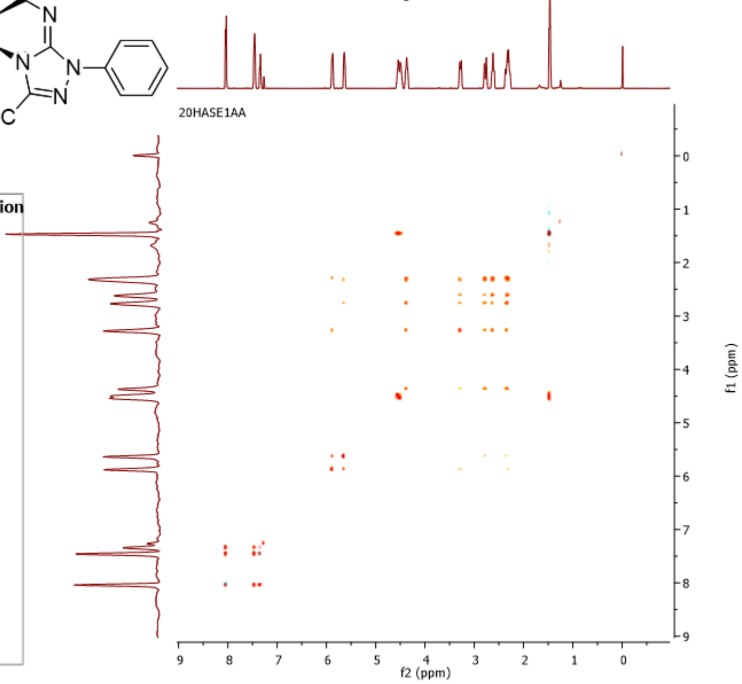

H-H Correction

f1 (ppm)	f2 (ppm)
5.88	2.75
4.52	1.46
5.64	3.28
4.37	2.77
4.37	2.33
8.04	7.35
4.37	2.62
5.64	2.62
4.52	1.48
4.37	3.28
5.64	2.77
5.88	2.79
8.04	7.46
5.88	2.30
5.64	2.34
5.88	3.28
5.88	2.62
5.88	5.64

	Parameter	Value (f2, f1)
1	Data File Name	E:/ after/ Hungary work/ 19 20HAS/ 20HASE/ E1/ 20HASE1AA-20 200120T14145 8Z-001/ 20HASE1AA/ 14/ pdata/ 1/ 2rr
2	Title	20HASE1AA
3	Comment	
4	Origin	Bruker BioSpin GmbH
5	Owner	nmrsu
6	Site	
7	Spectrometer	CAB AV4 500 MHZ BASIC
8	Author	
9	Solvent	CDCl3
10	Temperature	298.0
11	Pulse Sequence	mlevphpp
12	Number of Scans	8
13	Receiver Gain	101
14	Relaxation Delay	2.0000
15	Pulse Width	10.8400
16	Acquisition Time	0.2048
17	Acquisition Date	2020-01-20T13:39:13
18	Modification Date	2020-01-20T06:04:54
19	Spectrometer Frequency	(500.20, 500.20)
20	Spectral Width	(5000.0, 5000.0)
21	Lowest Frequency	(-483.9, -483.9)
22	Nucleus	(1H, 1H)
23	Acquired Size	(1024, 256)
24	Spectral Size	(1024, 1024)

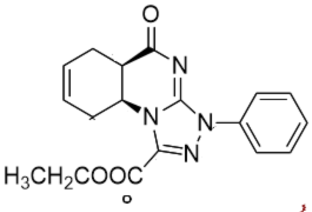

HSQC Experiments

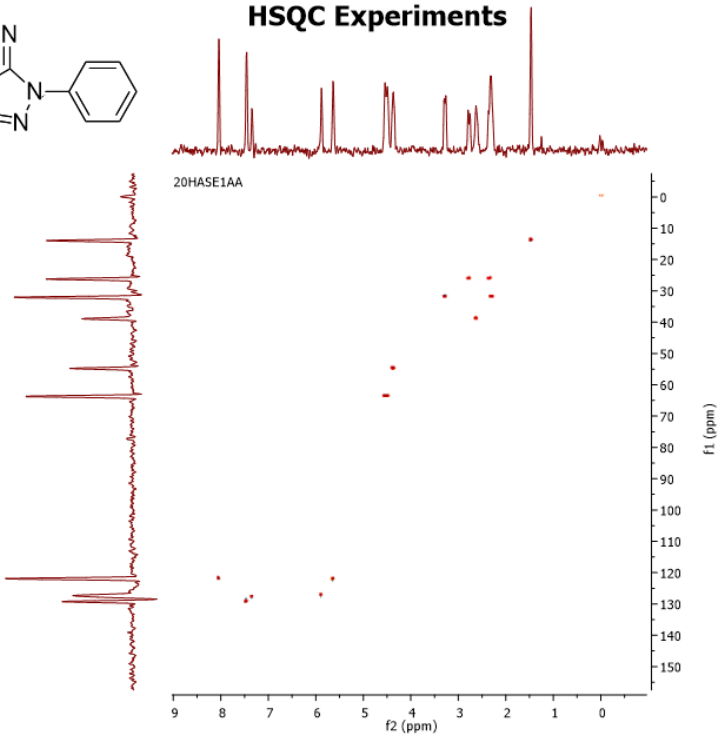

C-H Correction	
f1 (ppm)	f2 (ppm)
129.23	7.46
127.77	7.34
127.21	5.88
122.05	5.64
121.85	8.04
63.71	4.54
26.29	2.79
32.06	2.30
63.70	4.49
26.17	2.76
32.11	3.26
32.02	3.29
38.93	2.62
54.77	4.37
26.18	2.34
13.95	1.47

	Parameter	Value (f2, f1)
1	Data File Name	E:/ after/ Hungary work/ 19 20HAS/ 20HASE/ E1/ 20HASE1AA-20 200120T14145 8Z-001/ 20HASE1AA/ 15/ pdata/ 1/ 2rr
2	Title	20HASE1AA
3	Comment	
4	Origin	Bruker BioSpin GmbH
5	Owner	nmrsu
6	Site	
7	Spectrometer	CAB AV4 500 MHZ BASIC
8	Author	
9	Solvent	CDCl3
10	Temperature	298.0
11	Pulse Sequence	hsqcetgp
12	Number of Scans	2
13	Receiver Gain	101
14	Relaxation Delay	1.5000
15	Pulse Width	10.8400
16	Acquisition Time	0.1024
17	Acquisition Date	2020-01-20T13 :54:06
18	Modification Date	2020-01-20T06 :04:58
19	Spectrometer Frequency	(500.20, 125.78)
20	Spectral Width	(5000.0, 20752.3)
21	Lowest Frequency	(-483.9, -943.0)
22	Nucleus	(1H, 13C)
23	Acquired Size	(512, 256)
24	Spectral Size	(1024, 1024)

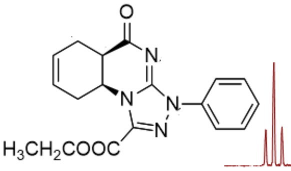

HMBC Experiments

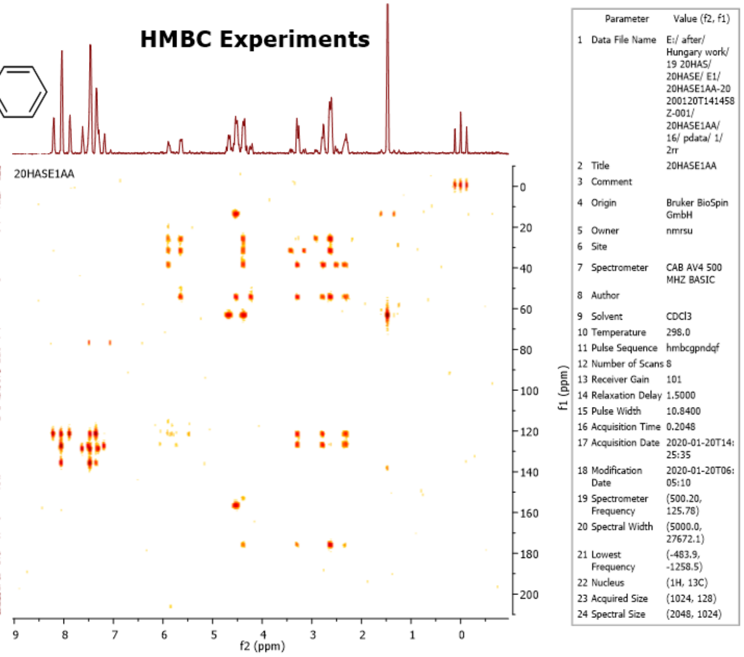

C-H[9a] Correlation	
f1 (ppm)	f2 (ppm)
174.21	4.38
156.13	4.38
63.54	4.38
38.67	4.38
31.93	4.38
26.13	4.38

	Parameter	Value (f2, f1)
1	Data File Name	E:/ after/ Hungary work/ 19 20HAS/ 20HASE/ E1/ 20HASE1AA-20 200120T141458 Z-001/ 20HASE1AA/ 16/ pdata/ 1/ 2rr
2	Title	20HASE1AA
3	Comment	
4	Origin	Bruker BioSpin GmbH
5	Owner	nmrsu
6	Site	
7	Spectrometer	CAB AV4 500 MHZ BASIC
8	Author	
9	Solvent	CDCl3
10	Temperature	298.0
11	Pulse Sequence	hmbcgpndqf
12	Number of Scans	8
13	Receiver Gain	101
14	Relaxation Delay	1.5000
15	Pulse Width	10.8400
16	Acquisition Time	0.2048
17	Acquisition Date	2020-01-20T14: 25:35
18	Modification Date	2020-01-20T06: 05:10
19	Spectrometer Frequency	(500.20, 125.78)
20	Spectral Width	(5000.0, 27672.1)
21	Lowest Frequency	(-483.9, -1258.5)
22	Nucleus	(1H, 13C)
23	Acquired Size	(1024, 128)
24	Spectral Size	(2048, 1024)

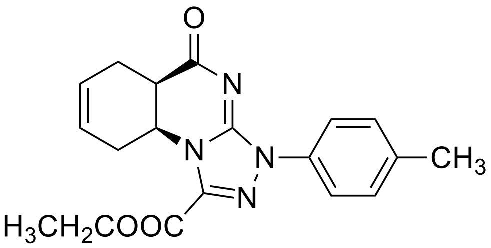

(4a*S**,8a*R**)-Ethyl 9-oxo-1-(*p*-tolyl)-1,4a,5,8,8a,9-hexahydro[1,2,4]triazolo[4,3-*a*]quinazoline-3-carboxylate (**4b**)

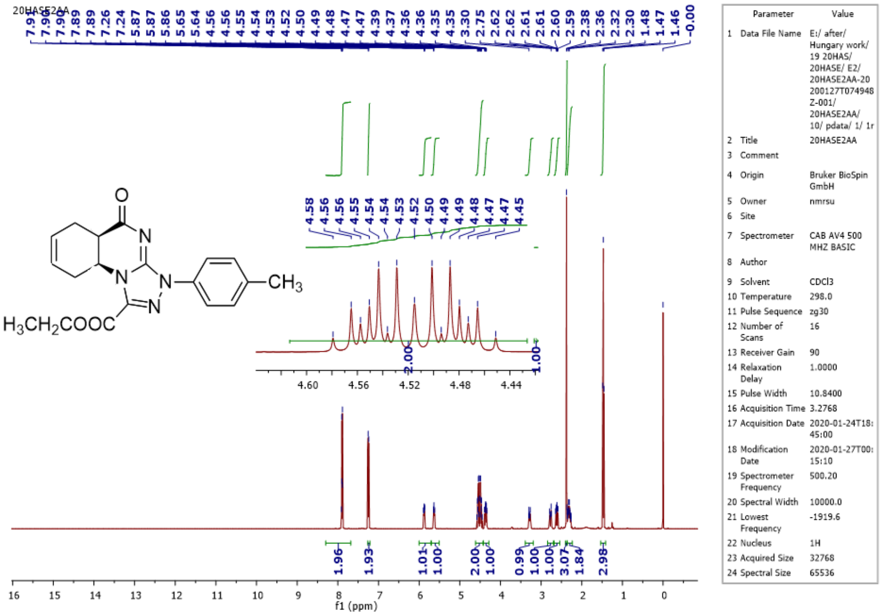

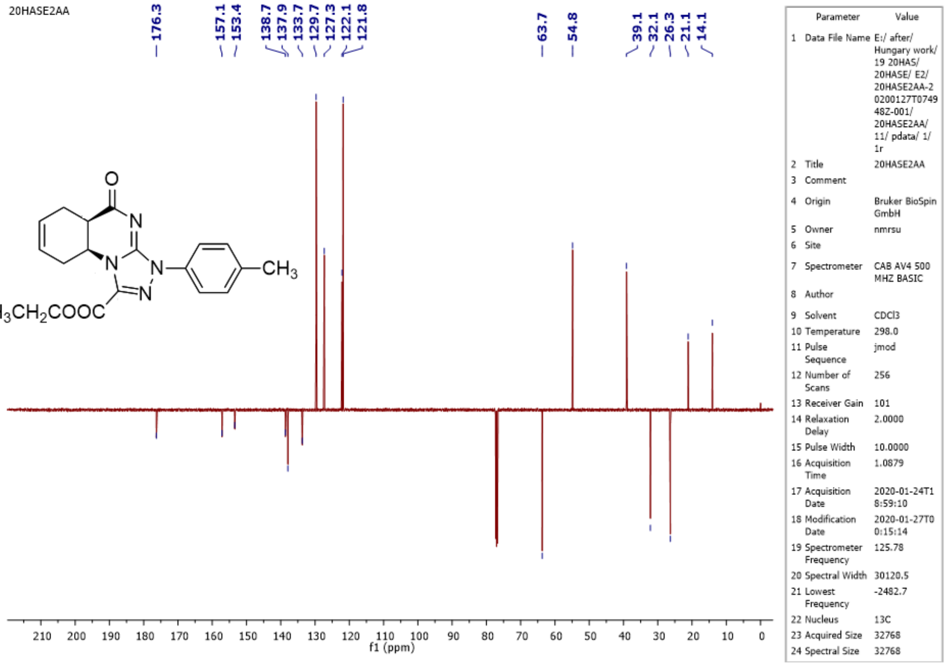

COSY Experiments

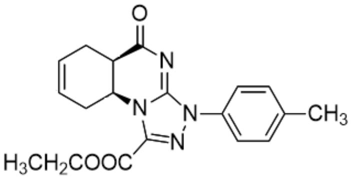

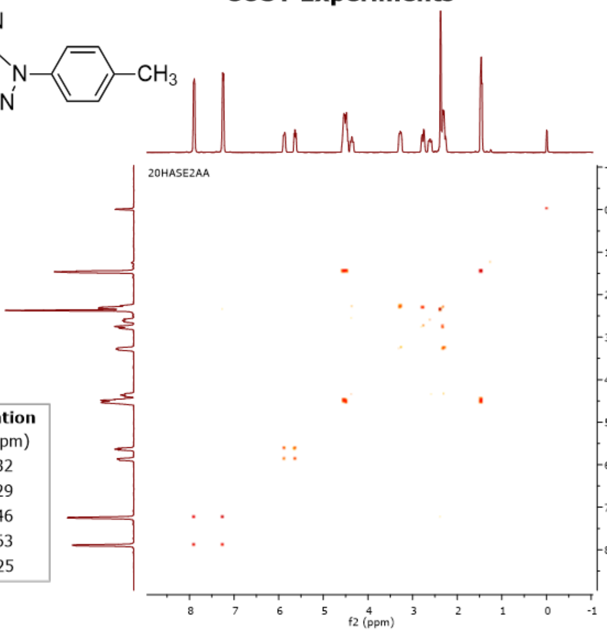

H-H Correlation	
f1 (ppm)	f2 (ppm)
2.78	2.32
3.28	2.29
4.51	1.46
5.88	5.63
7.89	7.25

	Parameter	Value (f2, f1)
1	Data File Name	E:/ after/ Hungary work/ 19 20HAS/ 20HASE/ E2/ 20HASE2AA-20 200127T07494 8Z-001/ 20HASE2AA/ 12/ pdata/ 1/ 2rr
2	Title	20HASE2AA
3	Comment	
4	Origin	Bruker BioSpin GmbH
5	Owner	nmrsu
6	Site	
7	Spectrometer	CAB AV4 500 MHZ BASIC
8	Author	
9	Solvent	CDCl3
10	Temperature	298.0
11	Pulse Sequence	cosygpppqf
12	Number of Scans	1
13	Receiver Gain	101
14	Relaxation Delay	1.9488
15	Pulse Width	10.8400
16	Acquisition Time	0.2048
17	Acquisition Date	2020-01-24T19:05:00
18	Modification Date	2020-01-27T00:15:20
19	Spectrometer Frequency	(500.20, 500.20)
20	Spectral Width	(5000.0, 5000.0)
21	Lowest Frequency	(-520.2, -520.2)
22	Nucleus	(1H, 1H)
23	Acquired Size	(1024, 128)
24	Spectral Size	(1024, 1024)

TOCSY Experiments

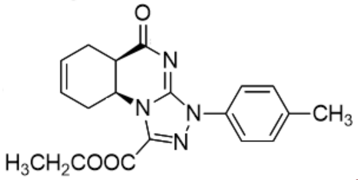

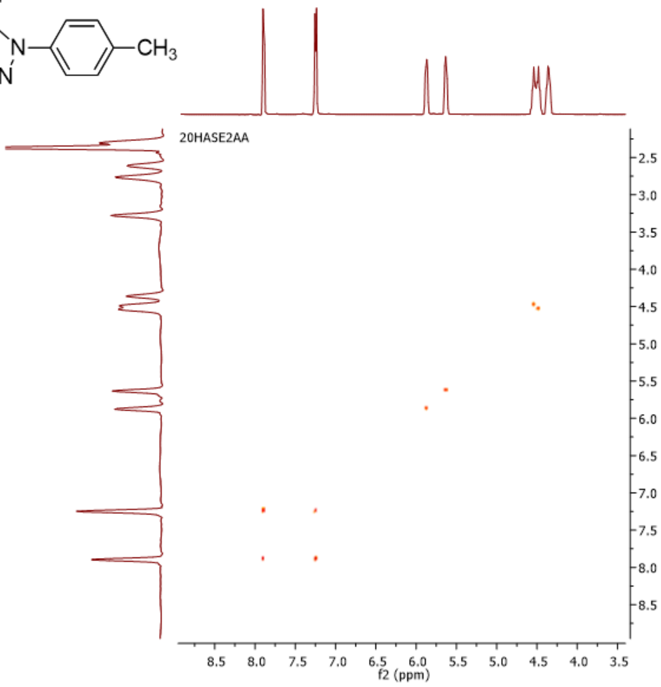

H-H Correlation	
f1 (ppm)	f2 (ppm)
4.36	3.27
4.36	2.76
4.36	2.60
4.36	2.32
4.51	1.46
4.53	1.46
5.63	4.36
5.63	3.28
5.63	2.32
5.63	2.60
5.63	2.76
5.88	5.63
5.88	4.36
5.88	2.29
5.88	2.61
5.88	2.77
5.88	3.27
7.89	7.25

	Parameter	Value (f2, f1)
1	Data File Name	E:/ after/ Hungary work/ 19 20HAS/ 20HASE/ E2/ 20HASE2AA-20 200127T07494 8Z-001/ 20HASE2AA/ 14/ pdata/ 1/ 2rr
2	Title	20HASE2AA
3	Comment	
4	Origin	Bruker BioSpin GmbH
5	Owner	nmrsu
6	Site	
7	Spectrometer	CAB AV4 500 MHZ BASIC
8	Author	
9	Solvent	CDCl3
10	Temperature	298.0
11	Pulse Sequence	mlevphpp
12	Number of Scans	8
13	Receiver Gain	101
14	Relaxation Delay	1.9980
15	Pulse Width	10.8400
16	Acquisition Time	0.2048
17	Acquisition Date	2020-01-24T21:11:06
18	Modification Date	2020-01-27T00:15:32
19	Spectrometer Frequency	(500.20, 500.20)
20	Spectral Width	(5000.0, 5000.0)
21	Lowest Frequency	(-520.2, -520.2)
22	Nucleus	(1H, 1H)
23	Acquired Size	(1024, 256)
24	Spectral Size	(1024, 1024)

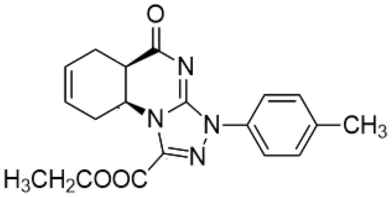

HSQC Experiments

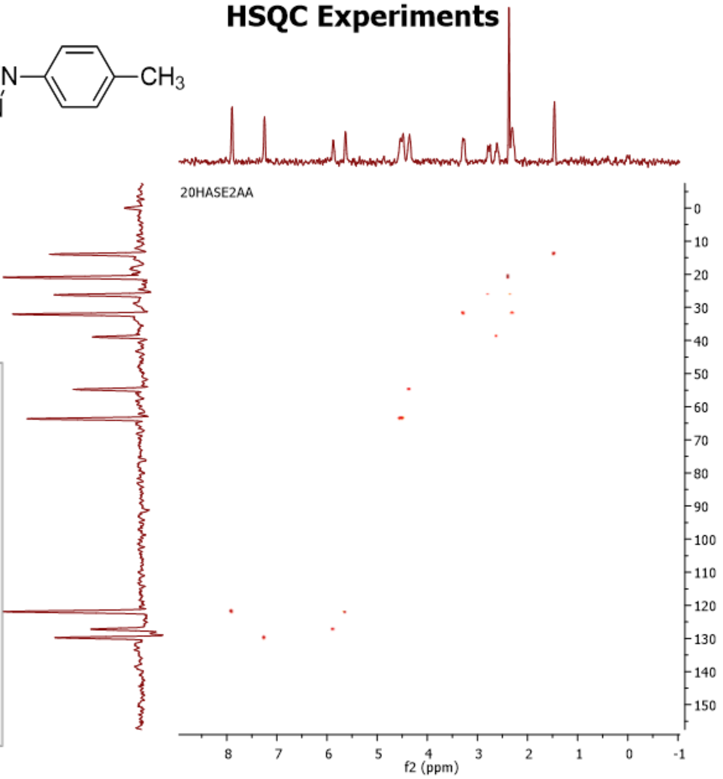

C-H Correlation	
f1 (ppm)	f2 (ppm)
13.95	1.46
20.89	2.37
26.21	2.77
26.21	2.33
32.02	2.29
32.03	3.29
38.95	2.61
54.76	4.35
63.63	4.48
121.85	7.89
122.01	5.63
127.18	5.87
129.76	7.25

	Parameter	Value (f2, f1)
1	Data File Name	E:/ after/ Hungary work/ 19 20HAS/ 20HASE/ E2/ 20HASE2AA-20 200127T074948 Z-001/ 20HASE2AA/ 15/ pdata/ 1/ 2rr
2	Title	20HASE2AA
3	Comment	
4	Origin	Bruker BioSpin GmbH
5	Owner	nmrsu
6	Site	
7	Spectrometer	CAB AV4 500 MHZ BASIC
8	Author	
9	Solvent	CDCl3
10	Temperature	298.0
11	Pulse Sequence	hsqcetgp
12	Number of Scans	2
13	Receiver Gain	101
14	Relaxation Delay	1.5000
15	Pulse Width	10.8400
16	Acquisition Time	0.1024
17	Acquisition Date	2020-01-24T21:26:01
18	Modification Date	2020-01-27T00:15:38
19	Spectrometer Frequency	(500.20, 125.78)
20	Spectral Width	(5000.0, 20752.3)
21	Lowest Frequency	(-520.2, -943.0)
22	Nucleus	(1H, 13C)
23	Acquired Size	(512, 256)
24	Spectral Size	(1024, 1024)

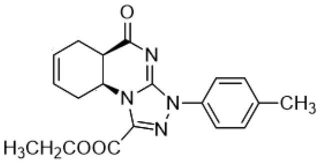

HMBC Experiments

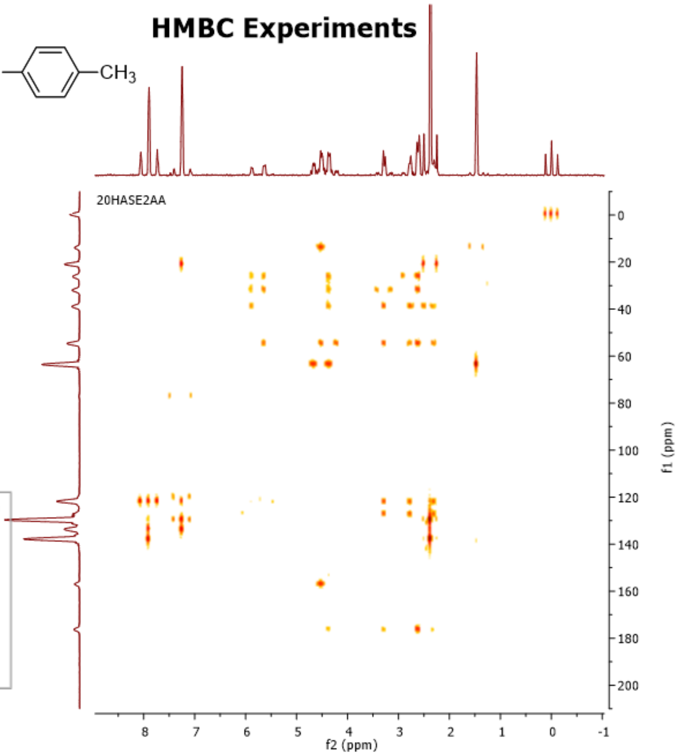

C-H9a Correlation	
f1 (ppm)	f2 (ppm)
26.24	4.35
31.78	4.36
38.70	4.36
61.12	4.37
153.31	4.36
176.19	4.36

	Parameter	Value (f2, f1)
1	Data File Name	E:/ after/ Hungary work/ 19 20HAS/ 20HASE/ E2/ 20HASE2AA-20 200127T074948 Z-001/ 20HASE2AA/ 16/ pdata/ 1/ 2rr
2	Title	20HASE2AA
3	Comment	
4	Origin	Bruker BioSpin GmbH
5	Owner	nmrsu
6	Site	
7	Spectrometer	CAB AV4 500 MHZ BASIC
8	Author	
9	Solvent	CDCl3
10	Temperature	298.0
11	Pulse Sequence	hmbcgpndqf
12	Number of Scans	8
13	Receiver Gain	101
14	Relaxation Delay	1.5000
15	Pulse Width	10.8400
16	Acquisition Time	0.2048
17	Acquisition Date	2020-01-24T21:57:30
18	Modification Date	2020-01-27T00:15:44
19	Spectrometer Frequency	(500.20, 125.78)
20	Spectral Width	(5000.0, 27672.1)
21	Lowest Frequency	(-520.2, -1258.5)
22	Nucleus	(1H, 13C)
23	Acquired Size	(1024, 128)
24	Spectral Size	(2048, 1024)

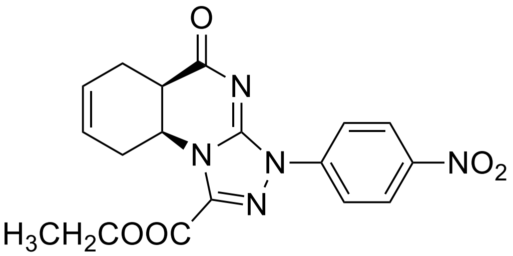

(4a*S**,8a*R**)-Ethyl 9-oxo-1-(4-nitrophenyl)-1,4a,5,8,8a,9-hexahydro-[1,2,4]triazolo[4,3-*a*]quinazoline-3-carboxylate (**4c**)

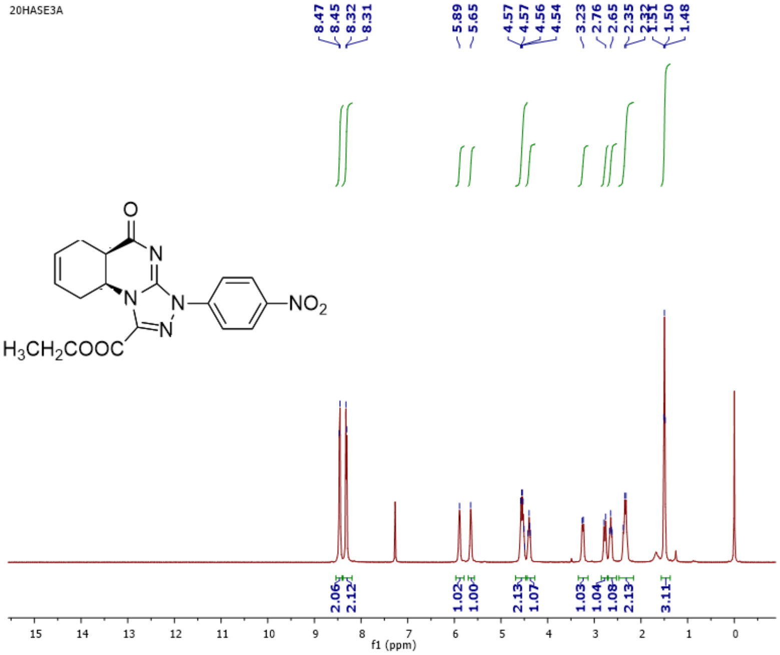

Parameter		Value
1	Data File Name	E:/ after/ Hungary work/ 19 20HAS/ 20HASE/ E3/ 20HASE3A-20200127T141455Z-001/ 20HASE3A/ 10/ pdata/ 1/ 1r
2	Title	20HASE3A
3	Comment	
4	Origin	Bruker BioSpin GmbH
5	Owner	nmrsu
6	Site	
7	Spectrometer	CAB AV4 500 MHZ BASIC
8	Author	
9	Solvent	CDCl3
10	Temperature	298.0
11	Pulse Sequence	zg30
12	Number of Scans	16
13	Receiver Gain	65
14	Relaxation Delay	1.0000
15	Pulse Width	10.8400
16	Acquisition Time	3.2768
17	Acquisition Date	2020-01-27T13:31:25
18	Modification Date	2020-01-27T05:59:54
19	Spectrometer Frequency	500.20
20	Spectral Width	10000.0
21	Lowest Frequency	-1920.2
22	Nucleus	1H
23	Acquired Size	32768
24	Spectral Size	65536

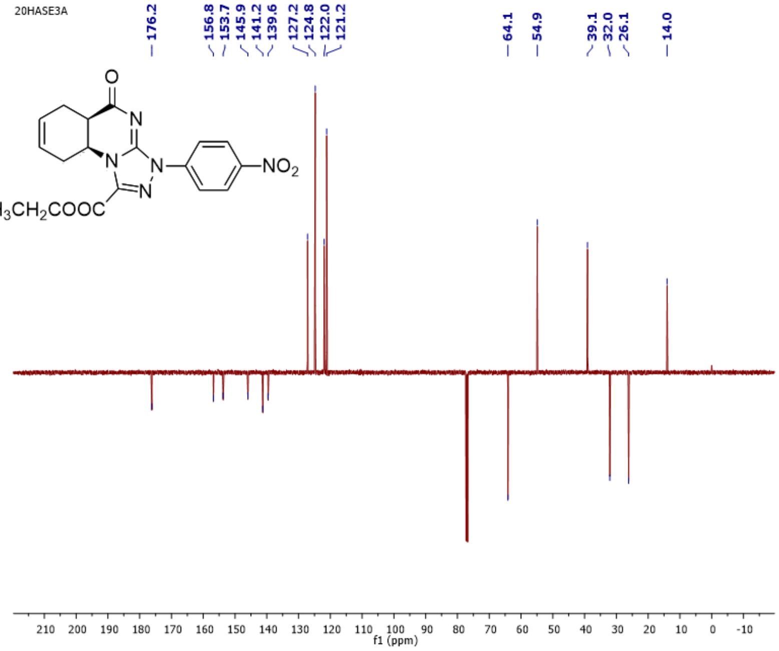

Parameter		Value
1	Data File Name	E:/ after/ Hungary work/ 19 20HAS/ 20HASE/ E3/ 20HASE3A-20200127T141455Z-001/ 20HASE3A/ 11/ pdata/ 1/ 1r
2	Title	20HASE3A
3	Comment	
4	Origin	Bruker BioSpin GmbH
5	Owner	nmrsu
6	Site	
7	Spectrometer	CAB AV4 500 MHZ BASIC
8	Author	
9	Solvent	CDCl3
10	Temperature	298.0
11	Pulse Sequence	jmod
12	Number of Scans	256
13	Receiver Gain	101
14	Relaxation Delay	2.0000
15	Pulse Width	10.0000
16	Acquisition Time	1.0879
17	Acquisition Date	2020-01-27T13:45:36
18	Modification Date	2020-01-27T06:00:14
19	Spectrometer Frequency	125.78
20	Spectral Width	30120.5
21	Lowest Frequency	-2482.7
22	Nucleus	13C
23	Acquired Size	32768
24	Spectral Size	32768

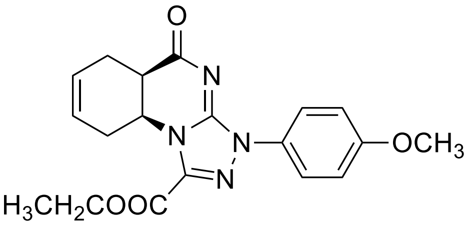

(4a*S**,8a*R**)-Ethyl 9-oxo-1-(4-methoxyphenyl)-1,4a,5,8,8a,9-hexahydro[1,2,4]triazolo-[4,3-*a*]quinazoline-3-carboxylate (**4d**)

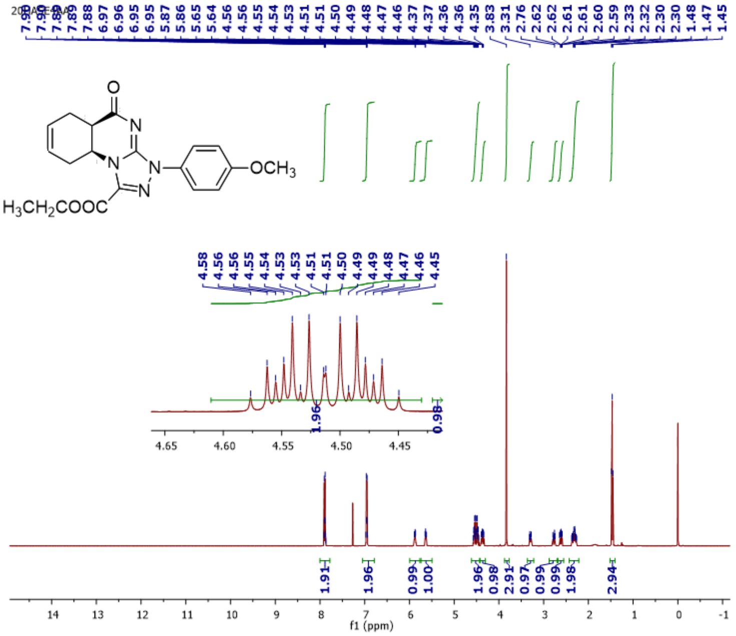

	Parameter	Value
1	Data File Name	E:/ after/ Hungary work/ 19 20HAS/ 20HASE/ E4/ 20HASE4AA-20 200129T12191 4Z-001/ 20HASE4AA/ 10/ pdata/ 1/ 1r
2	Title	20HASE4AA
3	Comment	
4	Origin	Bruker BioSpin GmbH
5	Owner	nmrsu
6	Site	
7	Spectrometer	CAB AV4 500 MHZ BASIC
8	Author	
9	Solvent	CDCl3
10	Temperature	298.0
11	Pulse Sequence	zg30
12	Number of Scans	16
13	Receiver Gain	83
14	Relaxation Delay	1.0000
15	Pulse Width	10.8400
16	Acquisition Time	3.2768
17	Acquisition Date	2020-01-28T15:36:37
18	Modification Date	2020-01-29T03:31:04
19	Spectrometer Frequency	500.20
20	Spectral Width	10000.0
21	Lowest Frequency	-1919.3
22	Nucleus	1H
23	Acquired Size	32768
24	Spectral Size	65536

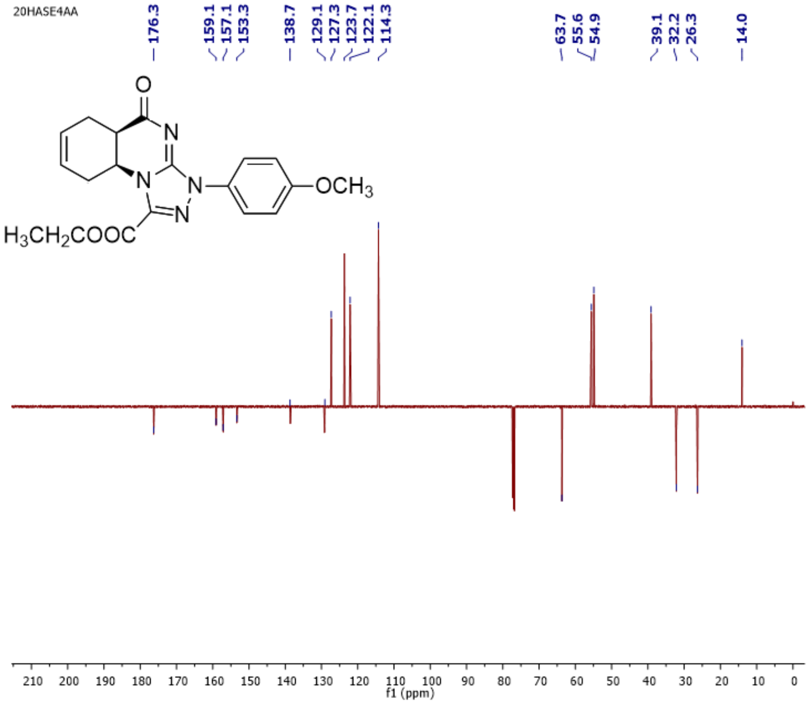

	Parameter	Value
1	Data File Name	E:/ after/ Hungary work/ 19 20HAS/ 20HASE/ E4/ 20HASE4AA-20 200129T12191 4Z-001/ 20HASE4AA/ 11/ pdata/ 1/ 1r
2	Title	20HASE4AA
3	Comment	
4	Origin	Bruker BioSpin GmbH
5	Owner	nmrsu
6	Site	
7	Spectrometer	CAB AV4 500 MHZ BASIC
8	Author	
9	Solvent	CDCl3
10	Temperature	298.0
11	Pulse Sequence	jmod
12	Number of Scans	256
13	Receiver Gain	101
14	Relaxation Delay	2.0000
15	Pulse Width	10.0000
16	Acquisition Time	1.0879
17	Acquisition Date	2020-01-28T15:50:47
18	Modification Date	2020-01-29T03:29:24
19	Spectrometer Frequency	125.78
20	Spectral Width	30120.5
21	Lowest Frequency	-2482.7
22	Nucleus	13C
23	Acquired Size	32768
24	Spectral Size	32768

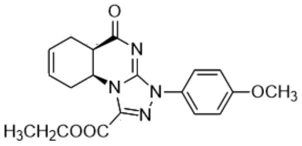

COSY Experiments

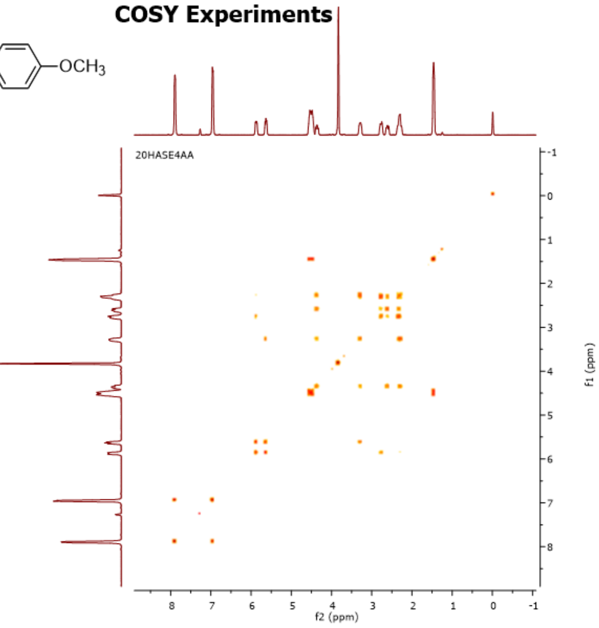

H-H correlation

f1 (ppm)	f2 (ppm)
2.61	2.32
2.77	2.60
2.77	2.34
3.29	2.29
4.35	3.29
4.36	2.30
4.36	2.61
4.51	1.46
5.63	3.29
5.86	2.28
5.88	2.77
5.88	5.63
7.90	6.95

	Parameter	Value (f2, f1)
1	Data File Name	E:/ after/ Hungary work/ 19 20HAS/ 20HASE/ E4/ 20HASE4AA-20200129T121914Z-001/ 20HASE4AA/ 12/ pdata/ 1/ 2rr
2	Title	20HASE4AA
3	Comment	
4	Origin	Bruker BioSpin GmbH
5	Owner	nmrsu
6	Site	
7	Spectrometer	CAB AV4 500 MHZ BASIC
8	Author	
9	Solvent	CDCl3
10	Temperature	298.0
11	Pulse Sequence	cosygpppqf
12	Number of Scans	1
13	Receiver Gain	101
14	Relaxation Delay	1.9508
15	Pulse Width	10.8400
16	Acquisition Time	0.2048
17	Acquisition Date	2020-01-28T15:56:36
18	Modification Date	2020-01-29T03:29:34
19	Spectrometer Frequency	(500.20, 500.20)
20	Spectral Width	(5000.0, 5000.0)
21	Lowest Frequency	(-544.7, -544.7)
22	Nucleus	(1H, 1H)
23	Acquired Size	(1024, 128)
24	Spectral Size	(1024, 1024)

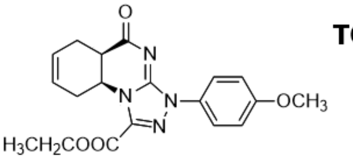

TOCSY Experiments

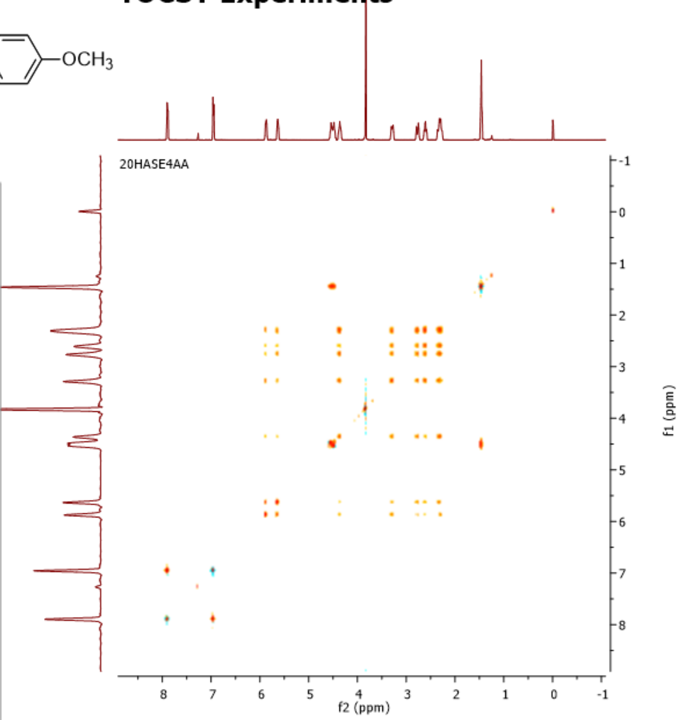

f1 (ppm)	f2 (ppm)
2.61	2.31
2.77	2.32
3.26	2.29
3.29	2.32
3.29	2.60
3.29	2.76
4.36	3.28
4.36	2.32
4.36	2.77
4.37	2.59
4.52	1.46
5.63	2.32
5.63	2.61
5.63	2.77
5.63	3.29
5.88	3.28
5.88	2.77
5.88	2.60
5.88	2.30
6.96	7.89
7.89	6.95

	Parameter	Value (f2, f1)
1	Data File Name	E:/ after/ Hungary work/ 19 20HAS/ 20HASE/ E4/ 20HASE4AA-20200129T121914Z-001/ 20HASE4AA/ 14/ pdata/ 1/ 2rr
2	Title	20HASE4AA
3	Comment	
4	Origin	Bruker BioSpin GmbH
5	Owner	nmrsu
6	Site	
7	Spectrometer	CAB AV4 500 MHZ BASIC
8	Author	
9	Solvent	CDCl3
10	Temperature	298.0
11	Pulse Sequence	mlevphpp
12	Number of Scans	8
13	Receiver Gain	101
14	Relaxation Delay	2.0000
15	Pulse Width	10.8400
16	Acquisition Time	0.2048
17	Acquisition Date	2020-01-28T18:02:47
18	Modification Date	2020-01-29T03:31:22
19	Spectrometer Frequency	(500.20, 500.20)
20	Spectral Width	(5000.0, 5000.0)
21	Lowest Frequency	(-544.7, -544.7)
22	Nucleus	(1H, 1H)
23	Acquired Size	(1024, 256)
24	Spectral Size	(1024, 1024)

HSQC Experiments

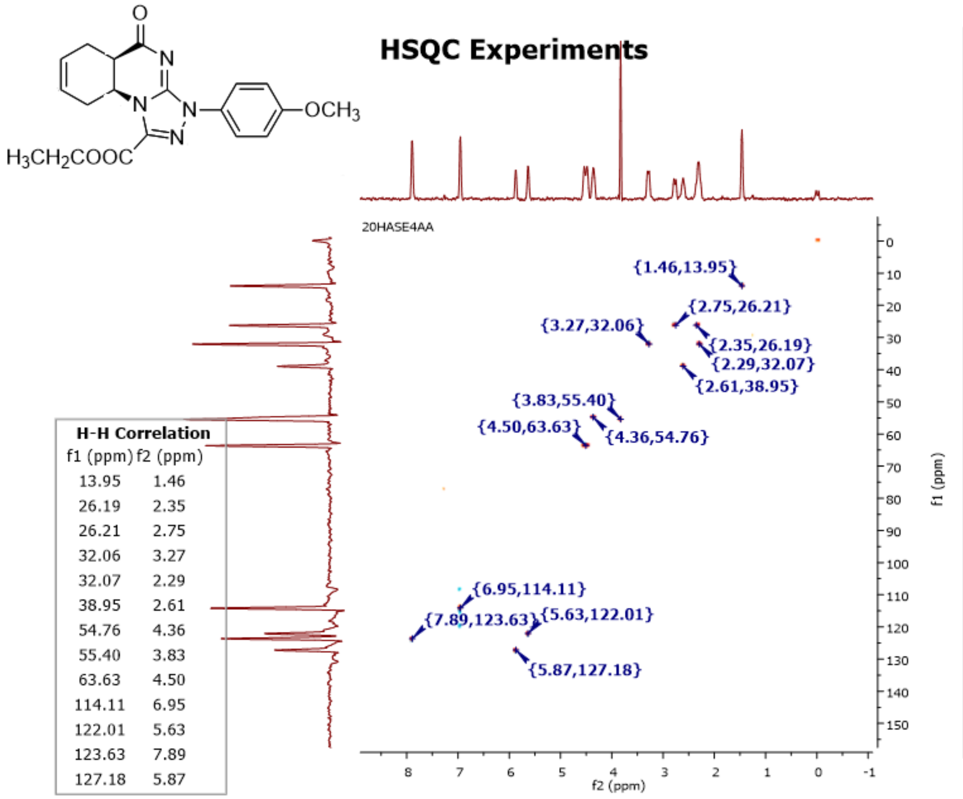

H-H Correlation

f1 (ppm)	f2 (ppm)
13.95	1.46
26.19	2.35
26.21	2.75
32.06	3.27
32.07	2.29
38.95	2.61
54.76	4.36
55.40	3.83
63.63	4.50
114.11	6.95
122.01	5.63
123.63	7.89
127.18	5.87

	Parameter	Value (f2, f1)
1	Data File Name	E:/ after/ Hungary work/ 19 20HAS/ 20HASE/ E4/ 20HASE4AA-20 200129T121914 Z-001/ 20HASE4AA/ 15/ pdata/ 1/ 2rr
2	Title	20HASE4AA
3	Comment	
4	Origin	Bruker BioSpin GmbH
5	Owner	nmrsu
6	Site	
7	Spectrometer	CAB AV4 500 MHZ BASIC
8	Author	
9	Solvent	CDCl3
10	Temperature	298.0
11	Pulse Sequence	hsqcetgp
12	Number of Scans	2
13	Receiver Gain	101
14	Relaxation Delay	1.5000
15	Pulse Width	10.8400
16	Acquisition Time	0.1024
17	Acquisition Date	2020-01-28T18:17:44
18	Modification Date	2020-01-29T03:32:10
19	Spectrometer Frequency	(500.20, 125.78)
20	Spectral Width	(5000.0, 20752.3)
21	Lowest Frequency	(-544.7, -943.0)
22	Nucleus	(1H, 13C)
23	Acquired Size	(512, 256)
24	Spectral Size	(1024, 1024)

HMBC Experiments

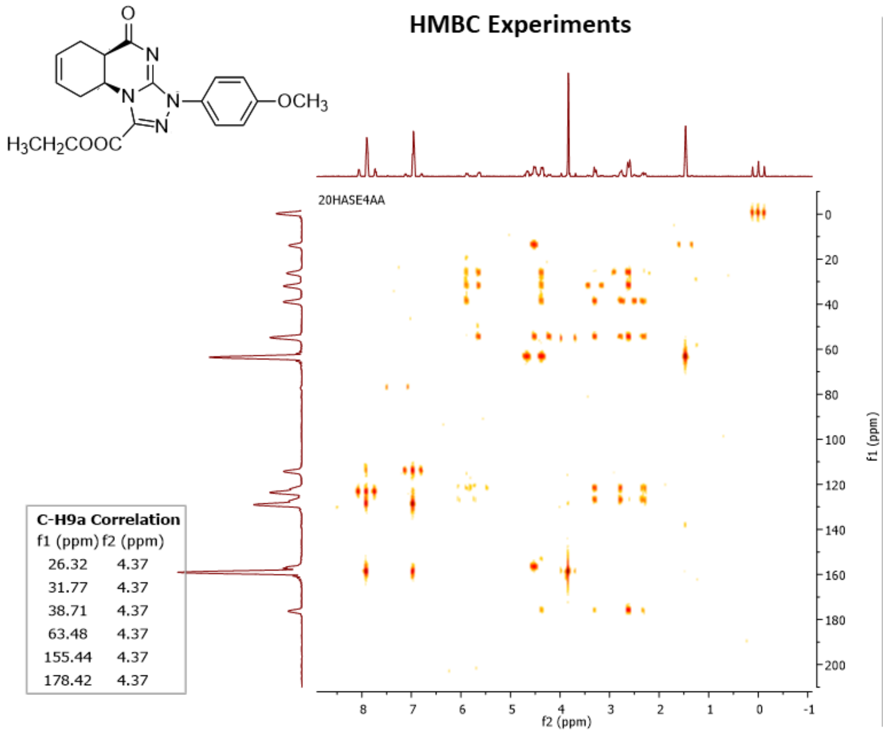

C-H9a Correlation

f1 (ppm)	f2 (ppm)
26.32	4.37
31.77	4.37
38.71	4.37
63.48	4.37
155.44	4.37
178.42	4.37

	Parameter	Value (f2, f1)
1	Data File Name	E:/ after/ Hungary work/ 19 20HAS/ 20HASE/ E4/ 20HASE4AA-20 200129T12191 4Z-001/ 20HASE4AA/ 16/ pdata/ 1/ 2rr
2	Title	20HASE4AA
3	Comment	
4	Origin	Bruker BioSpin GmbH
5	Owner	nmrsu
6	Site	
7	Spectrometer	CAB AV4 500 MHZ BASIC
8	Author	
9	Solvent	CDCl3
10	Temperature	298.0
11	Pulse Sequence	hmbcgpndqf
12	Number of Scans	8
13	Receiver Gain	101
14	Relaxation Delay	1.5000
15	Pulse Width	10.8400
16	Acquisition Time	0.2048
17	Acquisition Date	2020-01-28T18:49:12
18	Modification Date	2020-01-29T03:32:20
19	Spectrometer Frequency	(500.20, 125.78)
20	Spectral Width	(5000.0, 27672.1)
21	Lowest Frequency	(-544.7, -1258.5)
22	Nucleus	(1H, 13C)
23	Acquired Size	(1024, 128)
24	Spectral Size	(2048, 1024)

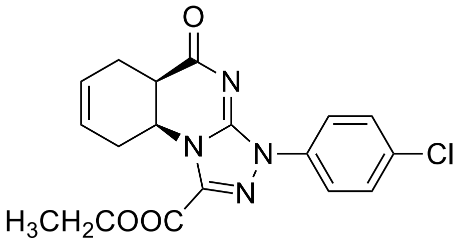

(4a*S**,8a*R**)-Ethyl 9-oxo-1-(4-chlorophenyl)-1,4a,5,8,8a,9-hexahydro[1,2,4]triazolo[4,3-*a*]quinazoline-3-carboxylate (**4e**)

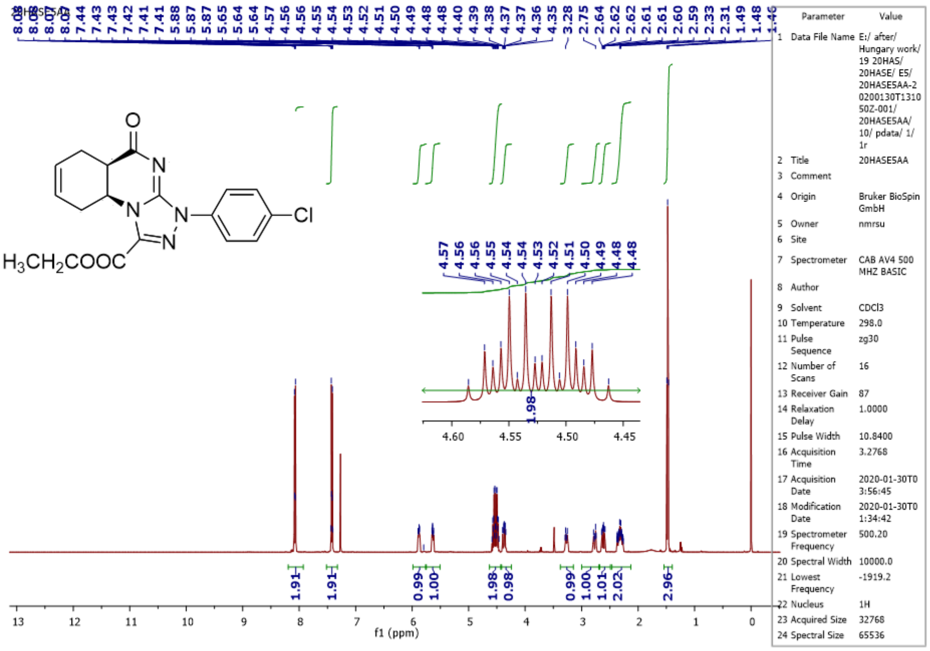

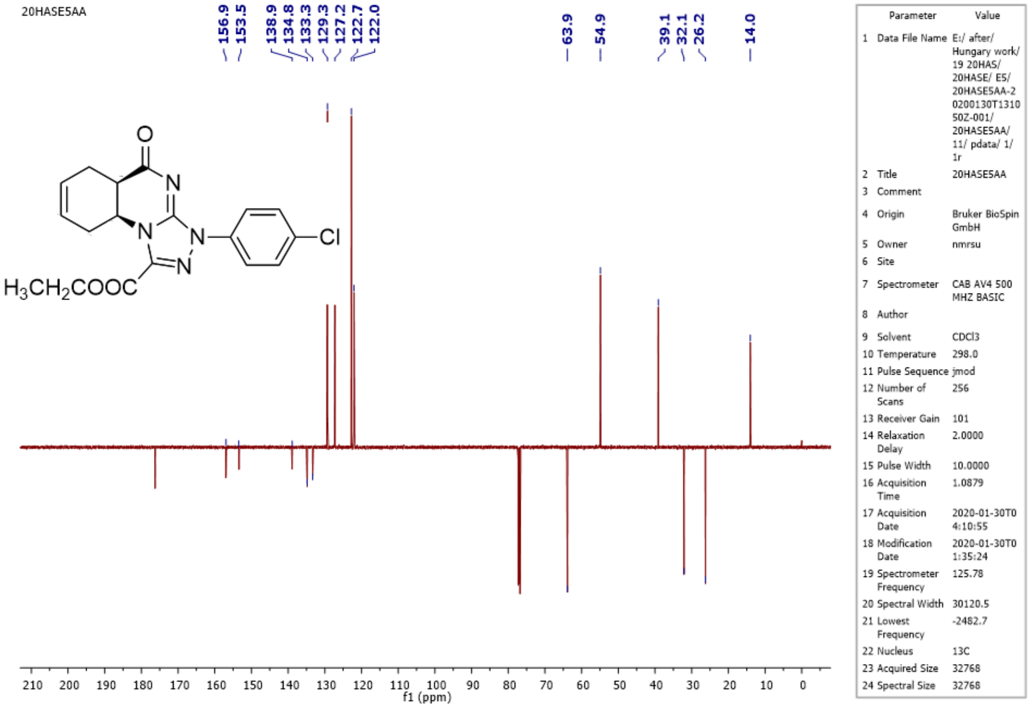

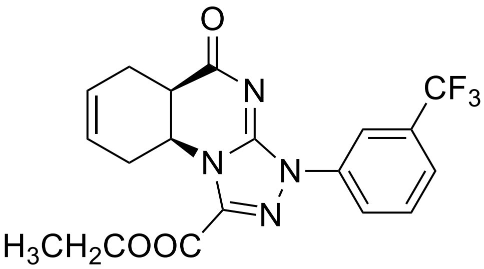

(4a*S**,8a*R**)-Ethyl 9-oxo-1-(3-(trifluoromethyl)phenyl)-1,4a,5,8,8a,9-hexahydro[1,2,4]triazolo[4,3-*a*]quinazoline-3-carboxylate (**4f**)

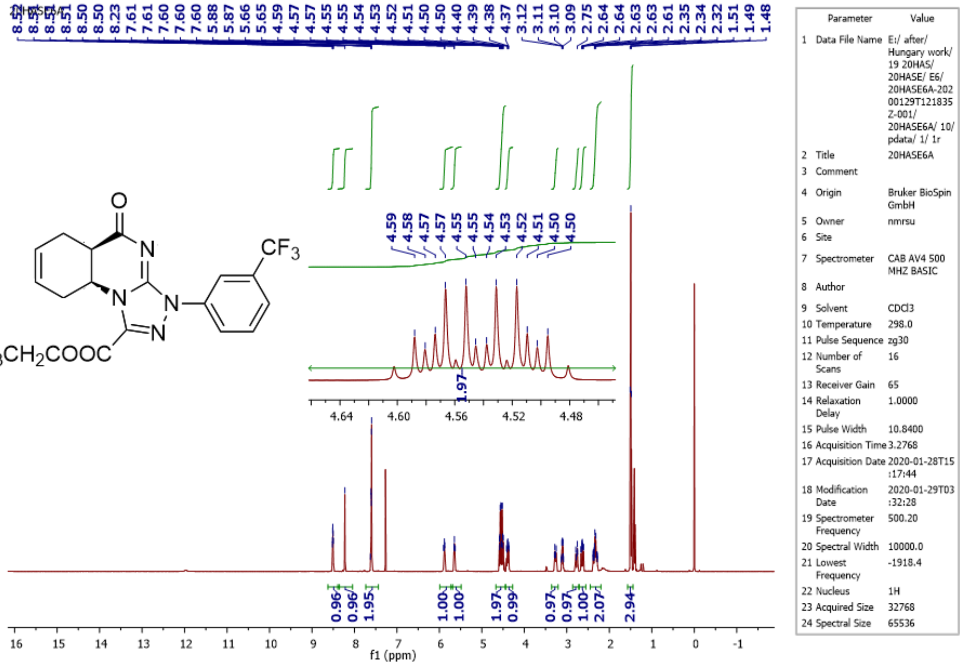

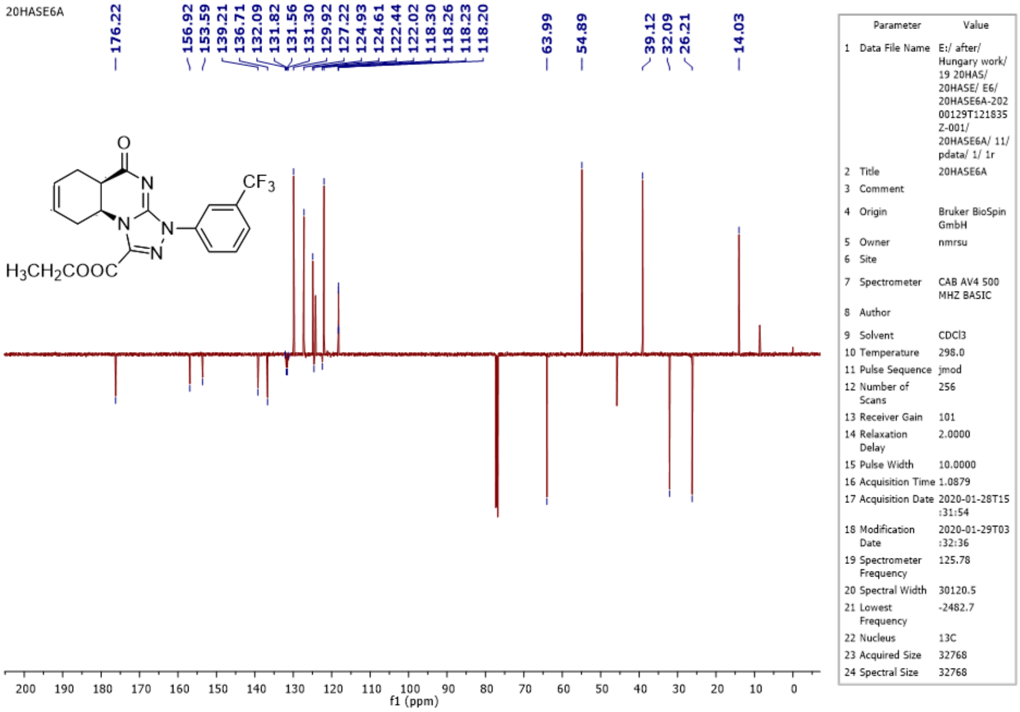

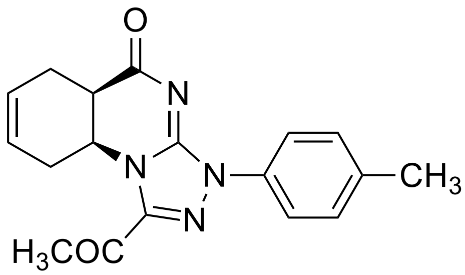

(4a*S**,8a*R**)-3-Acetyl-1-(*p*-tolyl)-4a,5,8,9-tetrahydro[1,2,4]triazolo[4,3-*a*]quinazoline-9(1*H*)-one (**4g**)

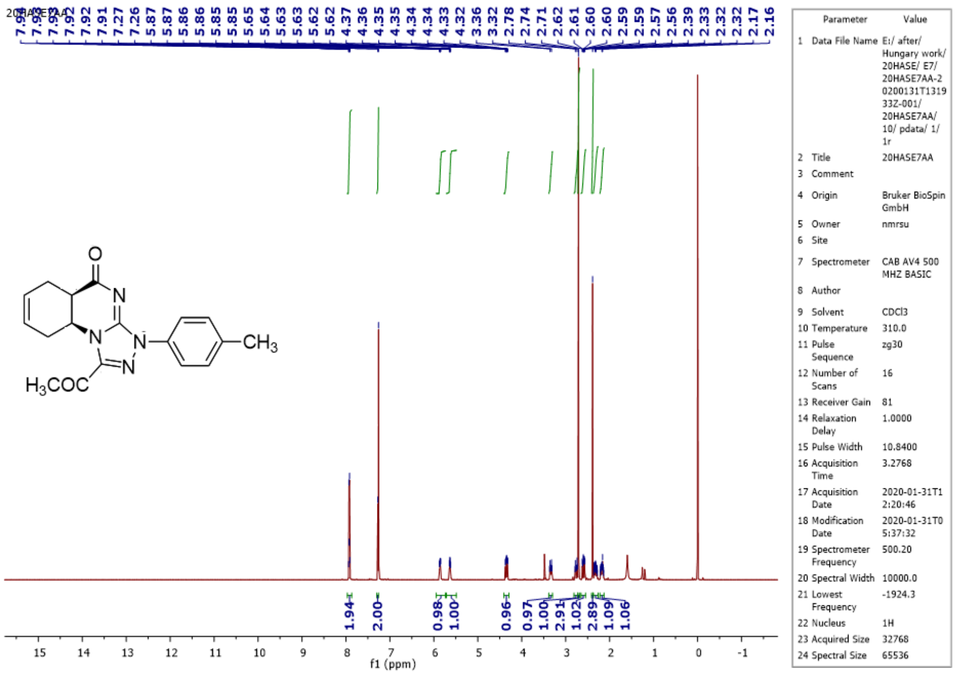

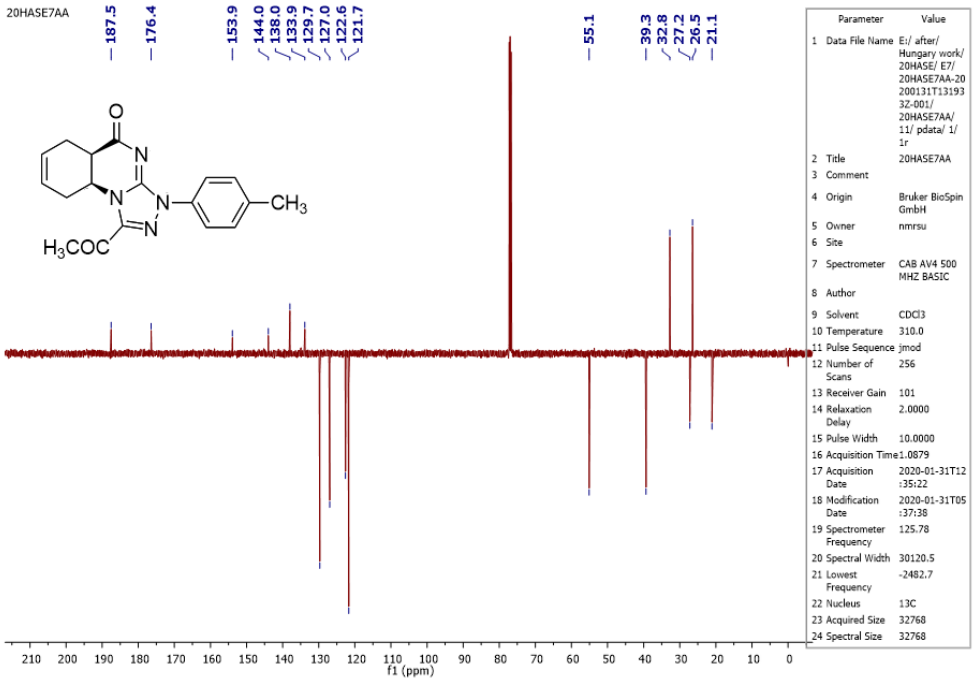

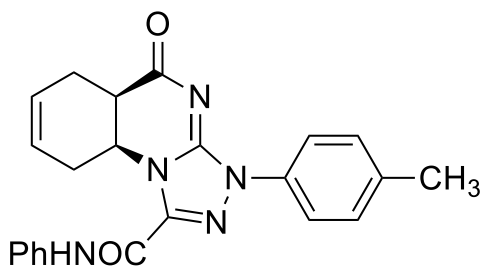

(4a*S**,8a*R**)-9-Oxo-*N*-Phenyl-1-(*p*-tolyl)-1,4a,5,8,8a,9-hexahydro[1,2,4]triazolo[4,3-*a*]quinazoline-3-carboxamide (**4h**)

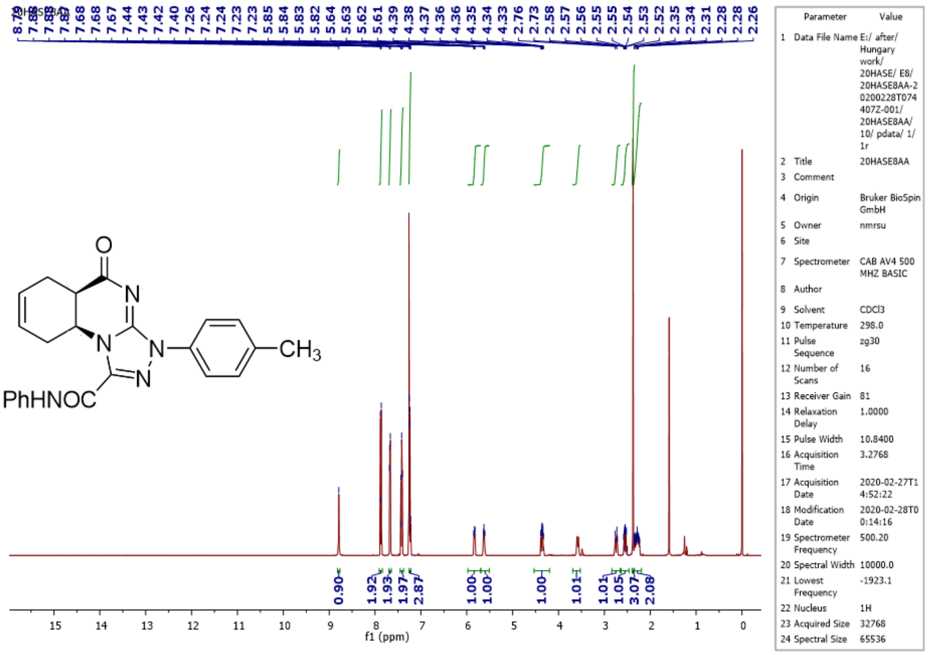

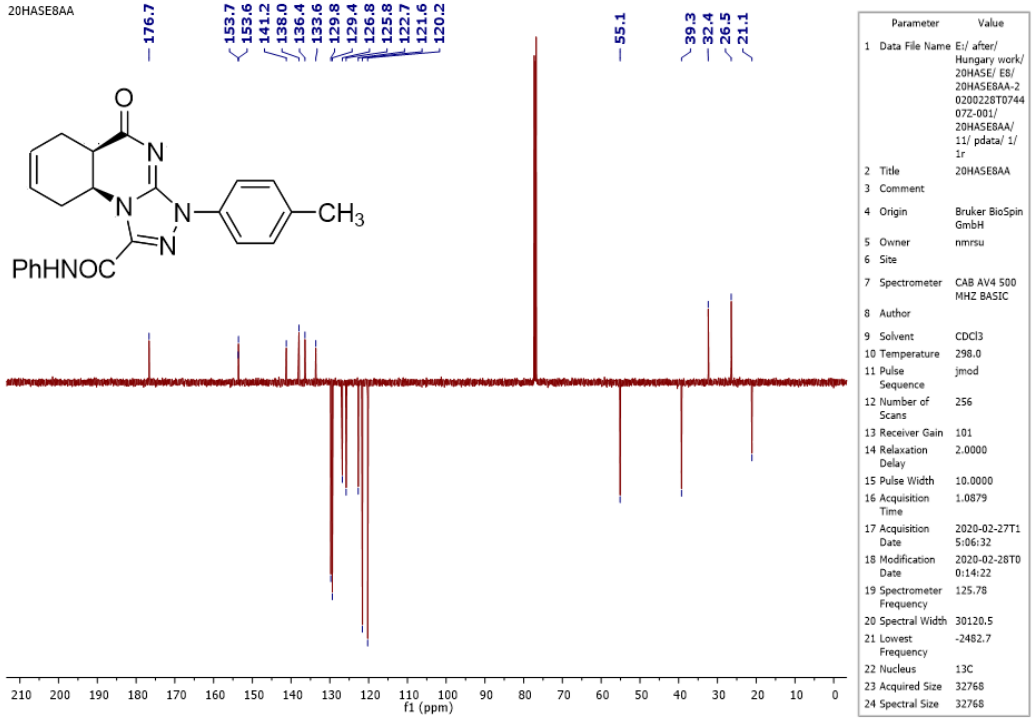

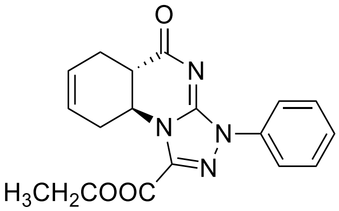

(4a*S**,8a*S**)-Ethyl 9-oxo-1-phenyl-1,4a,5,8,8a,9-hexahydro-[1,2,4]triazolo[4,3-a]quinazoline-3-carboxylate (**5a**)

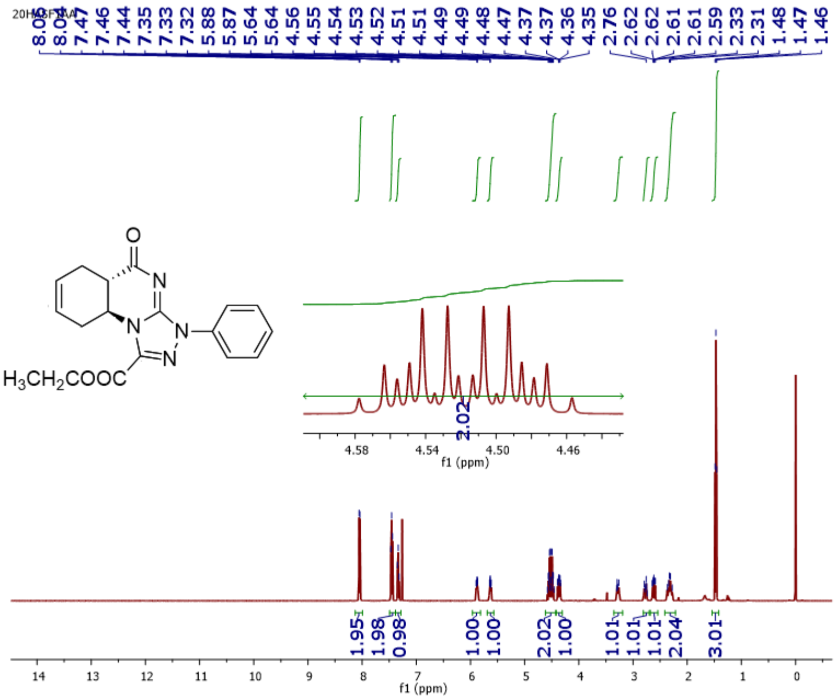

Parameter	Value
1 Data File Name	E:/ after/ Hungary work/ 19 20HAS/ 20HASF/ F1/ 20HASF1AA-20200131T160450Z-001/ 20HASF1AA/ 10/ pdata/ 1/ 1r
2 Title	20HASF1AA
3 Comment	
4 Origin	Bruker BioSpin GmbH
5 Owner	nmrsu
6 Site	
7 Spectrometer	CAB AV4 500 MHZ BASIC
8 Author	
9 Solvent	CDCl3
10 Temperature	310.0
11 Pulse Sequence	zg30
12 Number of Scans	16
13 Receiver Gain	81
14 Relaxation Delay	1.0000
15 Pulse Width	10.8400
16 Acquisition Time	3.2768
17 Acquisition Date	2020-01-31T12:39:50
18 Modification Date	2020-01-31T07:09:32
19 Spectrometer Frequency	500.20
20 Spectral Width	10000.0
21 Lowest Frequency	-1922.7
22 Nucleus	1H
23 Acquired Size	32768
24 Spectral Size	65536

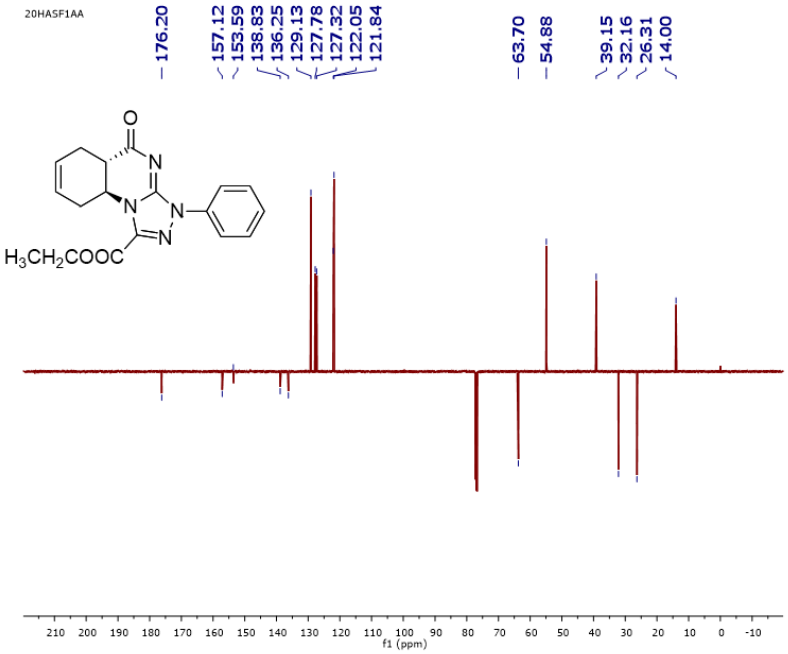

Parameter	Value
1 Data File Name	E:/ after/ Hungary work/ 19 20HAS/ 20HASF/ F1/ 20HASF1AA-20200131T160450Z-001/ 20HASF1AA/ 11/ pdata/ 1/ 1r
2 Title	20HASF1AA
3 Comment	
4 Origin	Bruker BioSpin GmbH
5 Owner	nmrsu
6 Site	
7 Spectrometer	CAB AV4 500 MHZ BASIC
8 Author	
9 Solvent	CDCl3
10 Temperature	310.0
11 Pulse Sequence	jmod
12 Number of Scans	256
13 Receiver Gain	101
14 Relaxation Delay	2.0000
15 Pulse Width	10.0000
16 Acquisition Time	1.0879
17 Acquisition Date	2020-01-31T12:54:15
18 Modification Date	2020-01-31T07:09:40
19 Spectrometer Frequency	125.78
20 Spectral Width	30120.5
21 Lowest Frequency	-2482.7
22 Nucleus	13C
23 Acquired Size	32768
24 Spectral Size	32768

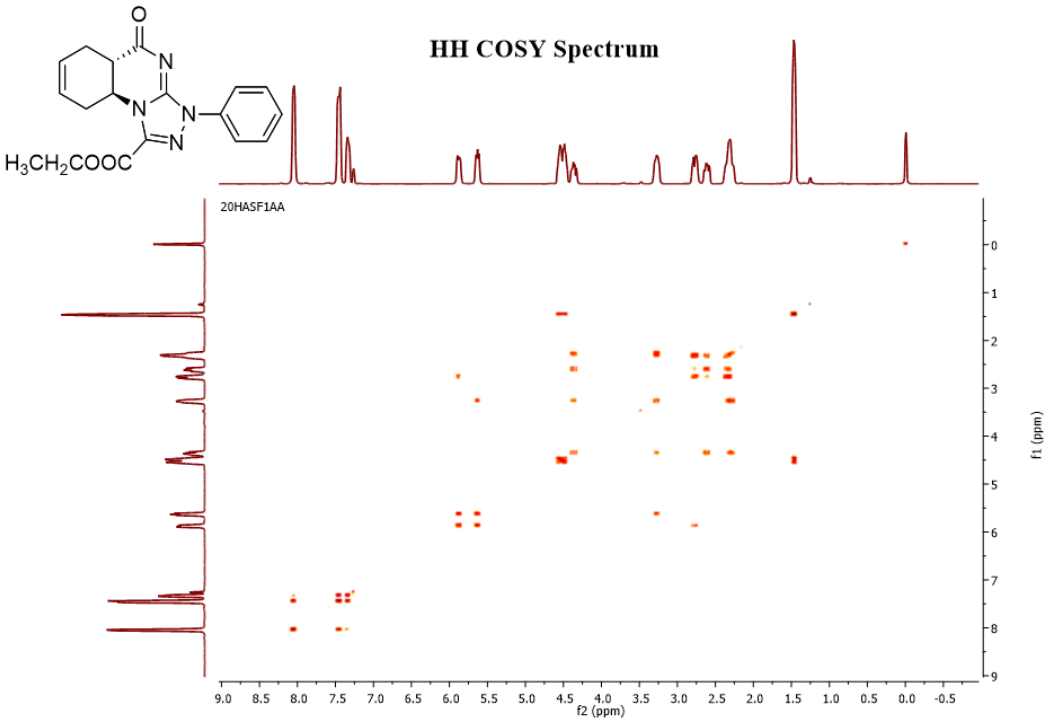

HH COSY Spectrum
H3CH2COOC
20HASF1AA
f2 (ppm)
f1 (ppm)

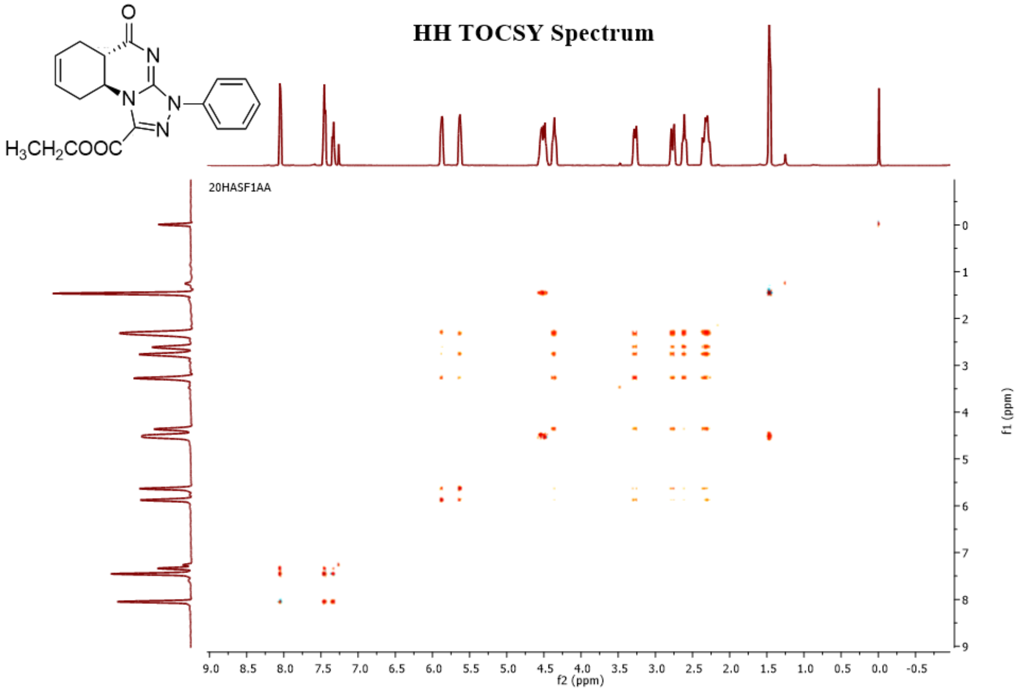

HH TOCSY Spectrum
H3CH2COOC
20HASF1AA
f2 (ppm)
f1 (ppm)

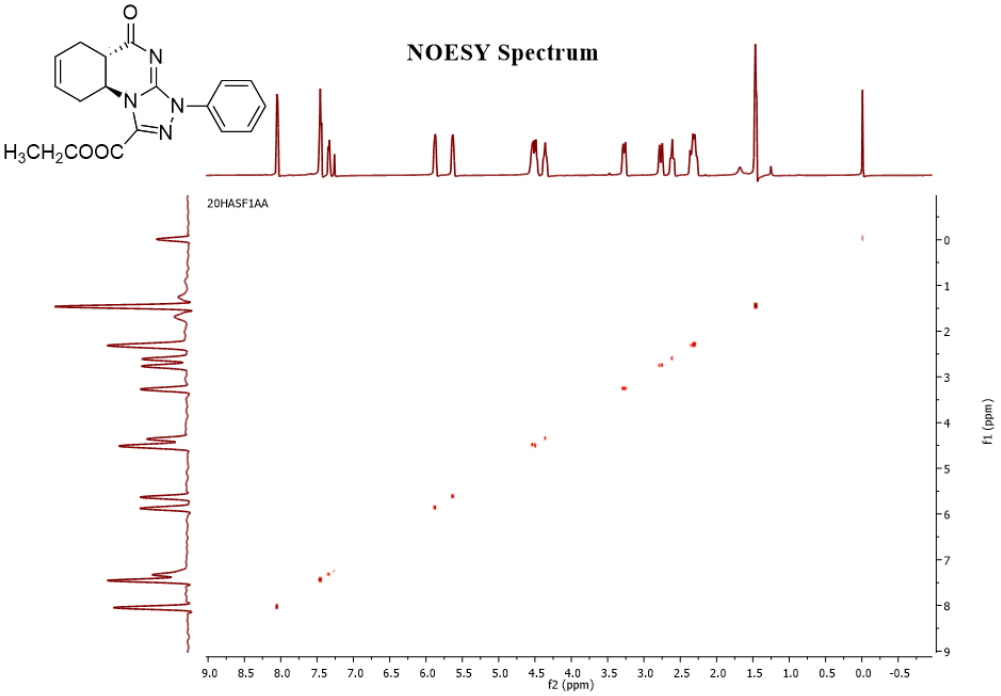
NOESY Spectrum
H3CH2COOC
O
N
N
N
N
N
20HASF1AA
f2 (ppm)
f1 (ppm)

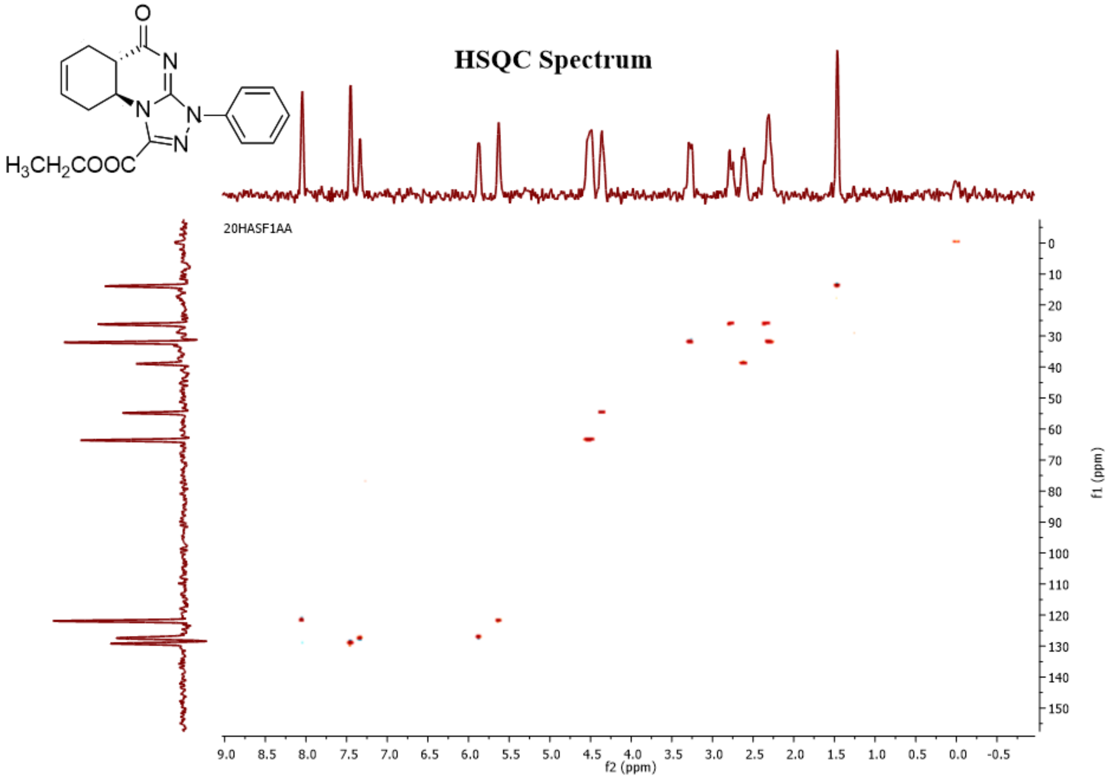
HSQC Spectrum
H3CH2COOC
O
N
N
N
N
N
20HASF1AA
f2 (ppm)
f1 (ppm)

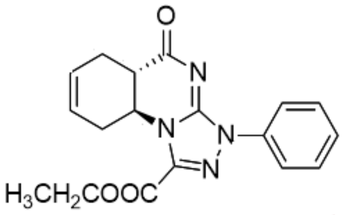

HMBC Spectrum

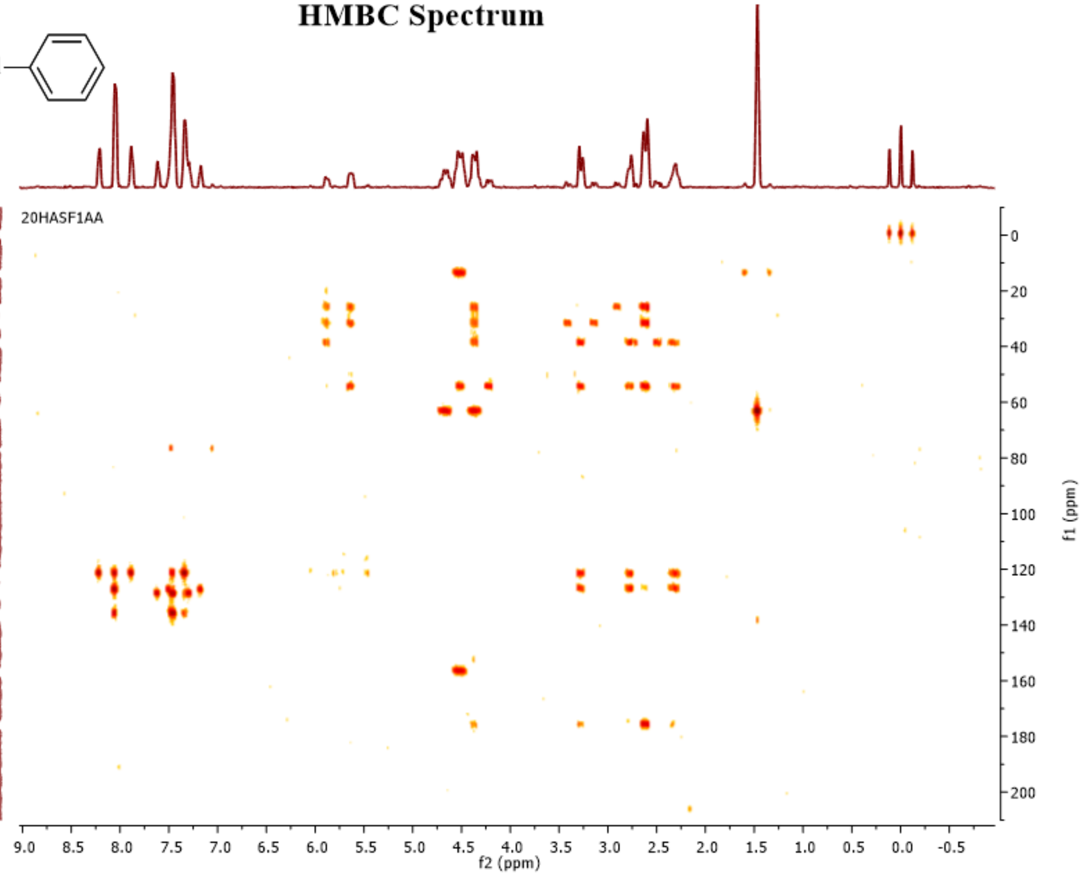

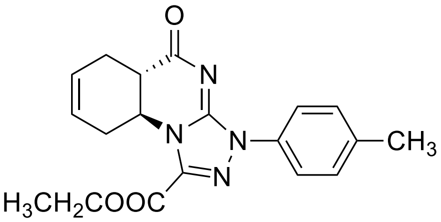

(4a*S**,8a*S**)-Ethyl 9-oxo-1-(*p*-tolyl)-1,4a,5,8,8a,9-hexahydro-[1,2,4]triazolo[4,3-*a*]quinazoline-3-carboxylate (**5b**)

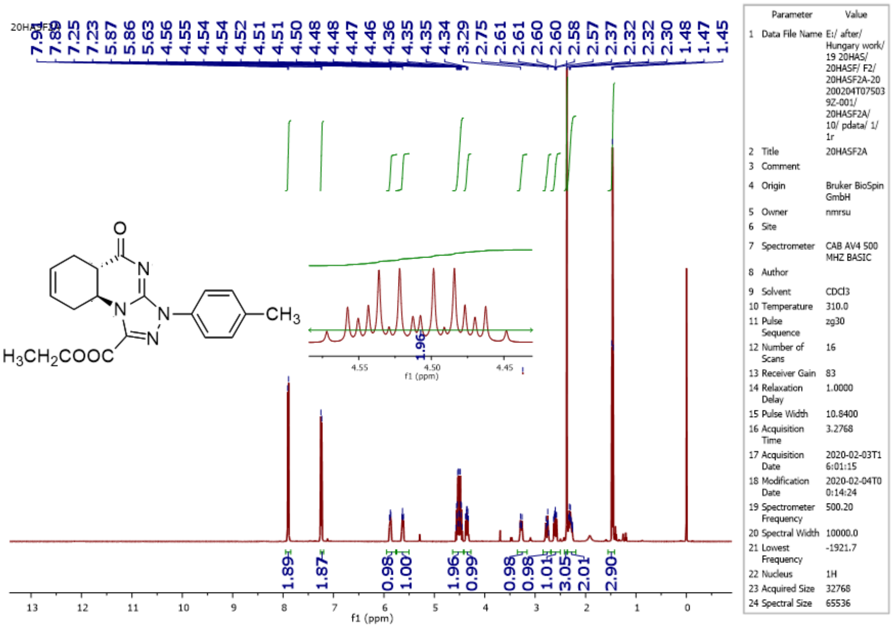

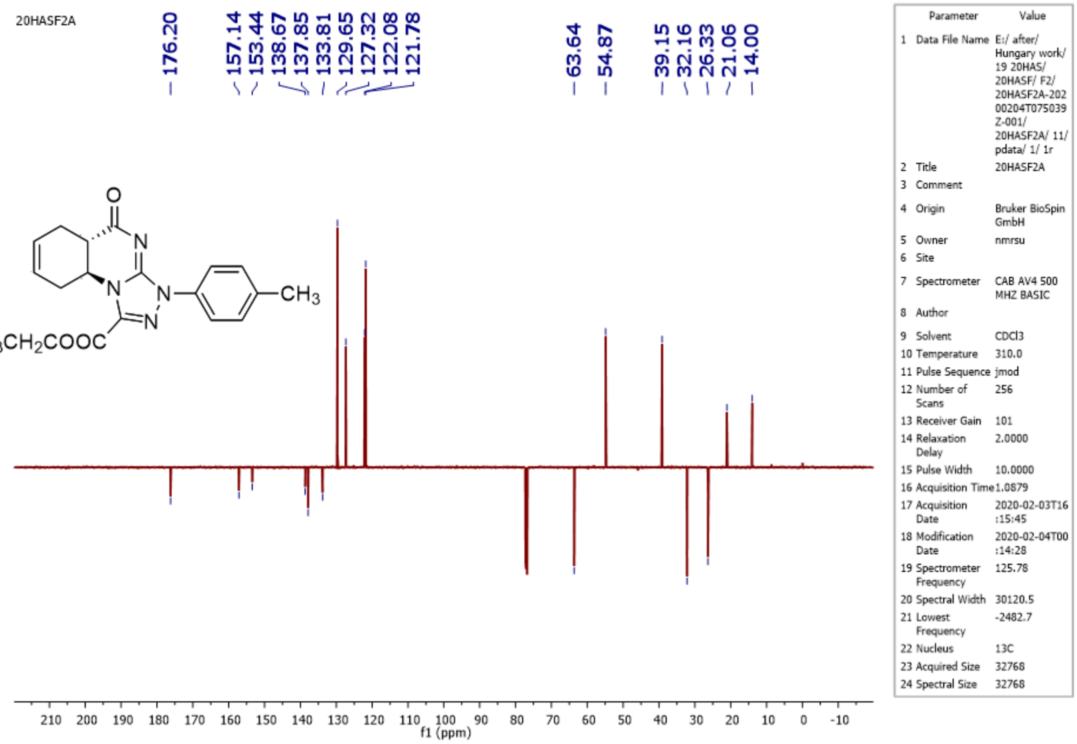

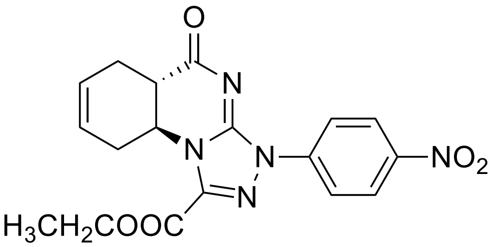

(4a*S**,8a*S**)-Ethyl 1-(4-nitrophenyl)-9-oxo-1,4a,5,8,8a,9-hexahydro-[1,2,4]triazolo[4,3-*a*]quinazoline-3-carboxylate (**5c**)

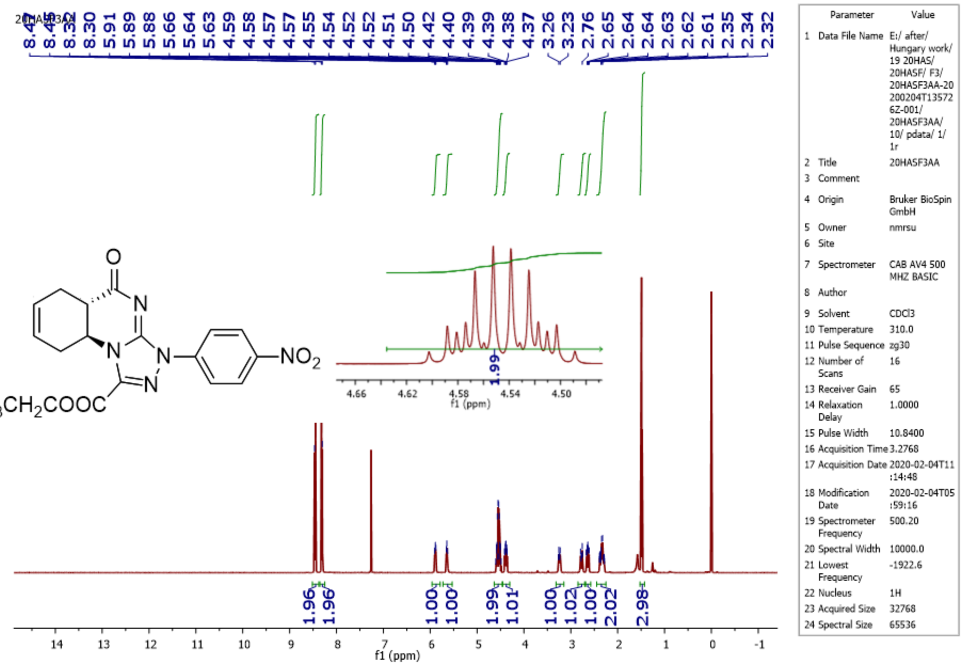

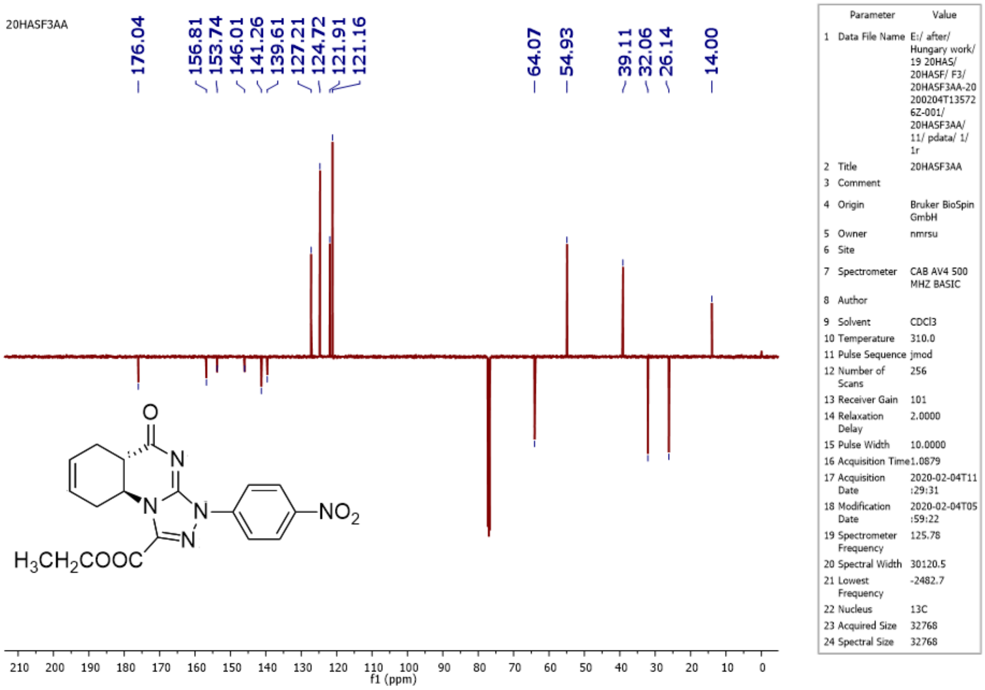

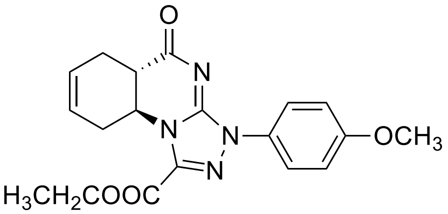

(4a*S**,8a*S**)-Ethyl 1-(4-methoxyphenyl)-9-oxo-1,4a,5,8,8a,9-hexahydro-[1,2,4]triazolo[4,3-*a*]quinazoline-3-carboxylate (**5d**)

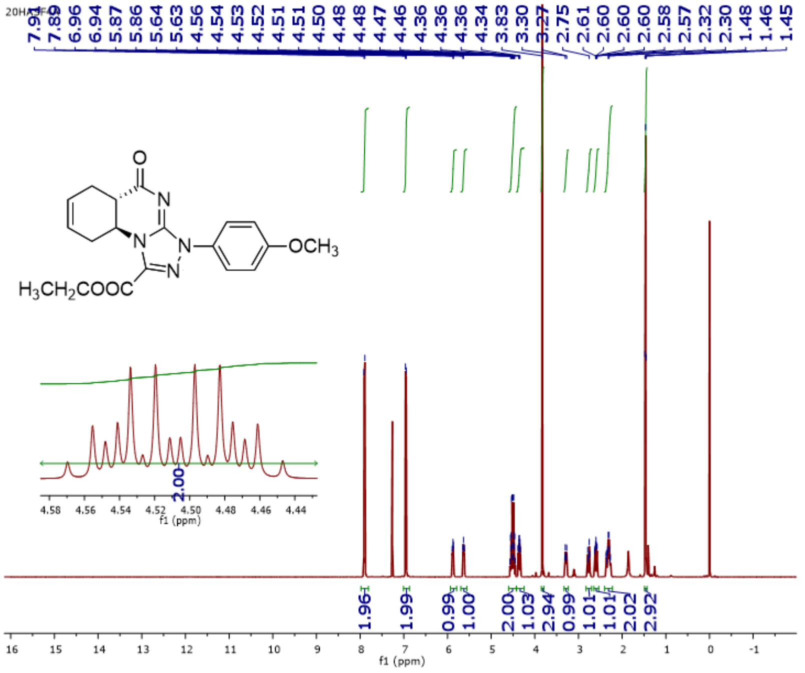

	Parameter	Value
1	Data File Name	E:/ after/ Hungary work/ 19 20HAS/ 20HASF/ F4/ 20HASF4A-20 200204T1358 12Z-001/ 20HASF4A/ 10/ pdata/ 1/ 1r
2	Title	20HASF4A
3	Comment	
4	Origin	Bruker BioSpin GmbH
5	Owner	nmrsu
6	Site	
7	Spectrometer	CAB AV4 500 MHZ BASIC
8	Author	
9	Solvent	CDCl3
10	Temperature	310.0
11	Pulse Sequence	zg30
12	Number of Scans	16
13	Receiver Gain	85
14	Relaxation Delay	1.0000
15	Pulse Width	10.8400
16	Acquisition Time	3.2768
17	Acquisition Date	2020-02-04T11:34:46
18	Modification Date	2020-02-04T05:59:02
19	Spectrometer Frequency	500.20
20	Spectral Width	10000.0
21	Lowest Frequency	-1922.0
22	Nucleus	1H
23	Acquired Size	32768
24	Spectral Size	65536

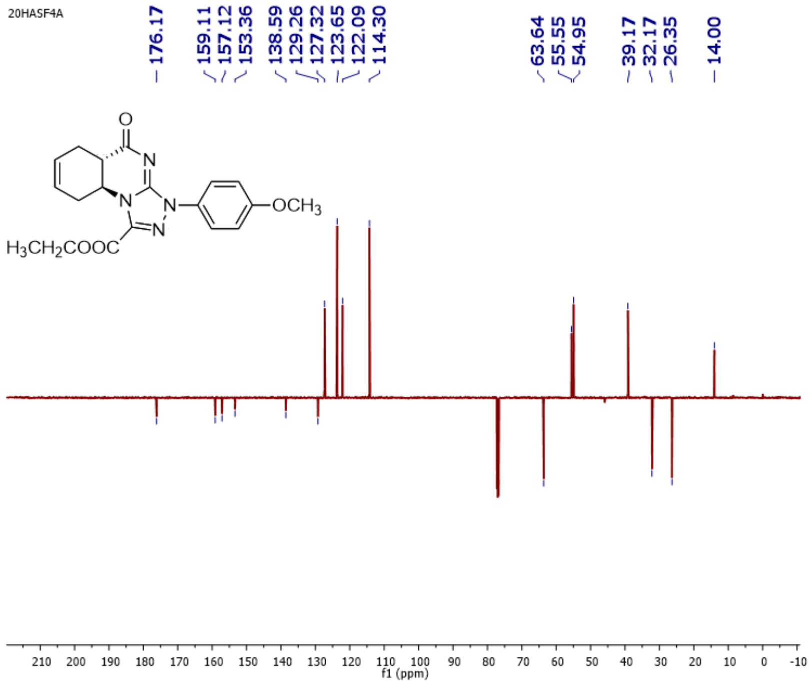

	Parameter	Value
1	Data File Name	E:/ after/ Hungary work/ 19 20HAS/ 20HASF/ F4/ 20HASF4A-202 00204T135812 Z-001/ 20HASF4A/ 11/ pdata/ 1/ 1r
2	Title	20HASF4A
3	Comment	
4	Origin	Bruker BioSpin GmbH
5	Owner	nmrsu
6	Site	
7	Spectrometer	CAB AV4 500 MHZ BASIC
8	Author	
9	Solvent	CDCl3
10	Temperature	310.0
11	Pulse Sequence	jmod
12	Number of Scans	256
13	Receiver Gain	101
14	Relaxation Delay	2.0000
15	Pulse Width	10.0000
16	Acquisition Time	1.0879
17	Acquisition Date	2020-02-04T11:48:55
18	Modification Date	2020-02-04T05:59:10
19	Spectrometer Frequency	125.78
20	Spectral Width	30120.5
21	Lowest Frequency	-2482.7
22	Nucleus	13C
23	Acquired Size	32768
24	Spectral Size	32768

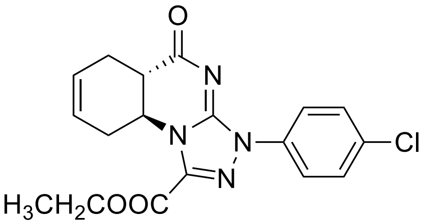

(4a*S**,8a*S**)-Ethyl 1-(4-chlorophenyl)-9-oxo-1,4a,5,8,8a,9-hexahydro-[1,2,4]triazolo[4,3-*a*]quinazoline-3-carboxylate (**5e**)

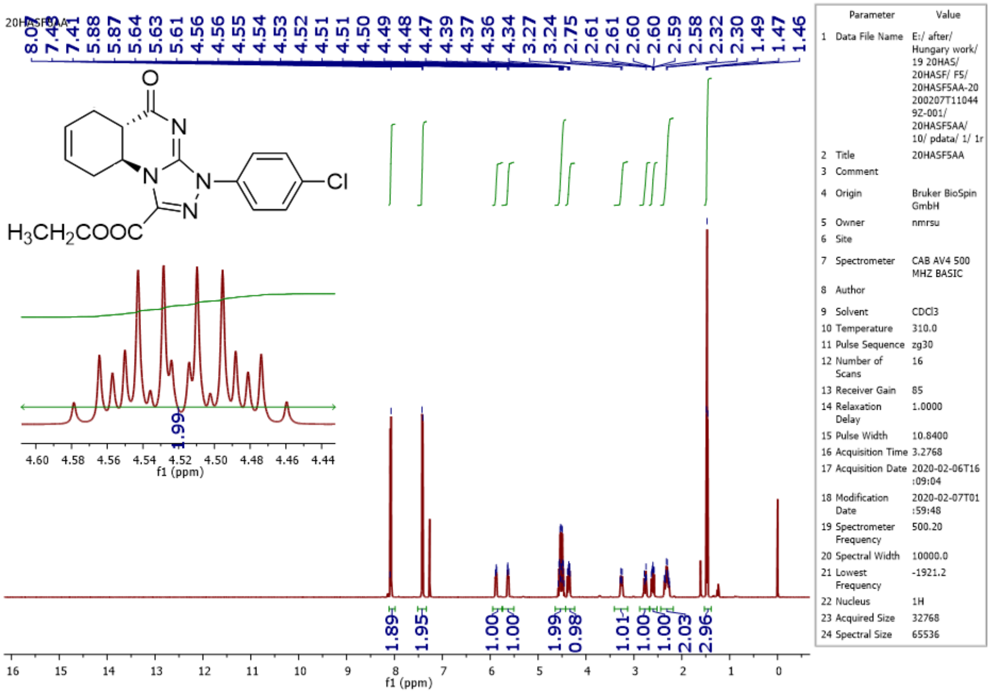

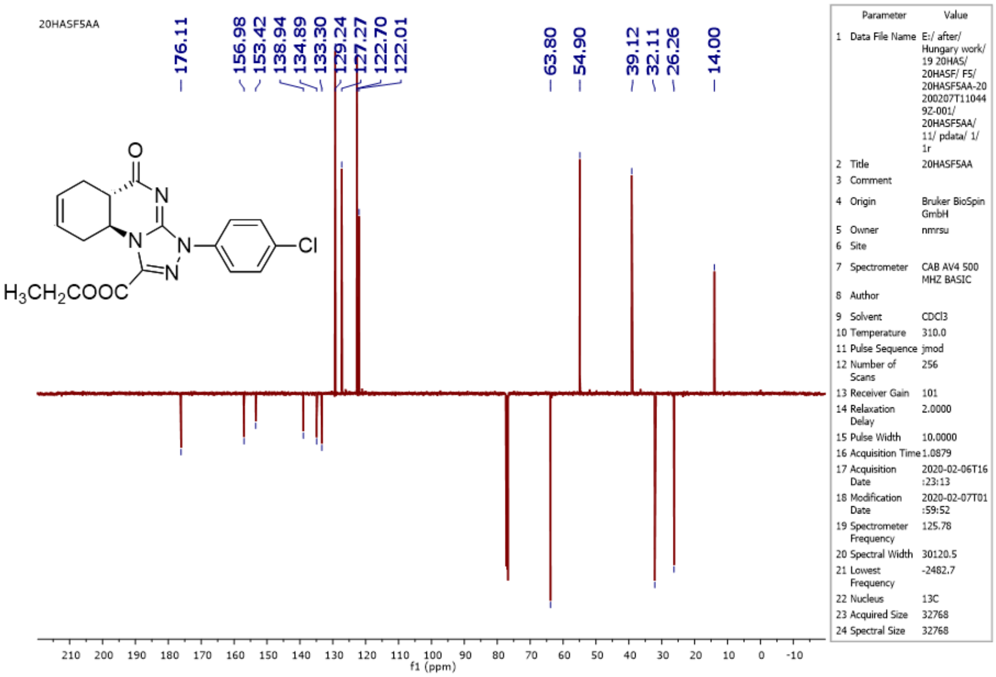

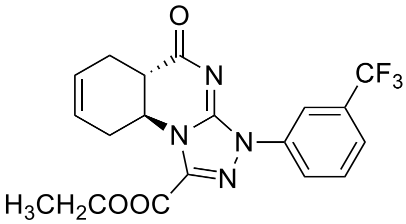

(4a*S**,8a*S**)-Ethyl 9-oxo-1-(3-(trifluoromethyl)phenyl)-1,4a,5,8,8a,9-hexahydro-[1,2,4]triazolo[4,3-*a*]quinazoline-3-carboxylate (**5f**)

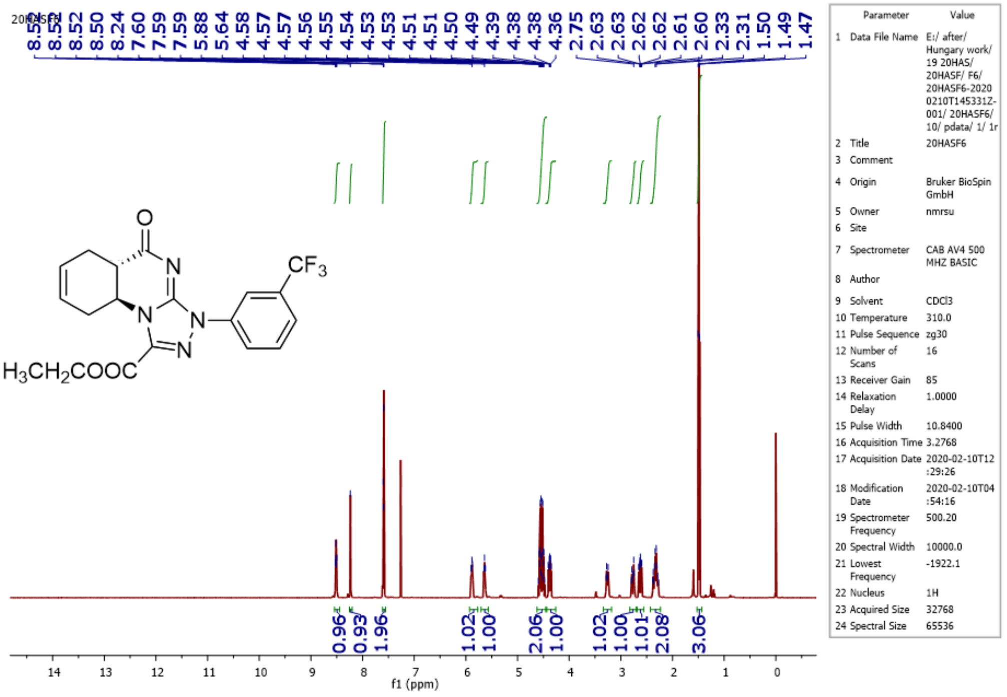

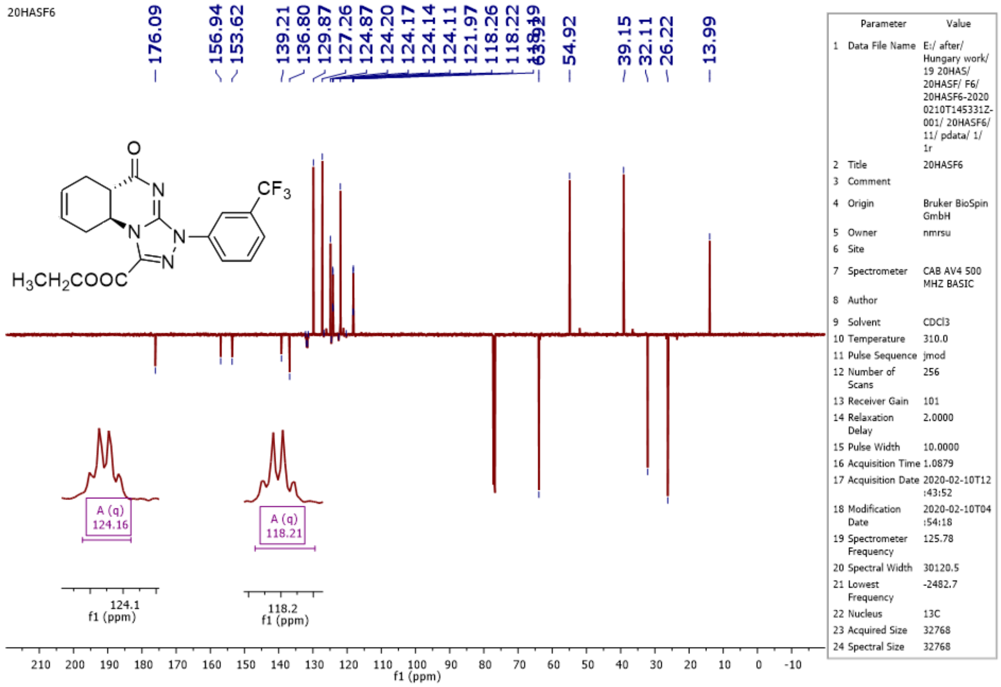

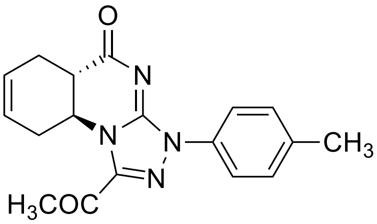

(4a*S**,8a*S**)-3-Acetyl-1-(*p*-tolyl)-4a,5,8,9-tetrahydro-[1,2,4]triazolo[4,3-*a*]quinazolin-9(1*H*)-one (**5g**)

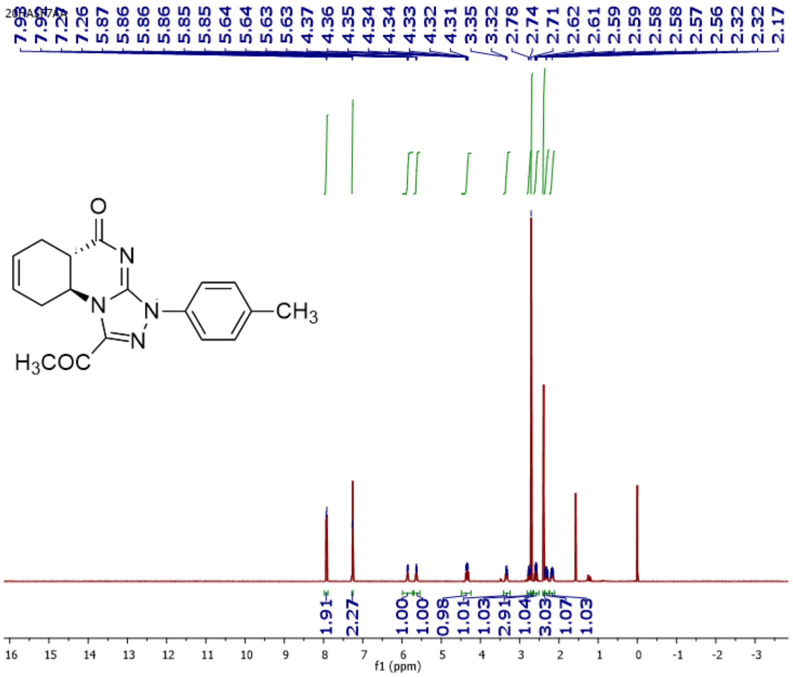

	Parameter	Value
1	Data File Name	E:/ after/ Hungary work/ 19 20HAS/ 20HASF/ F7/ 20HASF7AA-20 200207T13071 1Z-001/ 20HASF7AA/ 10/ pdata/ 1/ 1r
2	Title	20HASF7AA
3	Comment	
4	Origin	Bruker BioSpin GmbH
5	Owner	nmrsu
6	Site	
7	Spectrometer	CAB AV4 500 MHZ BASIC
8	Author	
9	Solvent	CDCl3
10	Temperature	310.0
11	Pulse Sequence	zg30
12	Number of Scans	16
13	Receiver Gain	65
14	Relaxation Delay	1.0000
15	Pulse Width	10.8400
16	Acquisition Time	3.2768
17	Acquisition Date	2020-02-07T11:33:08
18	Modification Date	2020-02-07T06:02:34
19	Spectrometer Frequency	500.20
20	Spectral Width	10000.0
21	Lowest Frequency	-1923.8
22	Nucleus	1H
23	Acquired Size	32768
24	Spectral Size	65536

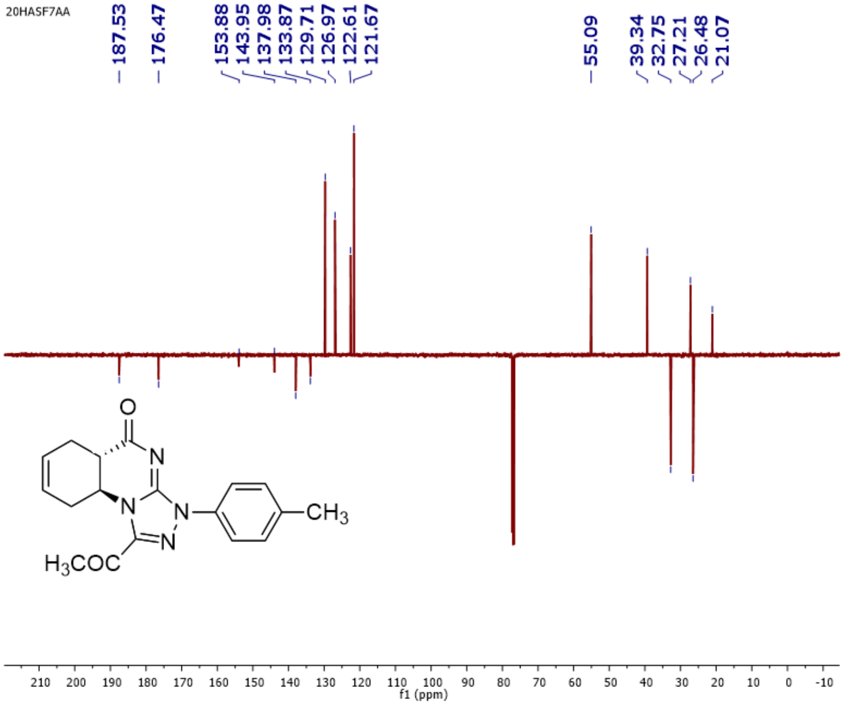

	Parameter	Value
1	Data File Name	E:/ after/ Hungary work/ 19 20HAS/ 20HASF/ F7/ 20HASF7AA-2 0200207T1307 11Z-001/ 20HASF7AA/ 11/ pdata/ 1/ 1r
2	Title	20HASF7AA
3	Comment	
4	Origin	Bruker BioSpin GmbH
5	Owner	nmrsu
6	Site	
7	Spectrometer	CAB AV4 500 MHZ BASIC
8	Author	
9	Solvent	CDCl3
10	Temperature	310.0
11	Pulse Sequence	jmod
12	Number of Scans	256
13	Receiver Gain	101
14	Relaxation Delay	2.0000
15	Pulse Width	10.0000
16	Acquisition Time	1.0879
17	Acquisition Date	2020-02-07T11:47:35
18	Modification Date	2020-02-07T06:02:38
19	Spectrometer Frequency	125.78
20	Spectral Width	30120.5
21	Lowest Frequency	-2482.7
22	Nucleus	13C
23	Acquired Size	32768
24	Spectral Size	32768

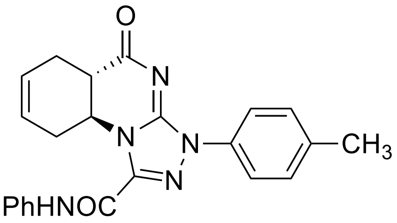

(4a*S**,8a*S**)-9-Oxo-*N*-phenyl-1-(*p*-tolyl)-1,4a,5,8,8a,9-hexahydro-[1,2,4]triazolo[4,3-*a*]quinazoline-3-carboxamide (**5h**)

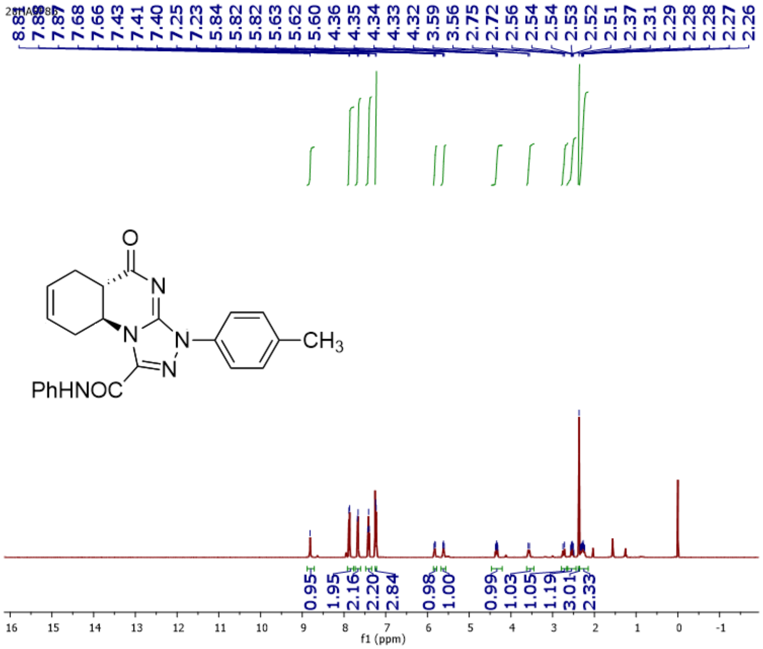

Parameter		Value
1	Data File Name	E:/ after/ Hungary work/ 19 20HAS/ 20HASF/ F8/ 20HASF8B-20 200211T0809 01Z-001/ 20HASF8B/ 10/ pdata/ 1/ 1r
2	Title	20HASF8B
3	Comment	
4	Origin	Bruker BioSpin GmbH
5	Owner	nmrsu
6	Site	
7	Spectrometer	CAB AV4 500 MHZ BASIC
8	Author	
9	Solvent	CDCl3
10	Temperature	310.0
11	Pulse Sequence	zg30
12	Number of Scans	16
13	Receiver Gain	81
14	Relaxation Delay	1.0000
15	Pulse Width	10.8400
16	Acquisition Time	3.2768
17	Acquisition Date	2020-02-10T12:49:06
18	Modification Date	2020-02-11T00:08:50
19	Spectrometer Frequency	500.20
20	Spectral Width	10000.0
21	Lowest Frequency	-1925.1
22	Nucleus	1H
23	Acquired Size	32768
24	Spectral Size	65536

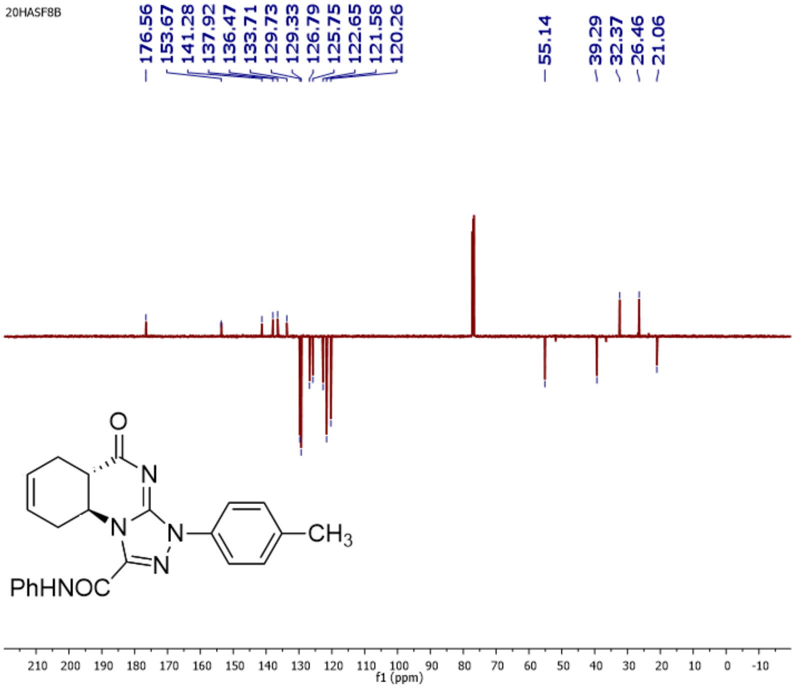

Parameter		Value
1	Data File Name	E:/ after/ Hungary work/ 19 20HAS/ 20HASF/ F8/ 20HASF8B-20200211T080901Z-001/ 20HASF8B/ 11/ pdata/ 1/ 1r
2	Title	20HASF8B
3	Comment	
4	Origin	Bruker BioSpin GmbH
5	Owner	nmrsu
6	Site	
7	Spectrometer	CAB AV4 500 MHZ BASIC
8	Author	
9	Solvent	CDCl3
10	Temperature	310.0
11	Pulse Sequence	jmod
12	Number of Scans	256
13	Receiver Gain	101
14	Relaxation Delay	2.0000
15	Pulse Width	10.0000
16	Acquisition Time	1.0879
17	Acquisition Date	2020-02-10T13:03:31
18	Modification Date	2020-02-11T00:08:56
19	Spectrometer Frequency	125.78
20	Spectral Width	30120.5
21	Lowest Frequency	-2482.7
22	Nucleus	13C
23	Acquired Size	32768
24	Spectral Size	32768

Crystallographic details of compound 5a

Table S1. Crystal Data.

	**5a**
CCDC	
empirical formula	$C_{18}H_{18}N_4O_3$
fw	338.36
temp (K)	120(2)
λ(Å)	1.54184
cryst syst	Triclinic
space group	$P\bar{1}$
a (Å)	9.5332(3)
b (Å)	10.4081(3)
c (Å)	16.8864(5)
α (deg)	89.227(2)
β (deg)	81.130(2)
γ (deg)	74.199(3)
V (Å^3)	1592.20(9)
Z	4
ρ_{calc} (Mg/m^3)	1.412
μ(Mo Kα) (mm^{-1})	0.812
No. reflns.	42738
Unique reflns.	6682
Completeness to θ=67.684°	100%
GOOF (F^2)	1.029
R_{int}	0.0503
R1[a] ($I \geq 2\sigma$)	0.0377
wR2[b] ($I \geq 2\sigma$)	0.0932

[a] $R1 = \Sigma||F_o| - |F_c||/\Sigma|F_o|$. [b] $wR_2 = \{\Sigma[w(F_o^2 - F_c^2)^2]/ \Sigma[w(F_o^2)^2]\}^{1/2}$

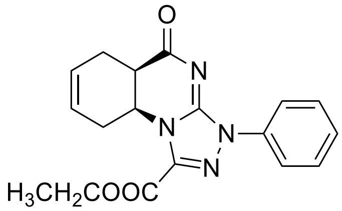

(4a*S**,8a*R**)-Ethyl 9-oxo-1-phenyl-1,4a,5,8,8a,9-hexahydro[1,2,4]triazolo[4,3-*a*]quinazoline-3-carboxylate (**4a**)

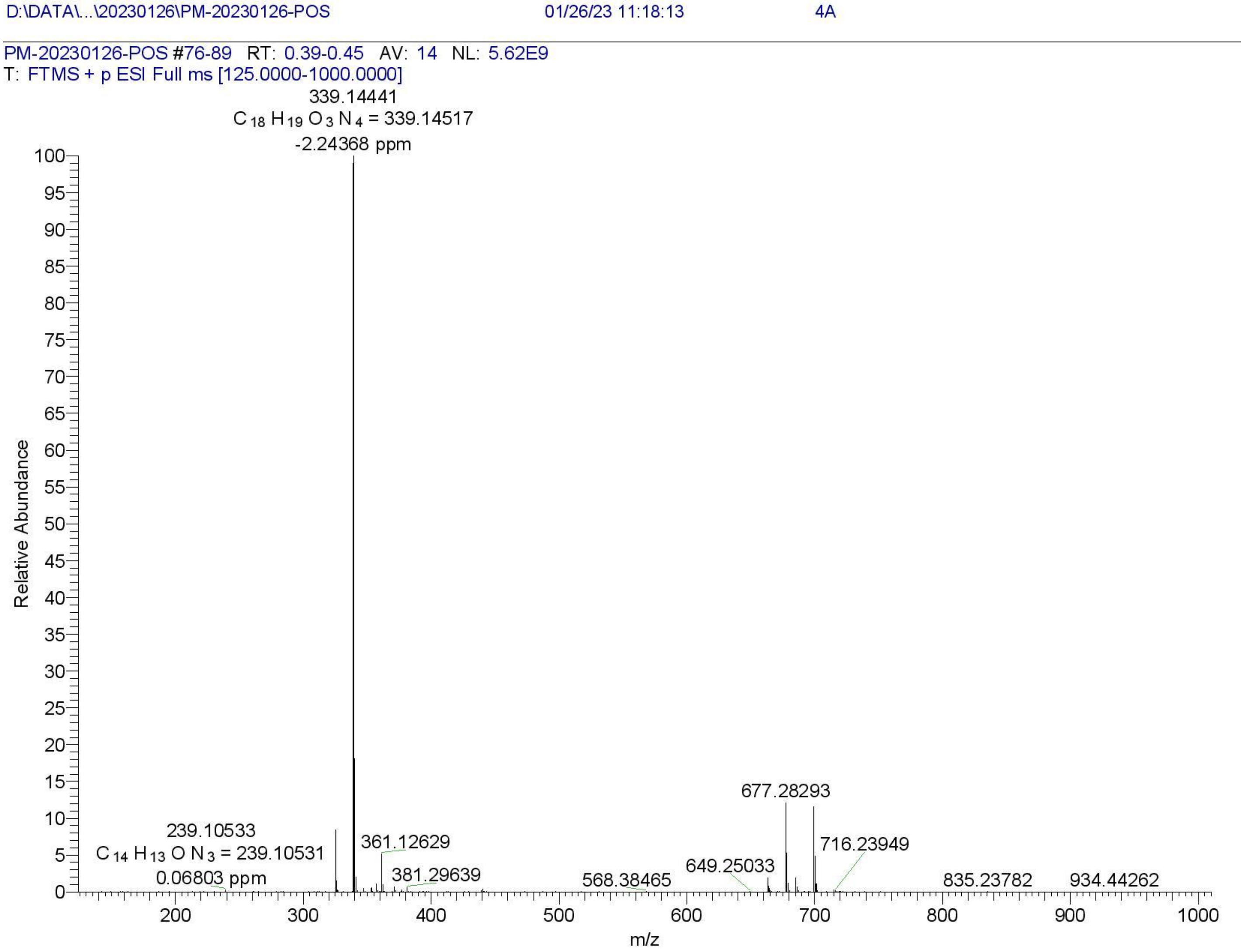

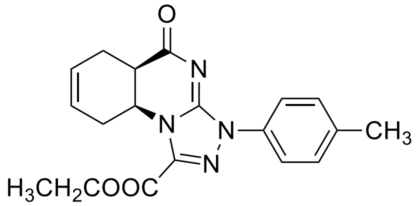

(4a*S**,8a*R**)-Ethyl 9-oxo-1-(*p*-tolyl)-1,4a,5,8,8a,9-hexahydro[1,2,4]triazolo[4,3-*a*]quinazoline-3-carboxylate (**4b**)

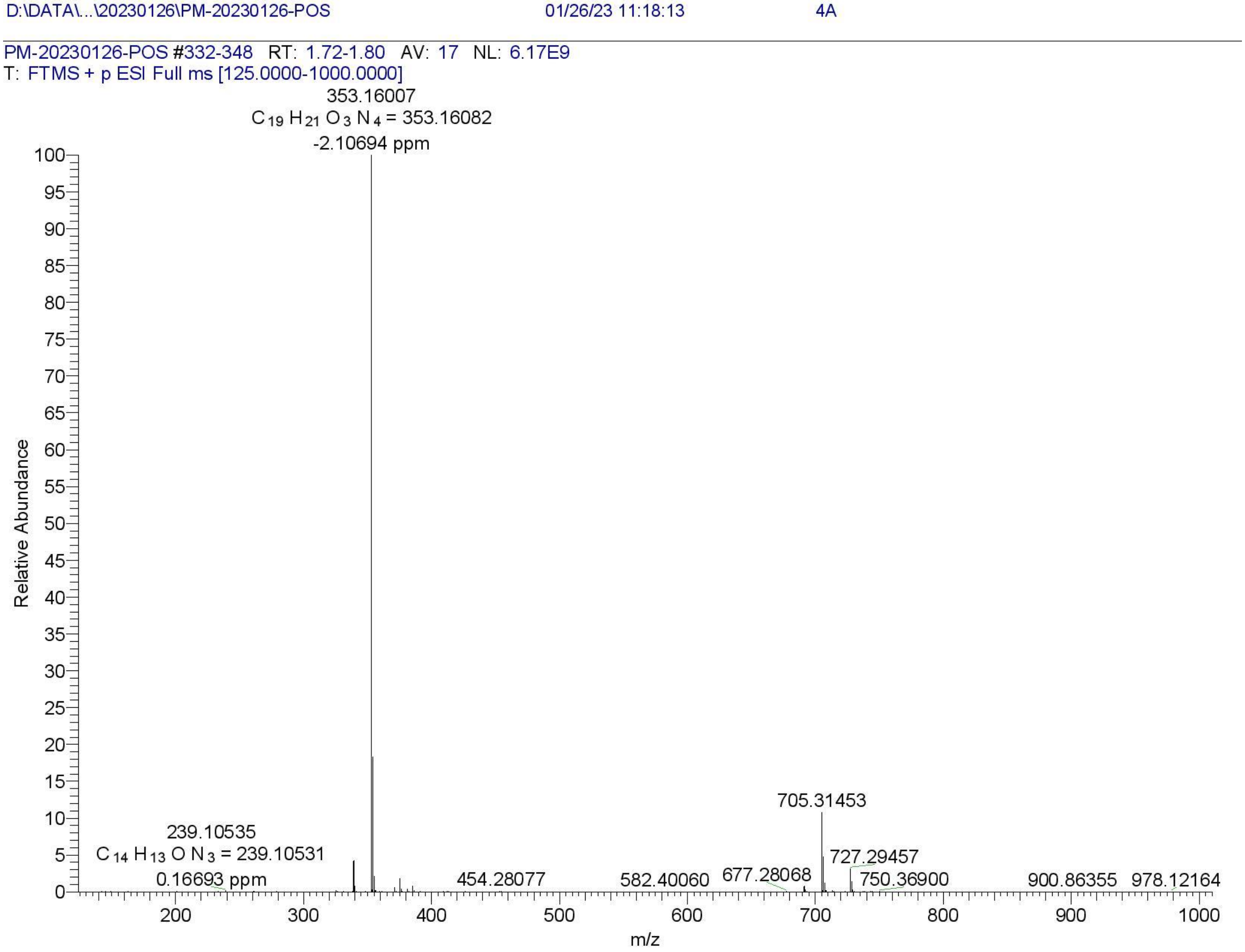

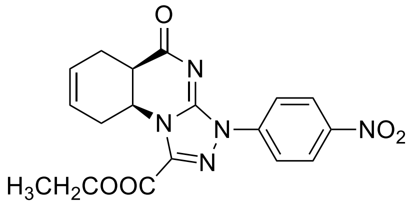

(4a*S**,8a*R**)-Ethyl 9-oxo-1-(4-nitrophenyl)-1,4a,5,8,8a,9-hexahydro-[1,2,4]triazolo[4,3-*a*]quinazoline-3-carboxylate (**4c**)

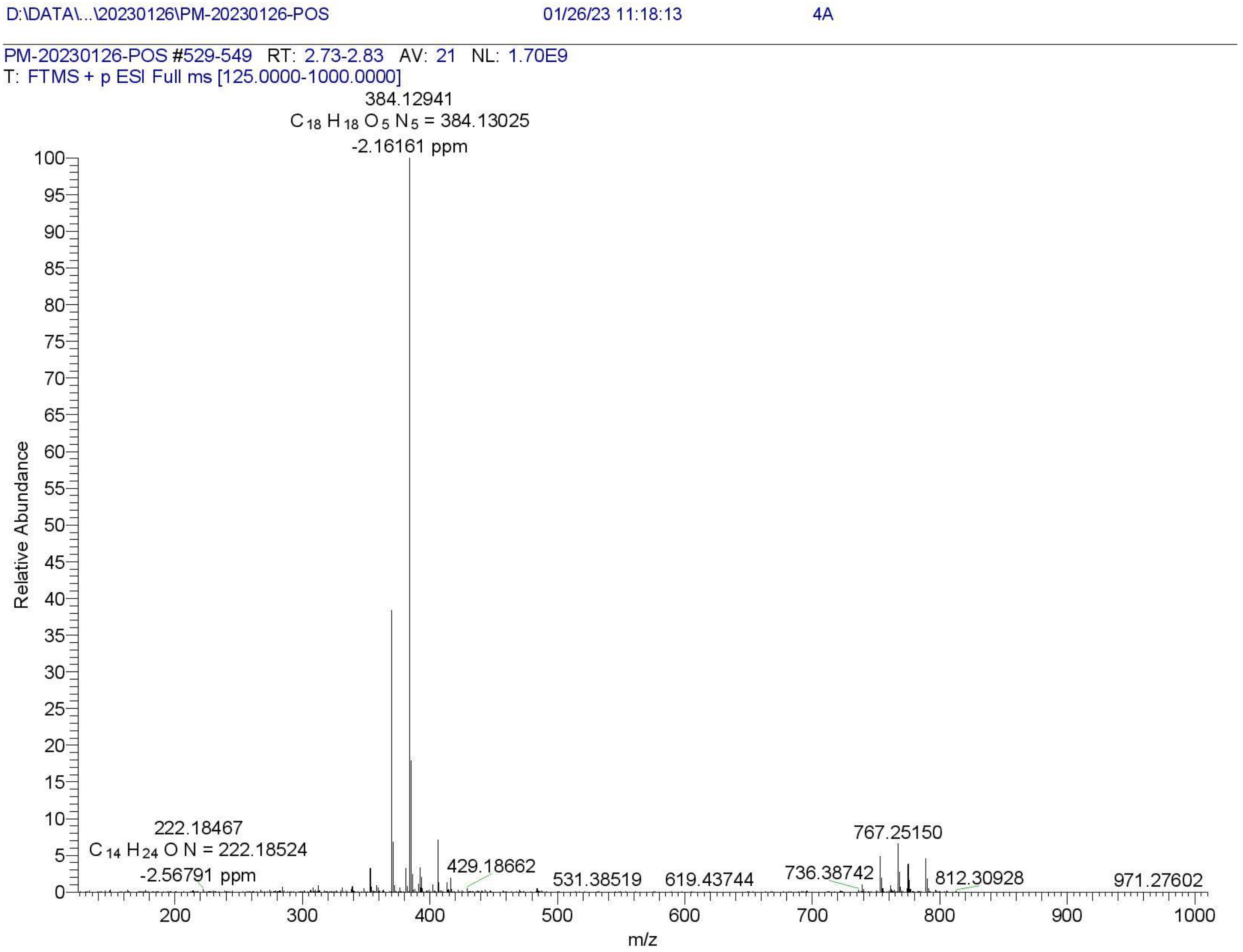

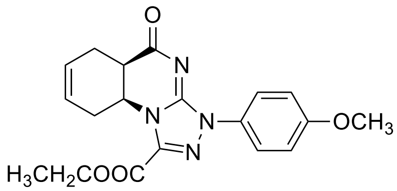

(4a*S**,8a*R**)-Ethyl 9-oxo-1-(4-methoxyphenyl)-1,4a,5,8,8a,9-hexahydro[1,2,4]triazolo[4,3-*a*]quinazoline-3-carboxylate (**4d**)

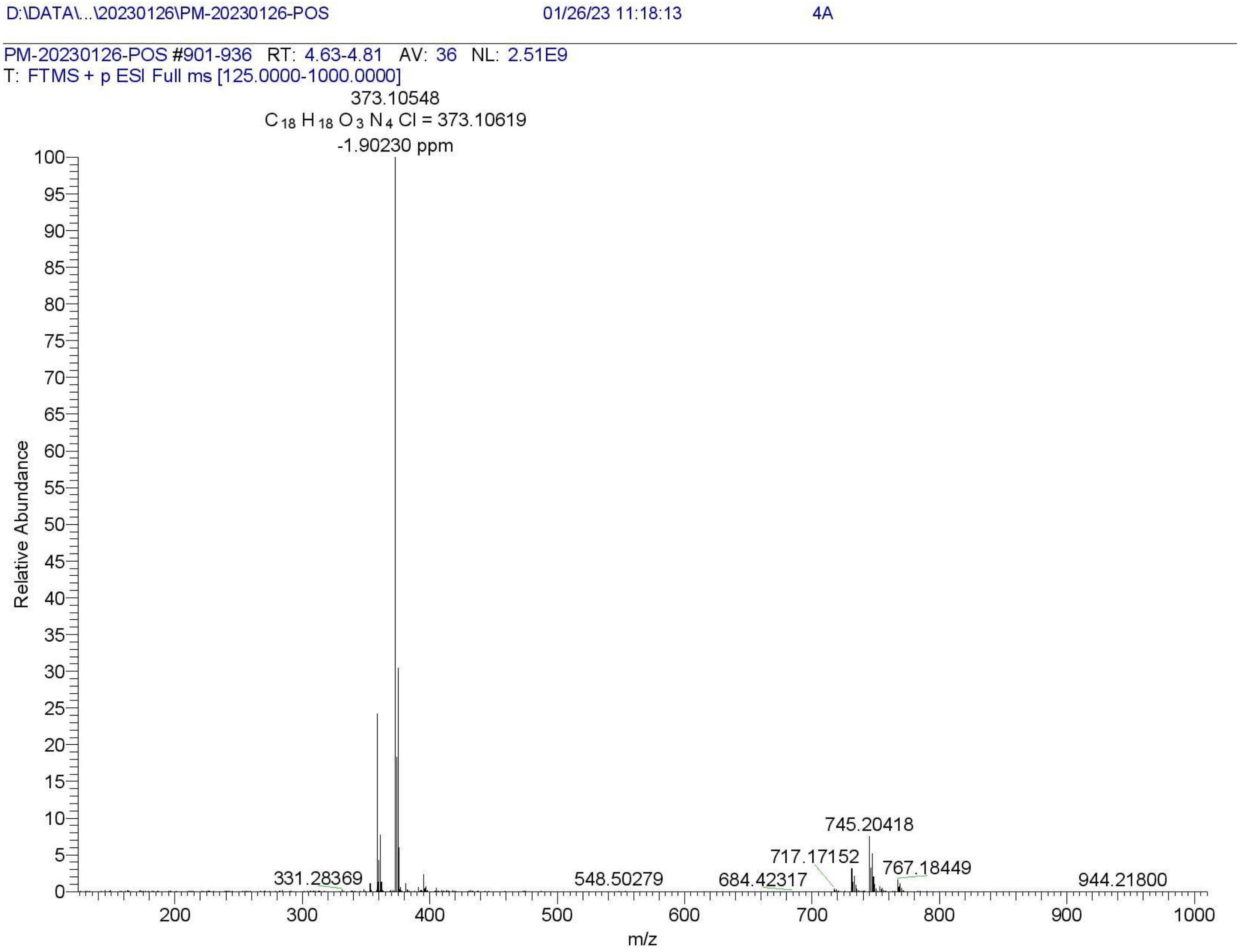

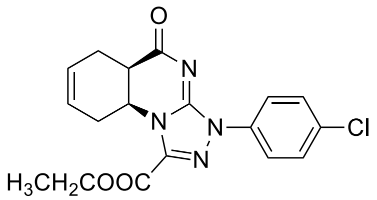

(4a*S**,8a*R**)-Ethyl 9-oxo-1-(4-chlorophenyl)-1,4a,5,8,8a,9-hexahydro[1,2,4]triazolo[4,3-*a*]quinazoline-3-carboxylate (**4e**)

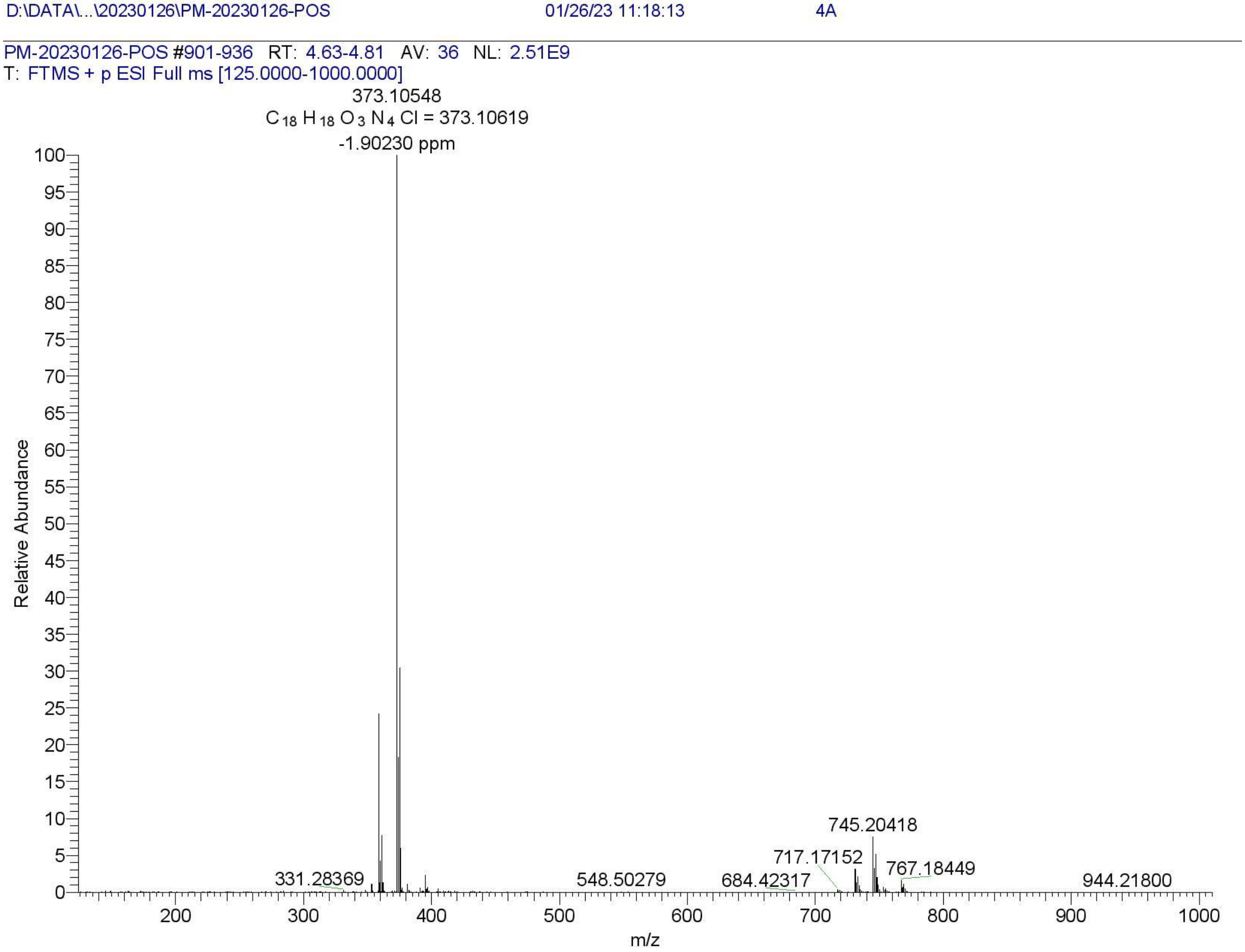

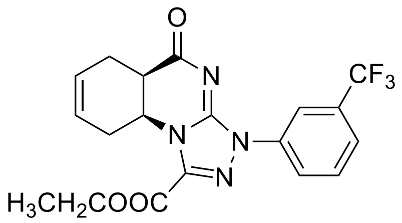

(4a*S**,8a*R**)-Ethyl 9-oxo-1-(3-(trifluoromethyl)phenyl)-1,4a,5,8,8a,9-hexahydro[1,2,4]triazolo[4,3-*a*]quinazoline-3-carboxylate (**4f**)

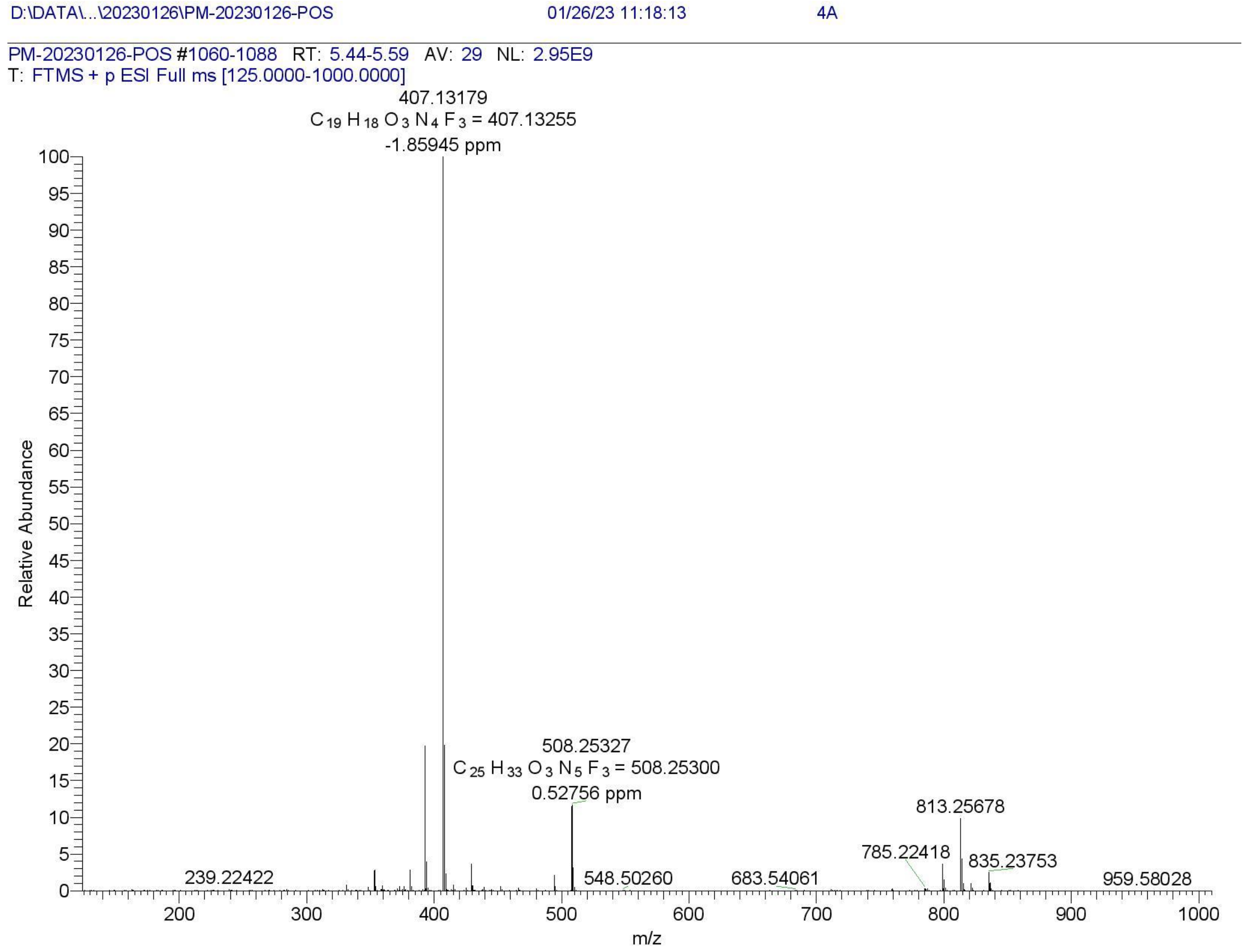

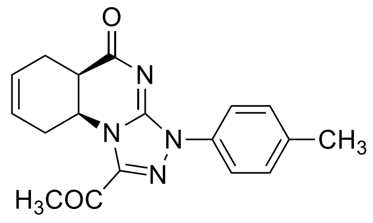

(4a*S**,8a*R**)-3-Acetyl-1-(*p*-tolyl)-4a,5,8,9-tetrahydro[1,2,4]triazolo[4,3-*a*]quinazoline-9(1*H*)-one (**4g**)

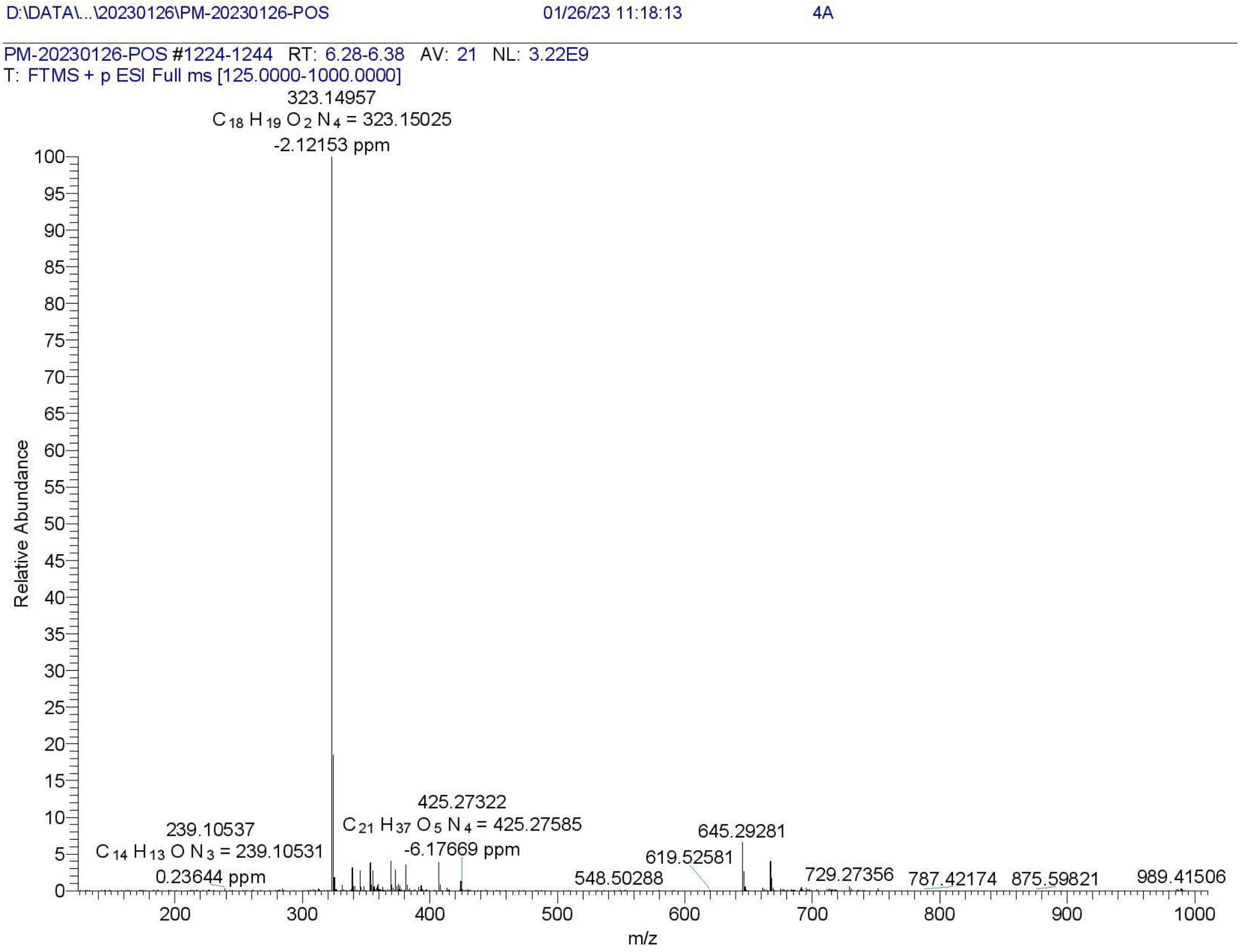

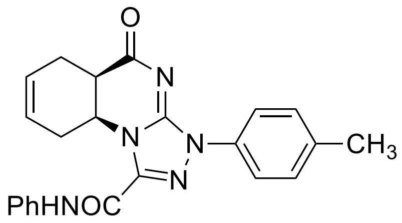

(4a*S**,8a*R**)-9-Oxo-*N*-Phenyl-1-(*p*-tolyl)-1,4a,5,8,8a,9-hexahydro[1,2,4]triazolo[4,3-*a*]quinazoline-3-carboxamide (**4h**)

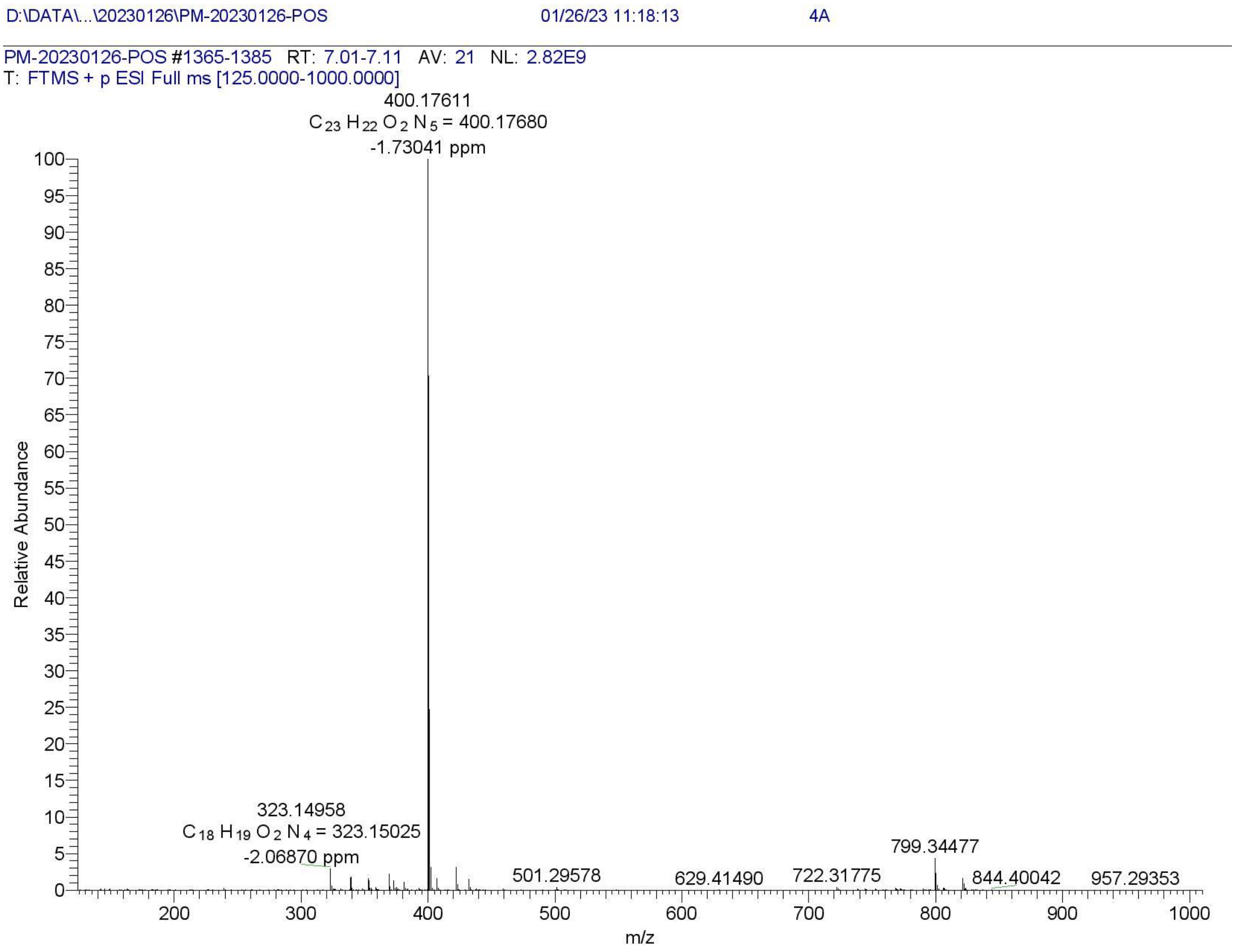

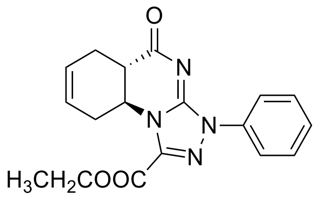

(4a*S**,8a*S**)-Ethyl 9-oxo-1-phenyl-1,4a,5,8,8a,9-hexahydro-[1,2,4]triazolo[4,3-a]quinazoline-3-carboxylate (**5a**)

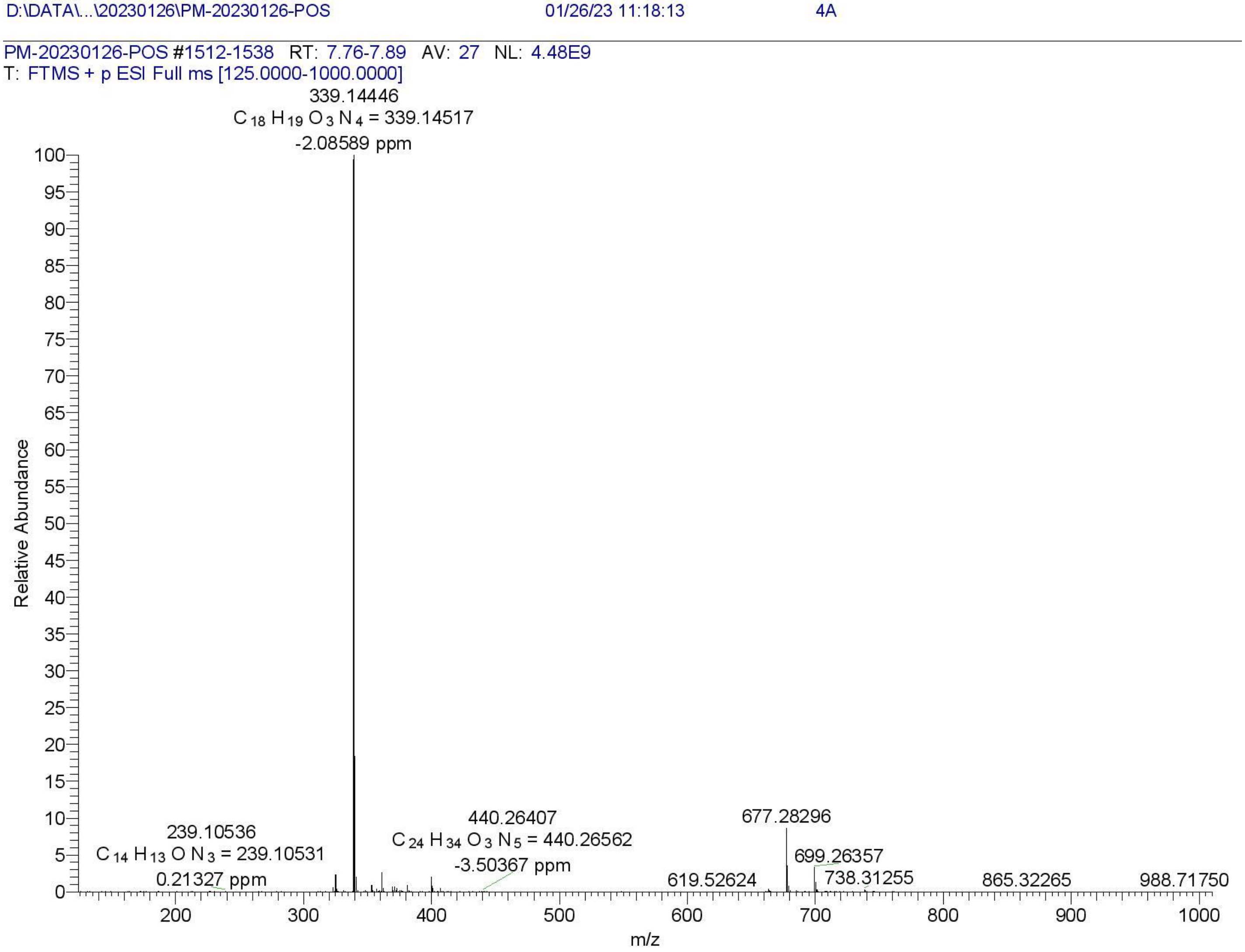

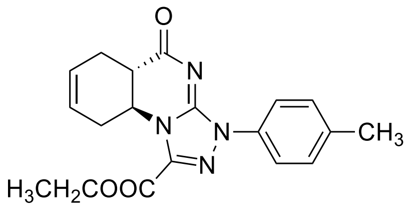

(4a*S**,8a*S**)-Ethyl 9-oxo-1-(*p*-tolyl)-1,4a,5,8,8a,9-hexahydro-[1,2,4]triazolo[4,3-*a*]quinazoline-3-carboxylate (**5b**)

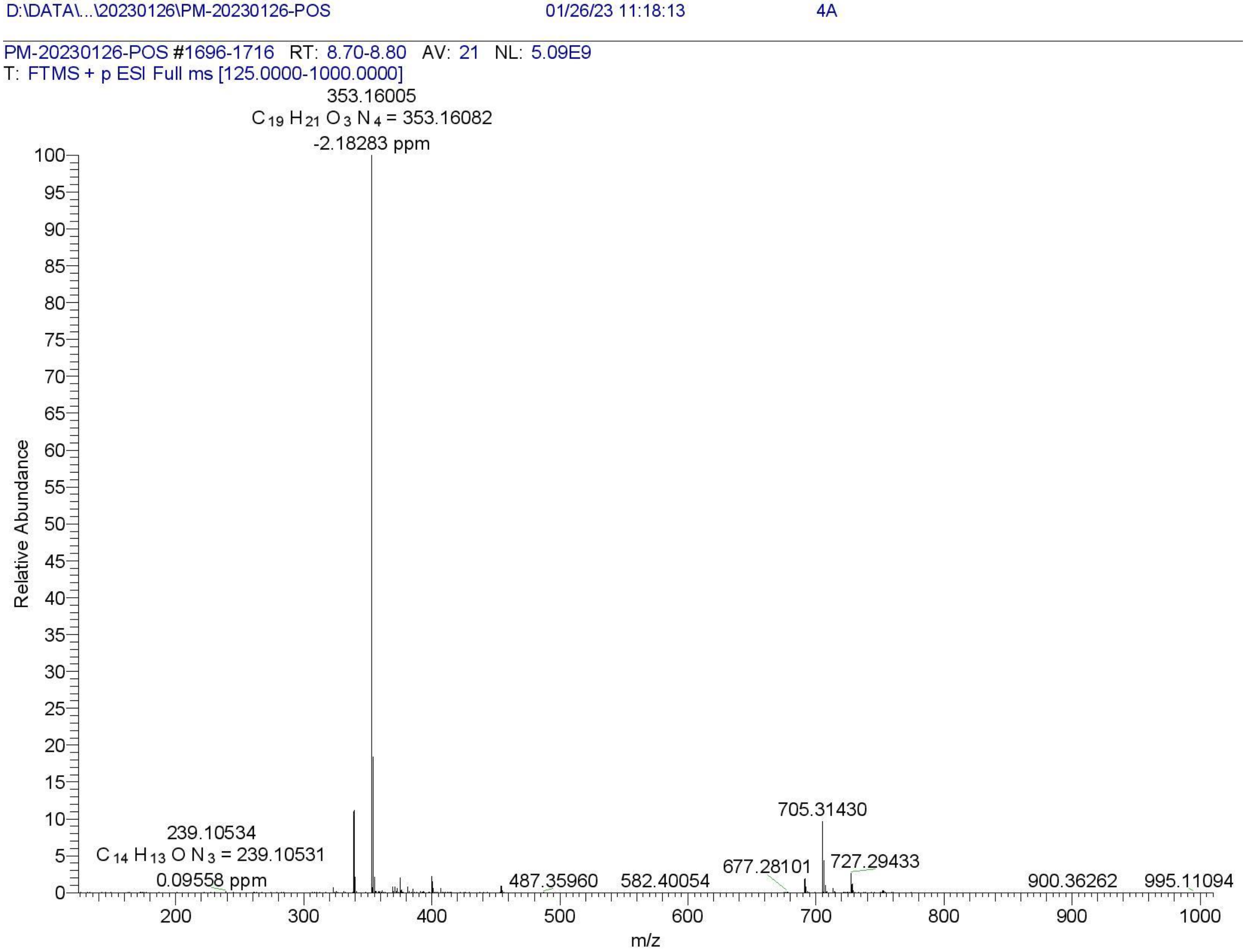

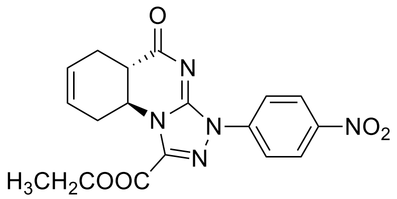

(4a*S**,8a*S**)-Ethyl 1-(4-nitrophenyl)-9-oxo-1,4a,5,8,8a,9-hexahydro-[1,2,4]triazolo[4,3-*a*]quinazoline-3-carboxylate (**5c**)

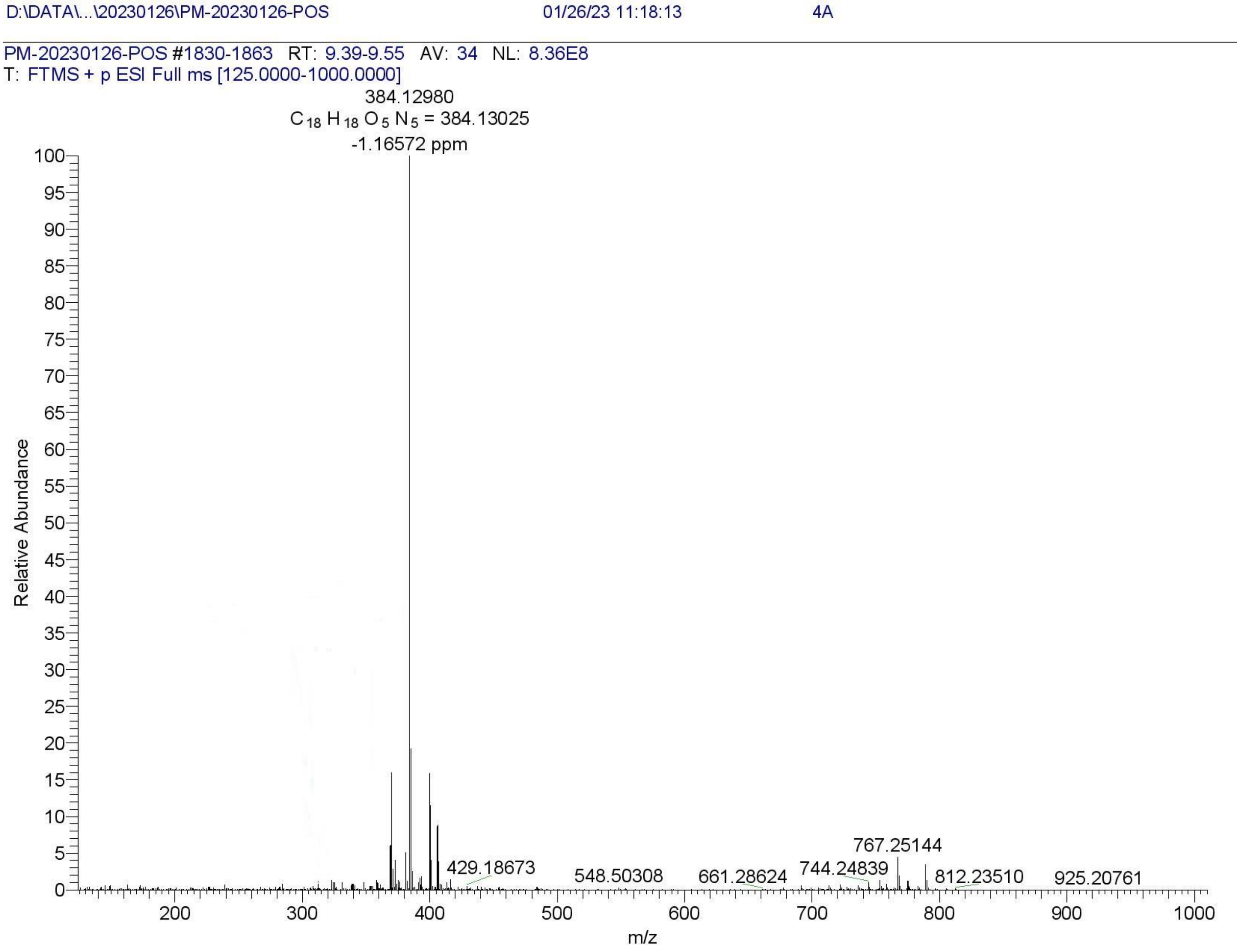

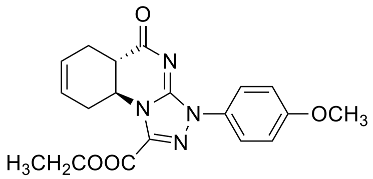

(4a*S**,8a*S**)-Ethyl 1-(4-methoxyphenyl)-9-oxo-1,4a,5,8,8a,9-hexahydro-[1,2,4]triazolo[4,3-*a*]quinazoline-3-carboxylate (**5d**)

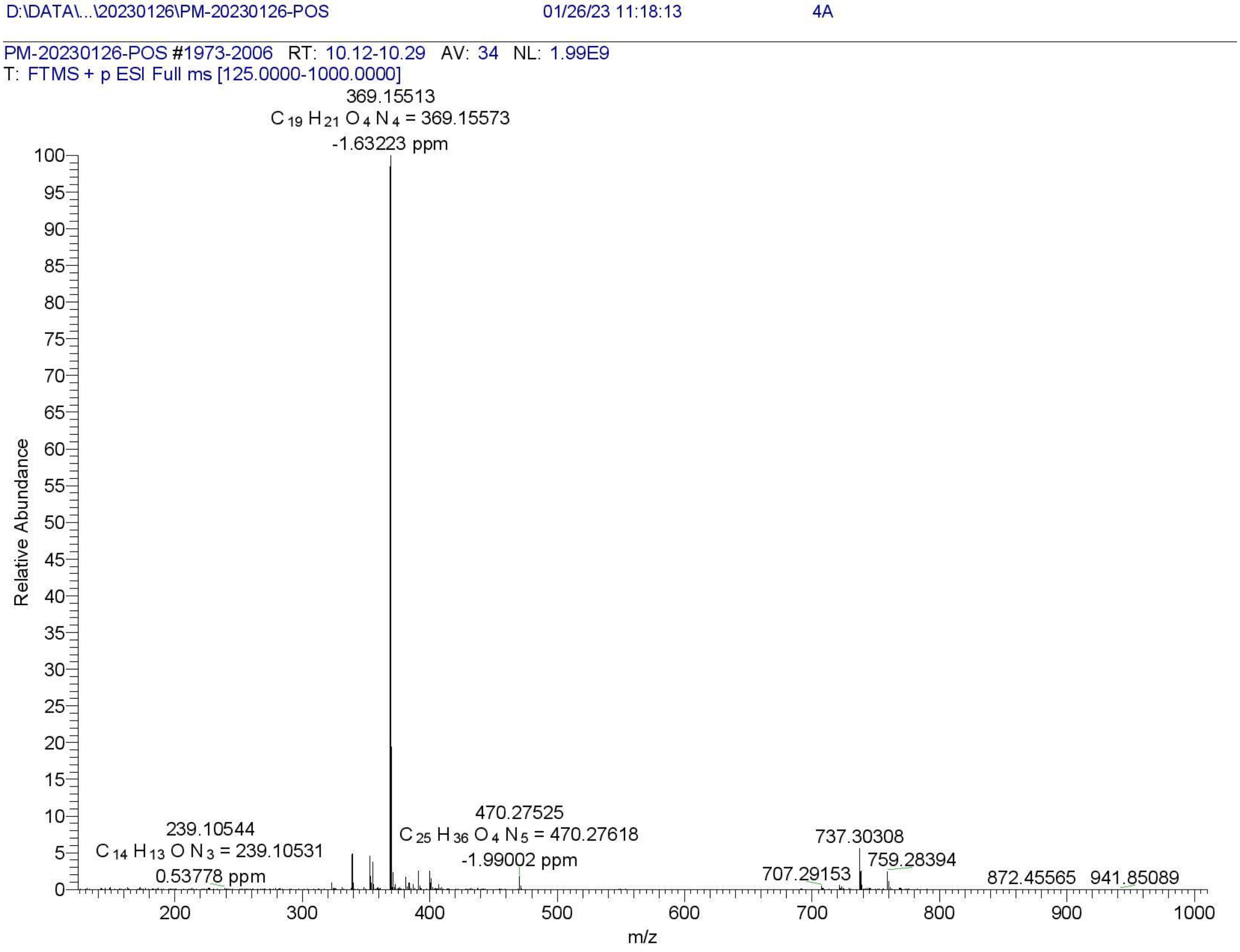

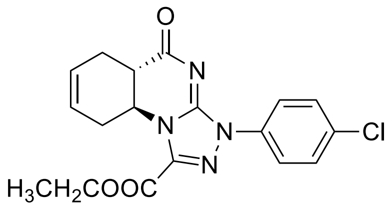

(5a*S**,9a*S**)-Ethyl 3-(4-chlorophenyl)-5-oxo-3,5,5a,6,9,9a-hexahydro-[1,2,4]triazolo[4,3-*a*]quinazoline-1-carboxylate (**5e**)

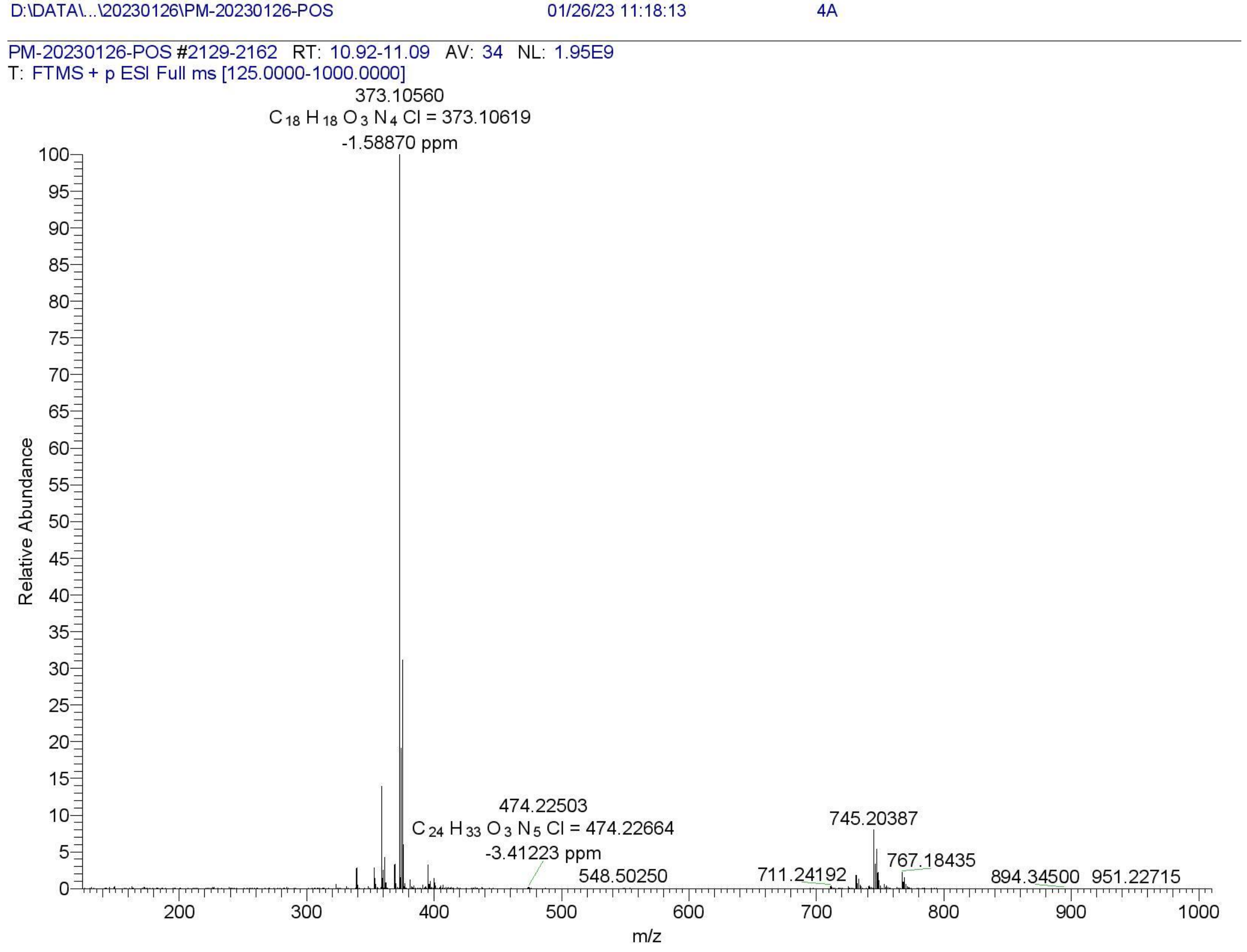

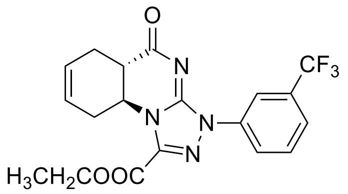

(4a*S**,8a*S**)-Ethyl 9-oxo-1-(3-(trifluoromethyl)phenyl)- 1,4a,5,8,8a,9-hexahydro-[1,2,4]triazolo[4,3-*a*]quinazoline-3-carboxylate (**5f**)

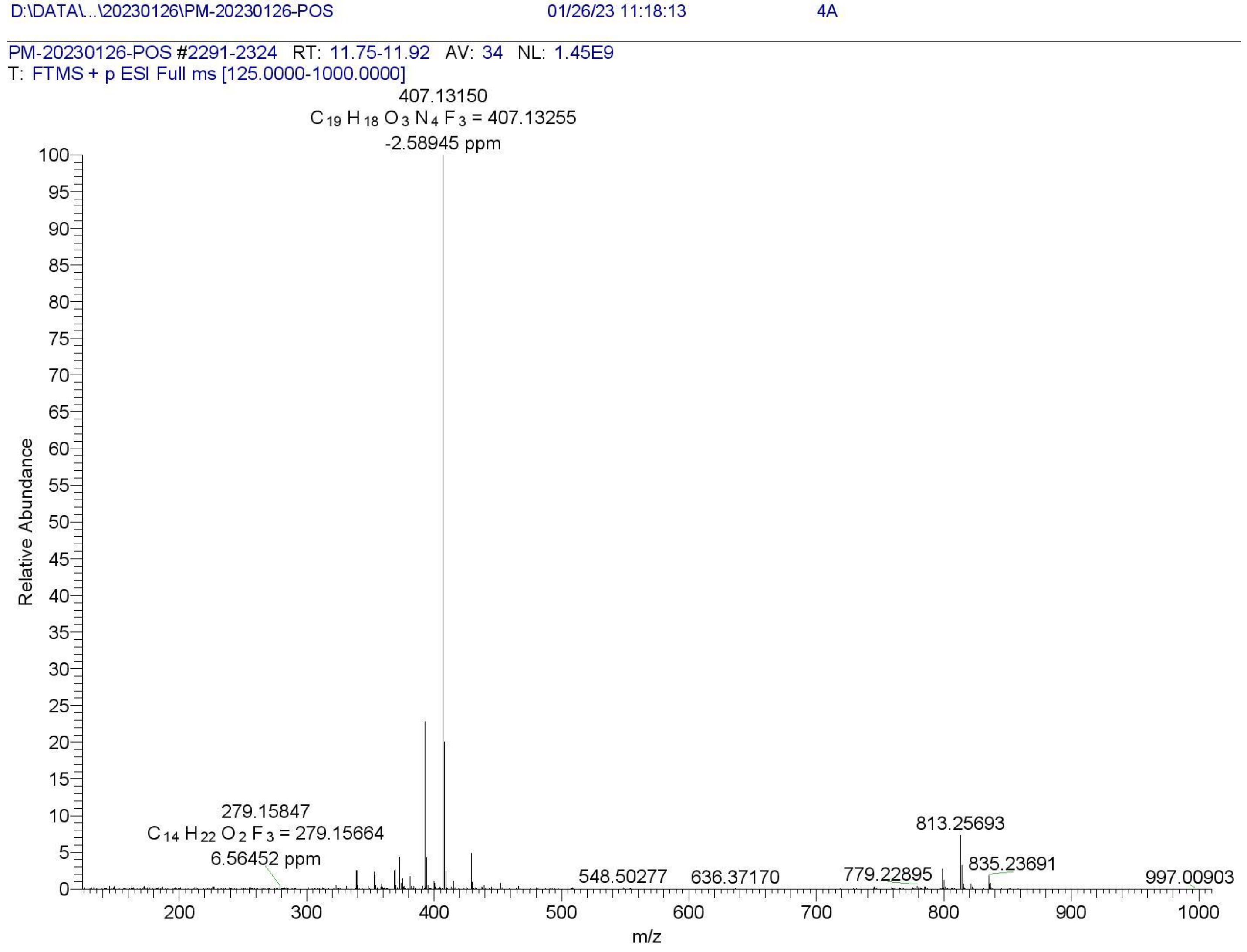

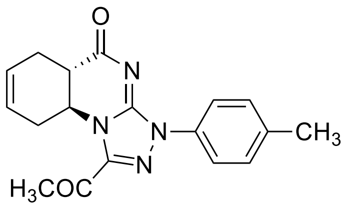

(4a*S**,8a*S**)-3-Acetyl-1-(*p*-tolyl)-4a,5,8,9-tetrahydro-[1,2,4]triazolo[4,3-*a*]quinazolin-9(1*H*)-one (**5g**)

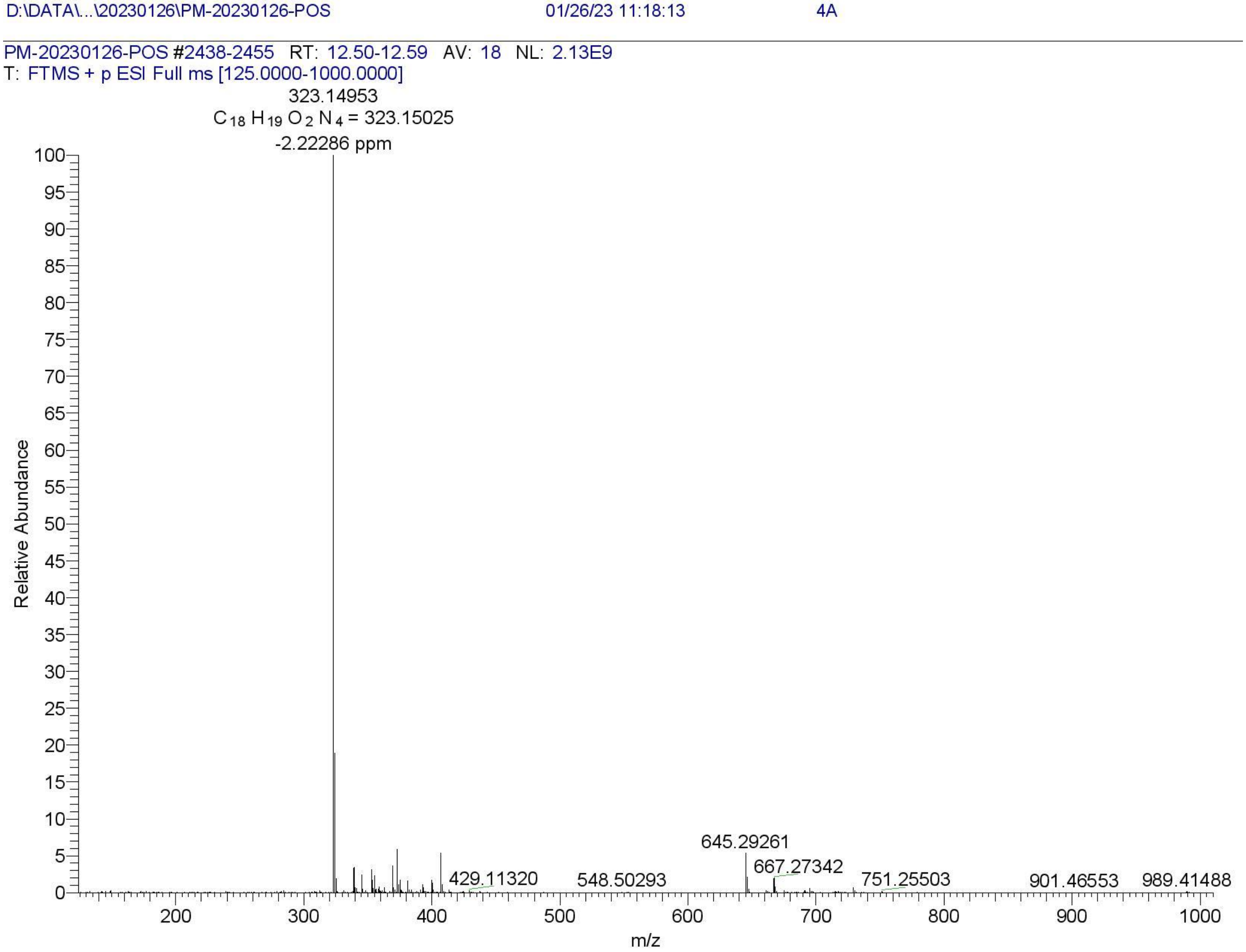

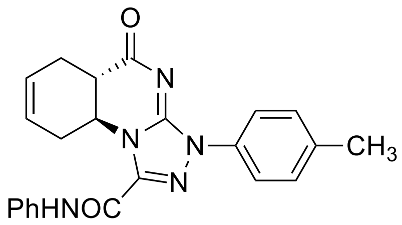

(4a*S**,8a*S**)-9-Oxo-*N*-phenyl-1-(*p*-tolyl)-1,4a,5,8,8a,9-hexahydro-[1,2,4]triazolo[4,3-*a*]quinazoline-3-carboxamide (**5h**)

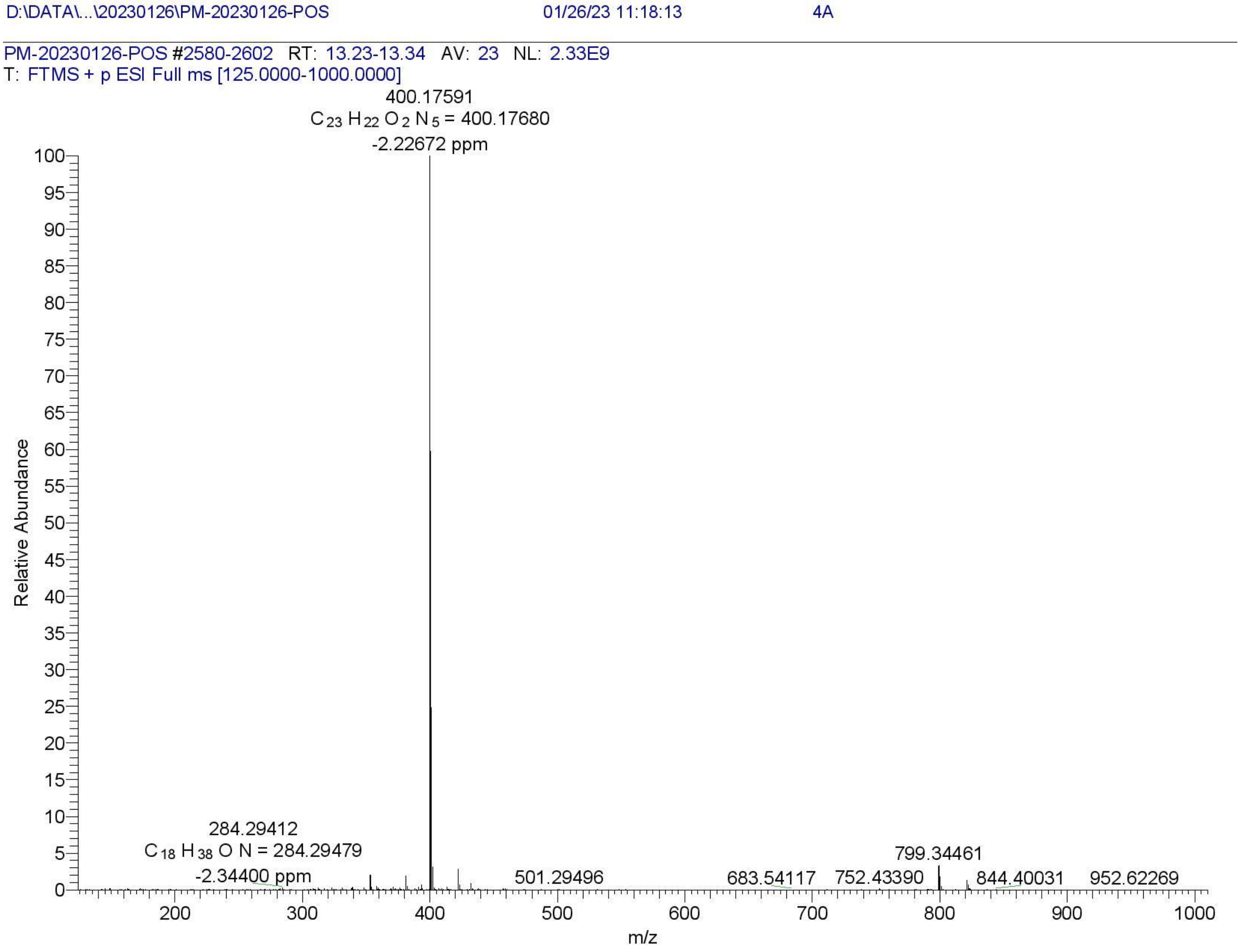

Supplement: Supplementary file 1 [file molecules-28-03718-s001.zip › molecules-2370099-supplementary.pdf]
